# Exploring medium and long arm extensions of 1,2,4-triazole derivatives as *Candida albicans* 14α-demethylase (CYP51) inhibitors†

**DOI:** 10.1039/d4md00863d

**Published:** 2025-03-12

**Authors:** Marwa Alsulaimany, Faizah A. Binjubair, Esra Tatar, Diane E. Kelly, Steven L. Kelly, Andrew G. Warrilow, Mikhail V. Keniya, Brian C. Monk, Josie E. Parker, Claire Simons

**Affiliations:** a School of Pharmacy and Pharmaceutical Sciences, Cardiff University King Edward VII Avenue Cardiff CF10 3NB UK simonsc@cardiff.ac.uk; b Department of Pharmaceutical Chemistry, Faculty of Pharmacy, Marmara University 34668 Istanbul Turkey; c Centre for Cytochrome P450 Biodiversity, Institute of Life Science, Swansea University Medical School, Swansea University Swansea SA2 8PP UK; d Faculty of Dentistry, Sir John Walsh Research Institute, University of Otago Dunedin 9016 New Zealand; e School of Biosciences, Cardiff University Museum Avenue Cardiff CF10 3AX UK parkerj21@cardiff.ac.uk

## Abstract

Fungal infections have been described as a silent crisis affecting more than one billion people each year. At least 150 million of these cases involve severe and life threatening invasive fungal infections, accounting for approximately 1.7 million deaths annually. 1,2,4-Trizoles such as fluconazole and posaconazole are widely used antifungal agents, but azole resistance is an increasing problem requiring further study. 1,2,4-Triazole derivatives with medium and long arm extensions designed to bind within the *Candida albicans* CYP51 (CaCYP51) access channel were synthesised to study their inhibition of CaCYP51 (IC_50_, MIC) and binding affinity (*K*_d_). A long arm extension using the amide linker was found to be most effective (*e.g.*13), giving an antifungal profile *vs.* wild-type and resistant model fungal strains comparable with posaconazole.

## Abstract 2

1,2,4-trizole derivatives were designed to study their inhibition of *Candida albicans* CYP51 (IC_50_), binding affinity (*K*_d_) and antifungal profile *vs.* wild-type and resistant model fungal strains comparable with posaconazole.

## Graphical abstract

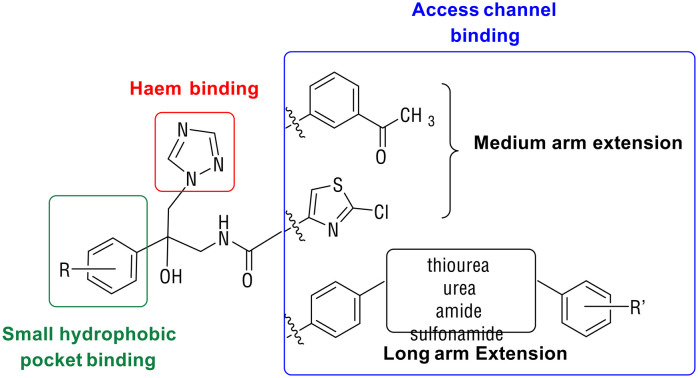

## Introduction

*Candida albicans* (*C. albicans*), the predominant cause of candidiasis, is a commensal that resides in different parts of the human body including the gastrointestinal tract, genitourinary tract, oropharynx, and the skin.^1^ Under certain conditions *C. albicans* can cause infections that range from superficial to invasive, life-threatening, infections such as candidemia.^1,2^ Worldwide *C. albicans* is one of the most common healthcare associated bloodstream infections in intensive care units with a mortality of 30–50% for candidemia.^3^

According to the Centre for Disease Control and prevention (CDC), many patients with severe COVID-19 symptoms in 2020 developed invasive candidemia from *C. albicans* as well as resistance to antifungal treatment owing to immune suppression caused by the virus.^4–6^ This emphasises the need for improved antifungal treatments as the current range of effective antifungal agents is limited and susceptible to drug resistance.

Ergosterol is a fungal-specific and major sterol component of the fungal cell membrane that affects its physiochemical characteristics including fluidity. A key cytochrome P450 (CYP) enzyme involved in the synthesis of ergosterol is sterol 14α-demethylase (CYP51, Erg11).^7^ The azole antifungals are the largest class of drugs that act by inhibiting CYP51.^8^ They are type II inhibitors that bind directly with the haem iron. The incidence of drug resistance to the available azole antifungals in yeast and other fungi is increasing and is attributed to the long treatments and prophylactic use in the clinic, and widespread use of agricultural azole fungicides to protect crops.^9,10^

**Fig. 1:**
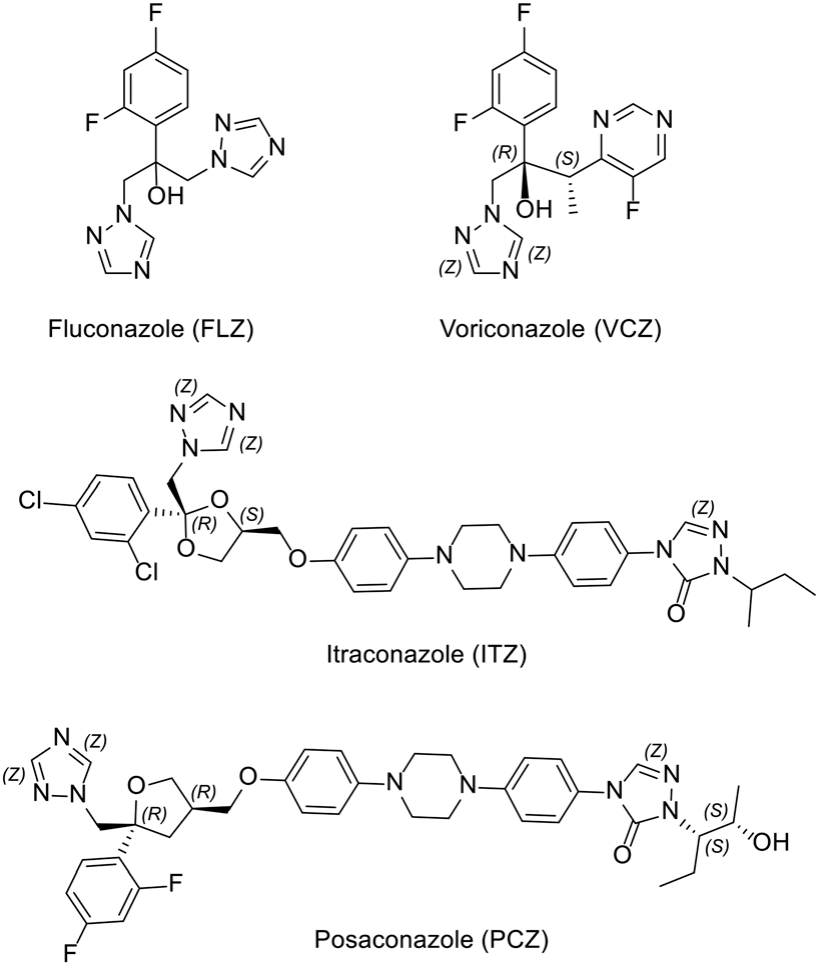
Structure of clinically used antifungal agents.

Fluconazole (FLZ, Fig. 1) is one of the most commonly used antifungal agents and significant target-based FLZ resistance has been found in clinical isolates of *C. albicans*.^10–14^ FLZ resistance has been attributed to single and double mutations within CYP51, with common single amino acid mutations involving Y132F and K143R located within the haem active site, and a G450E mutation within the electron transfer area that interacts with the NADPH-cytochrome P450 reductase redox partner. Y132 forms a key H-bonding interaction with azole antifungals *via* the tertiary hydroxy group of triazoles such as FLZ and voriconazole (VCZ, Fig. 1). Double mutations such as *C. albicans* CYP51 (CaCYP51) Y132H/K143R and Y132F/F145L further reduce the binding affinity of FLZ, increase the IC_50_ value,^10^ and probably account for increased MIC values found in *C. albicans* strains containing these mutations.^11–14^ Conformational changes induced by such mutations are also likely to affect the binding between the azole nitrogen and the haem iron as well as the haem propionates with Y118 and Y132, *e.g.* by affecting H-bonding interactions of haem and by distorting the ionic interaction with K143.^9,10,15–18^

Azole antifungals, especially the triazoles, are often a first-choice treatment due to their high oral bioavailability, low toxicity, selectivity, tolerability by patient and the broad spectrum of activity. There are some limitations for the use of these azoles. For example, while VCZ (Fig. 1) has a broad spectrum of activity against most *Candida* species and fungal pathogens compared with FLZ, it has more adverse effects and drug–drug interactions than FLZ.^19^ Itraconazole (ITZ, Fig. 1) and posaconazole (PCZ, Fig. 1) have broad spectrum activities against candidiasis, but their low bioavailability has limited their clinical use.^20^

**Fig. 2:**
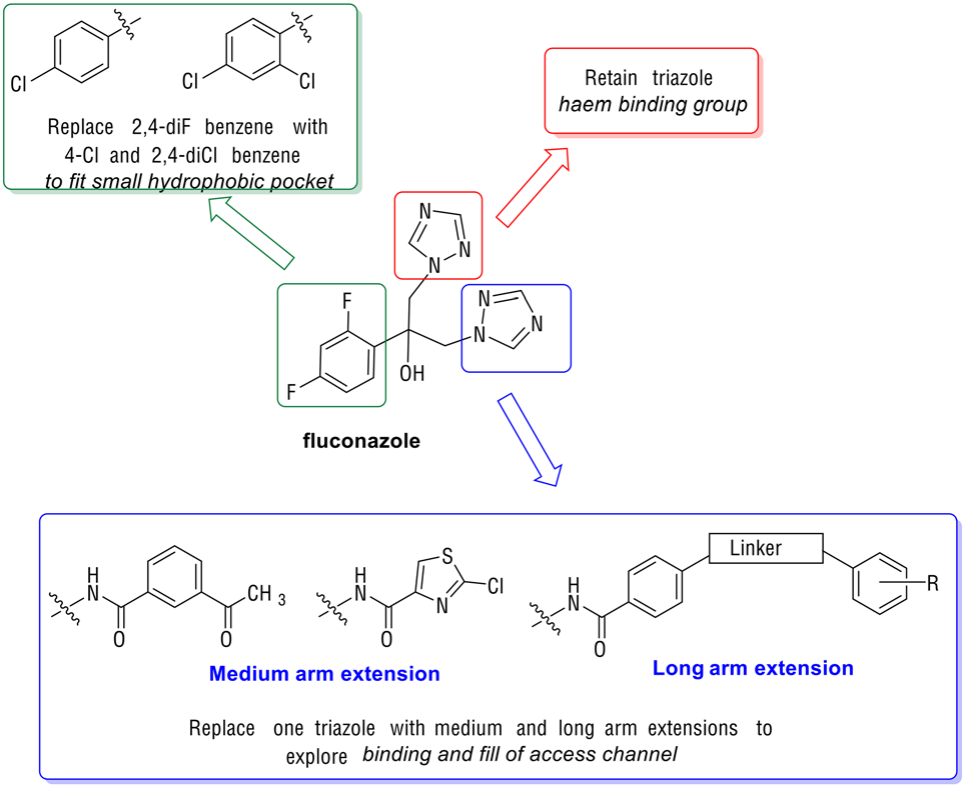
Rationale for modifications to generate azole antifungals with medium and long arm extensions.

Recent studies on azole antifungals^21^ have focussed on azole-hybrid structures *e.g.* FLZ-COX inhibitor hybrids,^22^ to explore dual activity and tetrazole derivatives *e.g.* VT-1161 (oteseconazole),^23^ to improve CYP selectivity, althoughVT-1161 is susceptible to azole resistance.^24^ The increased incidence of resistant clinical isolates highlights the importance of developing new antifungals with activity against such strains, with azoles the focus of the research presented here. Our goal was to develop mid-sized and extended arm azoles targeting the CaCYP51 enzyme by using FLZ as a pharmacophore and applying the promising results obtained in our previous published study^17^ to enhance activity, in particular against resistant fungal strains, and binding affinity. The following design was proposed: one triazole moiety of FLZ was retained as the haem-binding group and the 2,4-difluorobenzene ring was replaced with the more lipophilic 4-chlorobenzene and 2,4-dichlorobenzene to enhance uptake across the lipophilic fungal cell wall and still fit in the small hydrophobic binding site of CaCYP51. The second triazole ring was replaced with either a medium or long arm extension (Fig. 2). The medium extensions contain an amide linked to either acetophenone or a 2-chlorothiazole ring identified through computational modelling to increase binding interactions. The long arm extensions contain a benzamide attached to a second benzene ring through either a thiourea, urea, amide or sulfonamide linker. The rationale for adding thiourea, urea, amide or sulfonamide linkers was to add H-bond acceptor and/or donor capability, which might form additional binding interactions with amino acid residues in the CaCYP51 access channel binding site, to compensate for the loss of the key H-bonding interaction between the azole tertiary hydroxy group and common active site mutations, such as Y132H/F (*C. albicans*) or Y140H/F (*S. cerevisiae models*) in resistant strains.

## Results and discussion

### Chemistry

**Scheme 1:**
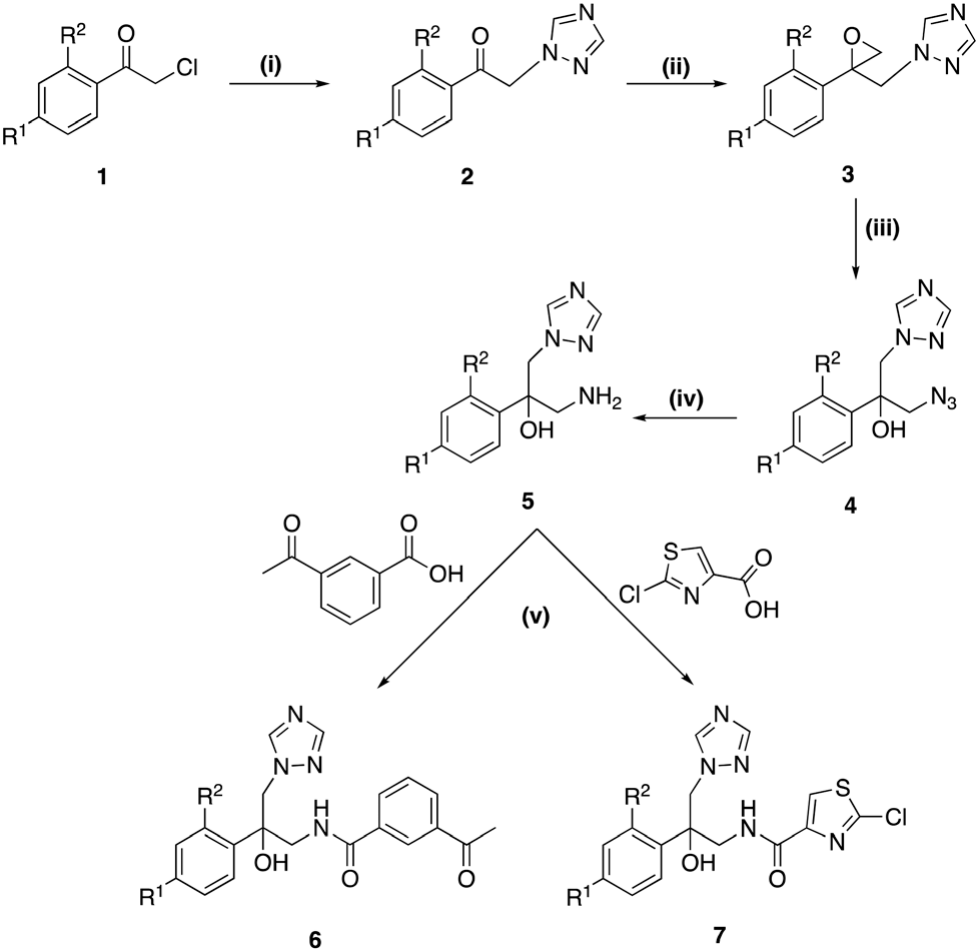
Reagents and conditions: (i) 1,2,4-triazole, K_2_CO_3_, CH_3_CN, 0–5 °C 30 min, r.t., 24 h or 80 °C, 4 h, 51–74% (ii) TMSOI, 2 M aq. NaOH, toluene, 60 °C, 6 h, quantitative crude yield (iii) NaN_3_, NH_4_Cl, DMF, 60 °C, 2 h, r.t., o/n, 65–66% (iv) (a) Ph_3_P, THF, r.t., 1 h (b) H_2_O, 60 °C, 4 h, 60–77% (v) CDI, DMF, r.t., o/n, 45–68% [a R^1^ = Cl, R^2^ = H; b R^1^ = Cl, R^2^ = Cl].

The mid-sized acetyl (6a–b) and thiazole (7a–b) derivatives were obtained by a 5-step synthesis (Scheme 1) starting with introduction of the triazole ring by reaction of acetophenone (1) with 1,2,4-triazole and activated K_2_CO_3_ to give the 1-(substituted phenyl)-2-(1*H*-1,2,4-triazol-1-yl)ethan-1-one (2) derivatives.^25^ Corey-Chaykovsky epoxidation reaction of the ethanone derivative (2) using TMSOI and 20% aqueous NaOH with heating at 60 °C for 6 h (ref. 26) gave the crude epoxides (3), which were directly converted to the azides (4) on treatment with NaN_3_ and NH_4_Cl in good yields (4a 60% and 4b 63%) over two steps from 2. Azide reduction to the free amines (5) was performed using a Staudinger reaction,^27^ with initial reaction of azides (4) with triphenylphosphine in THF at room temperature for 1 h followed by the addition of H_2_O and heating at 60 °C for 4 h. The mid-sized derivatives (6 and 7) were prepared by reaction of the amines (5) with either 3-acetylbenzoic acid or 2-chlorothiazole-4-carboxylic acid using 1,1′-carbonyldiimidazole (CDI) as the amide coupling reagent (Scheme 1). The mid-sized amides (6a–b and 7a–b) were obtained in yields ranging from 45–68% (Table 1) and structure and purity confirmed by ^1^H/^13^C NMR and HPLC respectively.

**Table 1:** Yields and melting points, ^1^H and ^13^C NMR of mid-sized (6 and 7) and linker (11 thiourea, 12 urea, 13 amide and 14 sulfonamide) of derivatives

Cmpd^a^	Yield (%)	MP (°C)	Amide/linker ^1^H NMR (*δ*)^b^	Amide/linker ^13^C NMR (*δ*)^b^
6a	52	Semisolid	NH 8.55	C=O 167.1
6b	45	160–162	NH 8.72	C=O 167.5
7a	68	Semisolid	NH 8.10	C=O 160.1
7b	68	Semisolid	NH 8.26	C=O 160.4
11a	68	146–148	2× NH 10.05, 10.00	C=S 180.0
11b	56	138–140	2× NH 10.06, 10.03	C=S 179.9
12a	63	226–228	2× NH 8.99, 8.90	C=O 152.63
12b	52	216–218	2× NH 9.06, 8.97	C=O 152.66
13a	80	222–224	NH 10.50	C=O 165.13
13b	76	190–192	NH 10.51	C=O 165.14
14a	77	236–238	NH 10.73	—
14b	83	228–230	NH 10.76	—

a a R^1^ = Cl, R^2^ = H; b R^1^ = Cl, R^2^ = Cl.

b DMSO-d_6_*.*

**Scheme 2:**
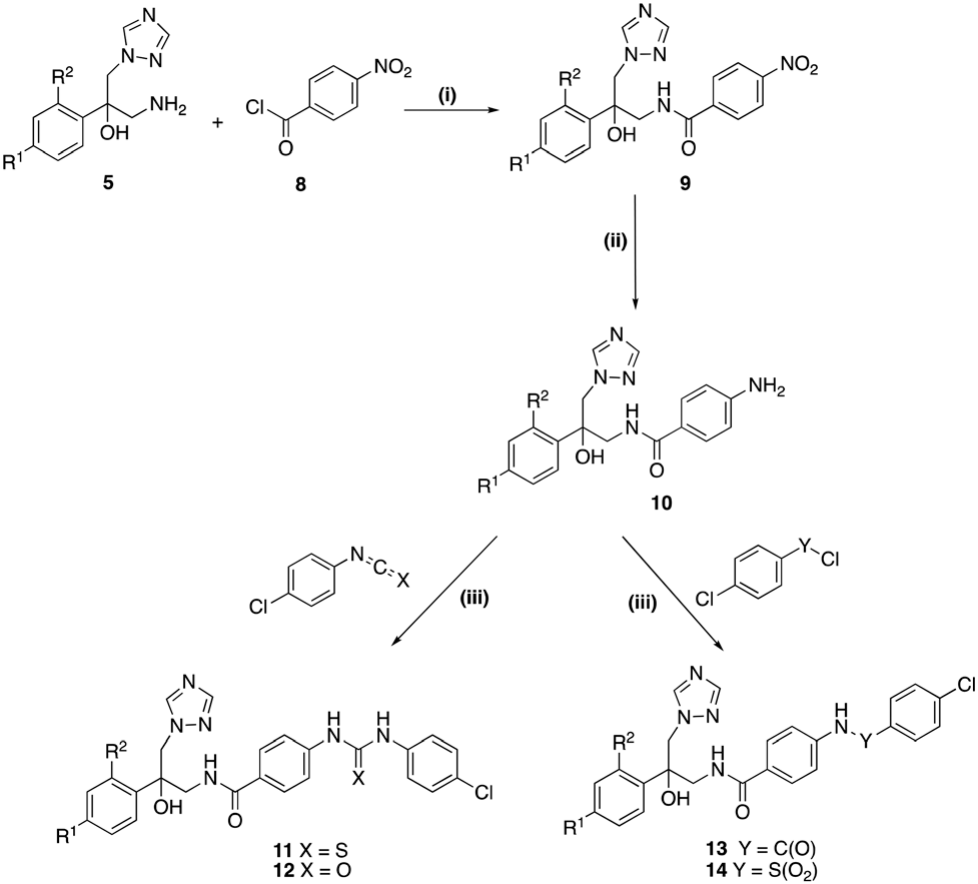
Reagents and conditions: (i) sat. aq. NaHCO_3_, CH_2_Cl_2_, r.t. o/n, 55–74% (ii) H_2_, Pd/C, EtOH, r.t., 3 h, 71–100% (iii) pyridine, r.t., o/n, 52–83% [a R^1^ = Cl, R^2^ = H; b R^1^ = Cl, R^2^ = Cl].

The nitro intermediates (9) for the preparation of the extended derivative, were prepared by reaction of 4-nitrobenzoyl chloride (8) with the free amines (5) at room temperature overnight, (Scheme 2). The nitro derivatives (9) were reduced to the amines (10) using 10% palladium on carbon in dry MeOH and H_2_ balloon for 3 h at room temperature. The synthesis of the final compounds (11–14) with the different linkers was achieved by adding 4-chlorophenyl derivatives to 4-amino-*N*-(2-(substituted phenyl)-2-hydroxy-3-(1*H*-1,2,4-triazol-1-yl)propyl)benzamide (12) in dry pyridine, and the reaction was left at room temperature overnight (Scheme 2). The extended compounds were obtained in good yields as white solids (Table 1), although lower yields were obtained for compounds 11b and 12b owing to more difficult purifications. Purity was determined either by elemental analysis or HPLC, while structure characterisation was performed by ^1^H/^13^C NMR.

### *In vitro* biological assays

#### *C. albicans* susceptibility testing

Each novel compound was screened against *C. albicans* wild-type clinical isolate CA14 and *C. albicans* laboratory strain SC5314 using the standardised CLSI M27-S4 broth dilution method.^28^ As shown in Table 2, the dichloro mid-sized compounds (6b and 7b, MIC < 0.03 μg mL^−1^) displayed promising antifungal activity against both strains compared with monochloro derivatives (6a and 7a) as well as with the control FLZ. This might be explained by the clog *P* data as the more lipophilic compounds should more readily penetrate the fungal membrane resulting in improved uptake or may relate to optimal fill of the small hydrophobic pocket leading to improved binding.

**Table 2:** MIC, IC_50_, *K*_d_ and clog *P* values for mid-sized and extended compounds

Cmpd	MIC (μg mL^−1^)		IC_50_ (μM)	*K*_d_ (nM)	clog *P*^a^
	CA14	SC5314			
6a	0.25	0.25	1.30	1445 ± 266	1.85
6b	<0.03	0.06	0.49	229 ± 45	2.46
7a	0.25	0.25	0.77	635 ± 181	1.95
7b	<0.03	<0.03	0.41	106 ± 15	2.55
11a	4	4	—	—	3.13
11b	0.25	0.5	0.67	125 ± 41.0	3.73
12a	1	2	—	—	3.86
12b	0.125	0.125	0.47	137 ± 14	4.46
13a	<0.03	<0.03	0.56	46 ± 8	3.54
13b	<0.03	<0.03	0.48	166 ± 39	4.15
14a	1	1	—	—	3.45
14b	0.25	0.25	0.78	94 ± 24	4.01
FLZ	0.125	0.125	0.31	31 ± 8	0.86

a clog *P* by Crippen's fragmentation.^29^

The dichloro with the thiourea, urea and sulfonamide linker of the extended derivatives (11b, 12b and 14b) were more effective at inhibiting *C. albicans* growth, for example: the dichloro thiourea 11b showed MIC 0.25 μg mL^−1^ compared with the monochloro 11a MIC 4 μg mL^−1^. Even though the clog *P* of the amide inhibitors (13a, clog *P* 3.53 and 13b, clog *P* 4.15) was more lipophilic than thiourea and sulfonamide (11a, clog *P* 3.13, 11b, clog *P* 3.73, 15a, clog *P* 3.45 and 15b, clog *P* 4.01) and less than urea (12a, clog *P* 3.86 and 12b, clog *P* 4.46), the mono and dichloro derivatives of the amide linker (13a and 13b) were the most effective over the three linkers as well as the standard FLZ with MIC <0.03 μg mL^−1^ (Table 2). This could be owing to the higher polarity of the thiourea and urea functional group compared with the amide, which may lead to a reduced uptake across the lipophilic fungal membrane, although other factors such as efflux and binding affinity may also play a role.

#### CaCYP51 IC_50_

Derivatives with a MIC <1 μg mL^−1^ were chosen for IC_50_ testing. In the mid-sized series (6 and 7) the dichloro derivatives of the acetyl 6b and the thiazole 7b showed inhibitory activity with IC_50_ values of 0.49 and 0.41 μM respectively compared with FLZ (IC_50_ 0.31 μM) (Table 2). The extended derivatives of the amide linker (13) all showed very good inhibition of CaCYP51 (IC_50_ 0.56 μM for 13a and 0.48 μM for 13b) (Table 2). Of all derivatives, compounds 6b, 7b, 12b, 13a and 13b had MIC and IC_50_ values comparable with or better than FLZ indicating that these compounds are promising candidates for further study.

#### Binding affinity (*K*_d_)

The novel azole compounds (6, 7, 11–14) were designed as type II binding inhibitors *i.e.* bind directly to the Fe^3+^ of the CaCYP51 haem prosthetic group by replacing the sixth axial water molecule *via* coordination with a triazole nitrogen atom.^30^

**Fig. 3:**
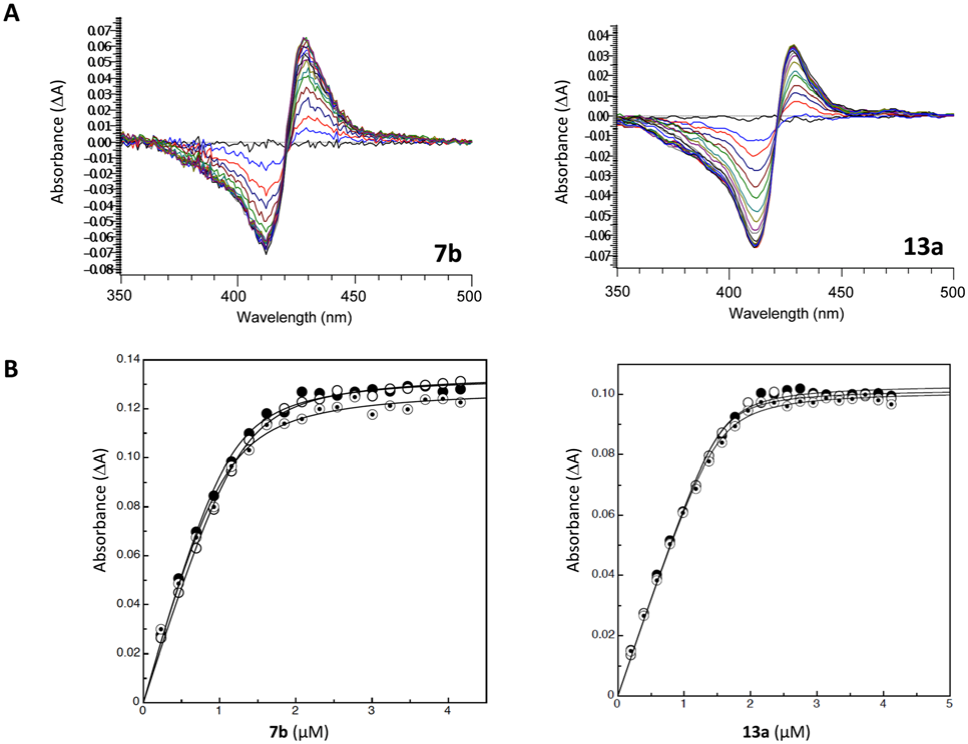
(A) Absorbance difference spectra of exemplars 7b and 13a were measured during the titration of 2.5 μM native CaCYP51 with 7b and 13a (type II binding). (B) Saturation curve of 7b and 13a derived from type II difference spectra in (A) with 2.5 μM native CaCYP51. Azole titration was performed in triplicate.

The binding affinity (*K*_d_) values for FLZ and the novel inhibitors were determined using saturation curves for CaCYP51 that measured the change in the absorbance (Δ*A* peak-trough) plotted against antifungal concentration (Fig. 3, S1 and S2†). For the mid-sized series, specifically the dichloro derivatives, the binding affinity for the thiazole (7b) compound was acceptable (*K*_d_ = 106 ± 15 nM) compared with fluconazole (*K*_d_ = 31 ± 8 nM), which showed tight binding with the haem iron. The binding affinity for the chloro and dichloro of the acetyl derivatives (6a–b) as well as the chloro of the thiazole (7a) derivative indicated weaker binding affinities (Table 2). For the extended series, the monochloro of the amide derivative (13a) showed the tightest binding (*K*_d_ = 46 ± 8 nM) with CaCYP51 haem Fe^3+^ compared with the mid-sized series as well as FLZ. Good binding affinity for the dichloro derivative of the thiourea (11b), urea (12b) and sulfonamide (14b) was observed (11b, *K*_d_ = 125 ± 41 nM, 12b, *K*_d_ = 137 ± 14 nM and 14b, *K*_d_ = 94 ± 24 nM) (Table 2).

#### *S. cerevisiae* target and resistant mutant models

The most promising thiazole derivative 7b, and the extended amide derivatives 13a and 13b were evaluated against a panel of recombinant *S. cerevisiae* strains^24,31,32^ expressing control levels of wild-type CYP51 (ScCYP51), overexpressed wild-type ScCYP51, overexpressed azole-resistant ScCYP51 Y140F/H mutations (*S. cerevisiae* Y140 equivalent to *C. albicans* Y132), overexpressed *C. albicans* efflux pumps MDR1a or CDR1B and overexpressed *H. sapiens* wild-type CYP51 (HsCYP51) (Table 3) at 24 and 48 h with each assay performed in triplicate.

**Table 3:** Susceptibility of *S. cerevisiae* strains expressing recombinant proteins to lead compounds 7b, 13a and 13b

Compound	7b	13a	13b	FLZ	PCZ
Strain	MIC (μg mL^−1^)				
*S. cerevisiae* models					
Y2411 parent	0.125	0.25	<0.03–0.06	4	<0.03
Y2300 ScCYP51	0.5–1.0	1–2	0.25	16	0.06
Y2301 Y140F	4	1–2	0.25	32	0.06
Y2513 Y140H	2–4	1	0.125	32	<0.03
*C. albicans* models					
Y525 CaMDR1a	2	0.125	<0.03–0.06	32	<0.03
Y570 CaCDR1B	>16	>16	>16	>128	8
*H. sapiens* model					
Y2760 HsCYP51	>16	>16	4–8	>128	8

The most promising thiazole derivative 7b, and the extended amide derives 13a and 13b were all active against the susceptible strain Y2411 and *S. cerevisiae* CYP51 overexpressing strain Y2300. The extended amide derivatives (13a/b) retained good activity against the azole-resistant strains Y2301 and Y2513, expressing ScCYP51 Y140F and Y140H respectively and with 13b the most effective. Against the *C. albicans* efflux pump models, 13a and 13b retained activity against strain Y525, which overexpresses the *C. albicans* MFS transporter MDR1a. However inhibitory activity was significantly reduced against strain Y570, which overexpresses the *C. albicans* ABC transporter CDR1B, consistent with both FLZ and PCZ. Good selectivity was observed for ScCYP51 compared with HsCYP51 (Y2760 strain). All three derivatives were more effective than FLZ and 13b displayed a profile comparable with PCZ (Table 3) at both 24 and 48 h. Micafungin, a β-(1–3)-d-glucan synthase inhibitor, served as a negative control with MIC values of 0.125 μg mL^−1^ for all strains.

### Computational studies

Protein–ligand complexes were prepared through docking of the *R*- and *S*-enantiomers of ligands 6a/b, 7a/b, 11a/b, 12a/b, 13a/b and 14a/b using Molecular Operating Environment (MOE) software^33^ and the crystal structure of CaCYP51 co-crystallised with PCZ (pdb 5FSA).^34^ The protein–ligand complexes were subjected to 200 ns molecular dynamics simulations using the Desmond programme of Schrödinger.^35,36^ All complexes equilibrated, although fluctuations were observed in RMSD for some compounds due to changes in binding interactions over the 200 ns MD simulations (Fig. S3 and S4†). The ligands should be positioned with the triazole above the haem Fe^3+^ and perpendicular to the haem with a distance <3 Å for optimal binding. For nine of the ligands (6a, 6b, 7b, 11a, 12a, 12b, 13a, 13b and 14b) only the *S*-enantiomer was positioned optimally, for 11b only the *R*-enantiomer was optimally positioned and for 7a and 14a both enantiomers were optimally positioned for binding with the haem (Fig. S5†).

**Fig. 4:**
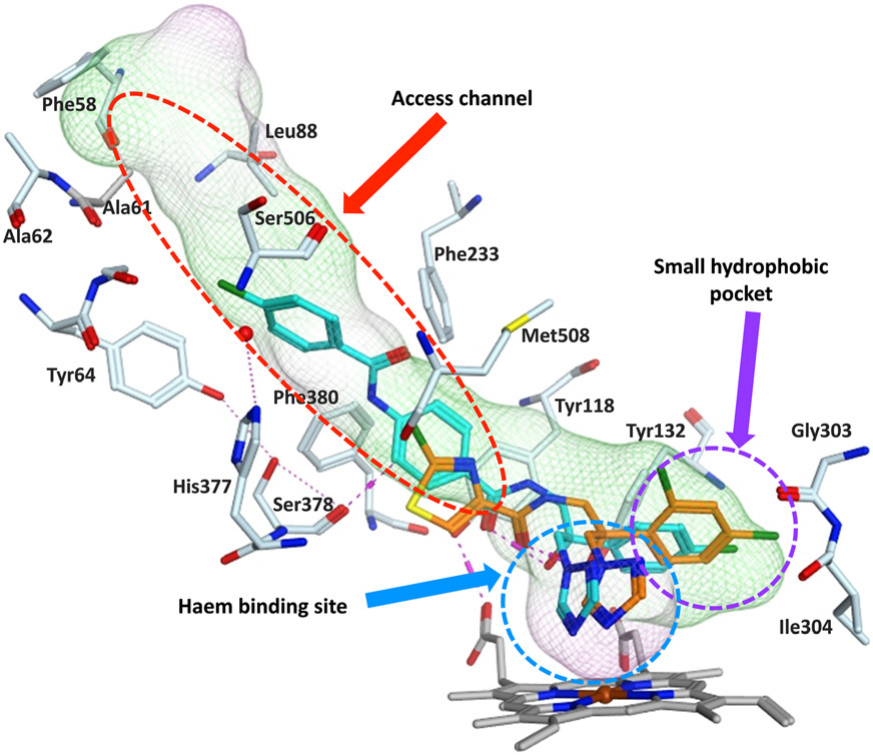
Positioning of exemplar compounds ***S*-**7b (orange) and ***S*-**13a (cyan) in the CaCYP51 protein (for clarity not all amino acids are shown).

The ligands were positioned in contact with the haem, and within the small hydrophobic pocket and the access channel (Fig. 4). The small hydrophobic pocket accommodates the chlorobenzene (*e.g.****S*-**7b) or dichlorobenzene (*e.g.****S*-**13a) ring and the haem accommodates the triazole and amide linker (Fig. 4). The medium extended compounds (6 and 7) form π–π stacking interactions with Tyr118, Tyr132 and Phe380 and H-bond interactions with Tyr132, Ser378 and Met508. The long extensions of compounds 11–14 form additional binding interactions along the access channel including Tyr64, Leu121, Phe233, Thr229, Ser506 and Ser507 (ligand interactions for each compound is provided in detail in Fig. S5 and S6†).

## Conclusions

In general, the dichlorobenzene derivatives (b) displayed better inhibitory activity (MIC, IC_50_) and binding affinity (*K*_d_) compared with the monochlorobenzene derivatives (a). Among the medium arm extensions, the benzene acetyl derivative (6b) and the thiazole derivative (7b) had MIC and IC_50_ values comparable with FLZ (Table 2), however 7b had improved binding affinity (*K*_d_ = 106 ± 15 nM) (Table 2). Comparison of the extended arm derivatives demonstrated the optimal linker with respect to antifungal activity was the amide (13), then urea (12), thiourea (11) and lastly sulfonamide (14), which was the least effective. The amide linker compounds (13a and 13b) were highly active against *C. albicans* strains CA14 and SC5314 with MIC <0.03 μg mL^−1^ and gave potent IC_50_ values (13a IC_50_ = 0.56 μM, 13b IC_50_ = 0.48 μM), but only compound 13a showed very tight binding to CaCYP51 (13a*K*_d_ = 46 ± 8 nM) while a good binding affinity was noted for 13b (*K*_d_ = 166 ± 39 nM,) compared with fluconazole (MIC = 0.125 μg mL^−1^, IC_50_ = 0.31 μM and *K*_d_ = 31 ± 8 nM) (Table 2).

Evaluation (MIC) against *S. cerevisiae* models indicated the amide of the three lead compounds 7b, 13a and 13b all outperformed FLZ, with 13b optimal as it retained activity against mutant ScCYP51 Y140F/H mutant strains and the strain overexpressing *C. albicans* efflux pump MDR1a with a similar profile to PCZ. Computational studies suggest the *S*-enantiomers are generally preferred for optimal binding and positioning within the CaCYP51 active site, with the extended arm derivatives forming additional binding interactions in the access channel (Fig. 4 and S5†).

**Fig. 5:**
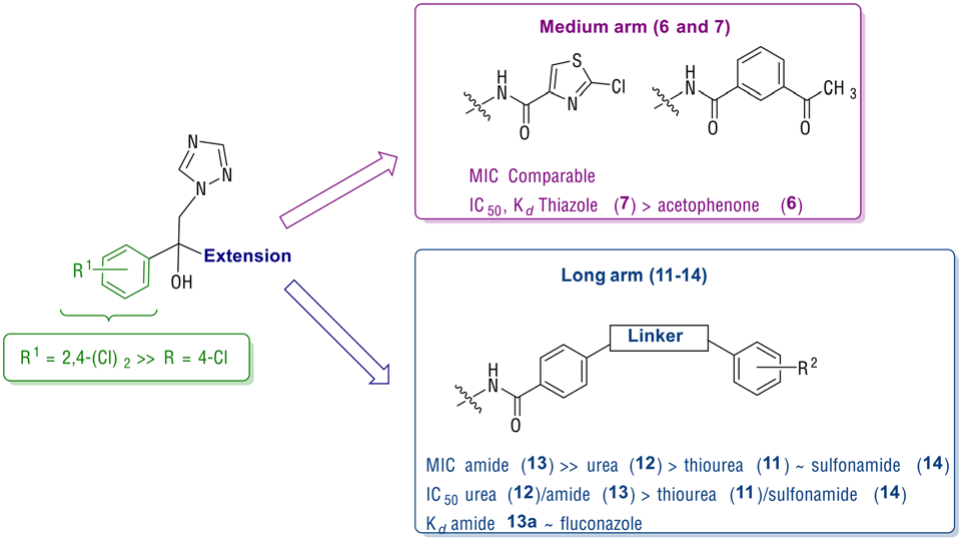
Summary of antifungal (MIC), enzyme inhibition (IC_50_) and binding affinity (*K*_d_) of the medium (6 and 7) and long arm (11–14) extended compounds.

In summary (Fig. 5), the dichlorobenzene ring was optimal for binding within the small hydrophobic pocket and, in combination with an extended arm (*e.g.* as seen in 13b) performed better in resistant strains than FLZ owing to additional binding opportunities along the access channel to stabilise ligand–protein interactions.

Consideration of the physicochemical properties of the most promising medium (7b) and extended (13a/b) arm derivatives compared with reference antifungal agents (Table 4), showed that the thiazole derivative 7b is comparable with FLZ, while the extended derivatives (13a/b) are intermediate between FLZ and PCZ. Compounds 13a and 13b are just over the ideal molecular weight resulting in one violation of Lipinski's Ro5, however this is still a significant improvement compared with PCZ with three violations. Improved physicochemical properties would make these compounds more suitable for oral administration, a major limitation of ITZ and PCZ, which combined with the addition of linkers and long arms capable of H-bonding interactions enables antifungal activity against resistant strains, in particular common mutations in the CYP51 active site.

**Table 4:** Physicochemical properties of promising compounds and clinical antifungal agents

Cmpd	MW	clog *P*	*n*_ON_	*n*_OHNH_	*n*_rotb_	*n*_viol_
7b	399.81	2.55	7	2	6	0
13a	510.38	3.54	8	3	8	1
13b	544.83	4.15	8	3	8	1
FLZ	306.27	0.86	7	1	5	0
PCZ	700.77	5.74	12	1	12	3

## Experimental

### General

All chemicals, reagents and solvents were purchased from Sigma-Aldrich, Alfa Aesar, VWR, Acros and Fluka. Solvents were dried over molecular sieves (4 Å) prior to use. For column chromatography, a glass column was slurry packed in the appropriate eluent with silica gel (Fluka Kieselgel 60). TLC was performed on pre-coated silica plates (ALUGRAM® SIL G/UV254) with visualisation *via* UV light (254 nm). Melting points were determined using an electrothermal instrument (Gallenkamp melting point apparatus) and were uncorrected. ^1^H and ^13^C NMR spectra were recorded on a Bruker Advance DP500 spectrometer operating at 500 MHz and 125 MHz, respectively. Chemical shifts are given in parts per million (ppm) relative to the internal standard tetramethylsilane (Me_4_Si). HPLC-HRMS or HPLC was performed at the Department of Pharmacy and Pharmacology, University of Bath, Bath, UK (method A) or at Cardiff University (method B). Method A: was performed on a Zorbax Eclipse Plus C18 Rapid Resolution 2.1 × 50 mm, 1.8 μm particle size using a 7.5 minute gradient method 5 : 95 v/v water : methanol with 0.1% formic acid as additive; method B: was performed on a Shimadzu LC-2030C Plus C18 Rapid Resolution 250 × 4.6 mm, 5 μm particle size using a 7–10 min gradient method 5 : 95 v/v water: methanol. Details for the preparation of the intermediate amines (5 and 12) are provided in the ESI.†

### Chemistry

#### General method for the synthesis of azoles (6, 7, 11–14)

To a solution of 3-acetylbenzoic acid or 2-chlorothiazole-4-carboxylic acid (1.5 eq.) in dry DMF (5 mL) was added CDI (1.5 eq.) and the reaction stirred at room temperature for 1 h. Then, a solution of 1-amino-2-(4-chlorophenyl)-3-(1*H*-1,2,4-triazol-1-yl)propan-2-ol (5) (1 eq.) in dry DMF (5 mL) was added and the reaction stirred at room temperature overnight. The reaction mixture was quenched with ice/cooled H_2_O (25 mL), the residue extracted with EtOAc (50 mL), washed with sat. aq. NaCl (25 mL × 2), and dried (MgSO_4_). The organic layer was evaporated under reduced pressure to give a light-yellow oil. Gradient column chromatography was used to purify the compound, which eluted with CH_2_Cl_2_–MeOH to give a semi solid or white solid on drying (6 and 7). To prepare 11–14: to a cooled (0 °C, ice bath) solution of 4-amino-*N*-(2-(4-chlorophenyl)-2-hydroxy-3-(1*H*-1,2,4-triazol-1-yl)propyl)benzamide (10) (1 eq.) in dry pyridine (5 mL) was added 4-chlorophenyl isothiocyanate or 4-chlorophenyl isocyanate or 4-chlorobenzoyl chloride or 4-chlorobenzenesulfonyl chloride (1.5 eq.) in portions and the reaction stirred at room temperature overnight. The solvent was evaporated, and the resulting oil extracted with EtOAc (50 mL), washed with 1 M aq. HCl (25 mL), H_2_O (2 × 25 mL), and dried (MgSO_4_). The organic layer was evaporated under reduced pressure to give a crude yellow-orange oil. The desired product was purified by gradient column chromatography using CH_2_Cl_2_–MeOH.

##### 3-Acetyl-*N*-(2-(4-chlorophenyl)-2-hydroxy-3-(1*H*-1,2,4-triazol-1-yl)propyl)benzamide (6a, R^1^ = Cl, R^2^ = H)

Prepared from 1-amino-2-(4-chlorophenyl)-3-(1*H*-1,2,4-triazol-1-yl)propan-2-ol (5a) (0.15 g, 0.59 mmol) and 3-acetyl benzoic acid (0.14 g, 0.88 mmol). Purified by gradient column chromatography eluting with 3.5% MeOH in CH_2_Cl_2_. Product obtained as a semisolid, yield 0.12 g (52%). TLC (CH_2_Cl_2_–MeOH 9.5 : 0.5 v/v), *R*_f_ 0.4. ^1^H NMR (DMSO-d_6_): *δ* 8.55 (t, *J* = 5.7 Hz, 1H, NH), 8.27 (d, *J* = 7.2 Hz, 1H, Ar), 8.26 (s, 1H, triaz), 8.09 (d, *J* = 7.8 Hz, 1H, Ar), 7.98 (d, *J* = 8.3 Hz, 1H, Ar), 7.83 (s, 1H, triaz), 7.60 (d, *J* = 7.8 Hz, 1H, Ar), 7.45 (d, *J* = 8.8 Hz, 2H, Ar), 7.32 (d, *J* = 8.8 Hz, 2H, Ar), 6.07 (s, 1H, OH, ex), 4.61 (q, *J* = 14.4 Hz, 2H, CH̲_2̲_-triaz), 3.90 (dd, *J* = 6.8, 14.0 Hz, 1H, CH̲_2_–NH), 3.65 (dd, *J* = 5.3, 14.0 Hz, 1H, CH̲_2_–NH), 2.61 (s, 3H, CH̲_3̲_). ^13^C NMR (DMSO-d_6_): *δ* 197.99 (C, CH_3_C=O), 167.08 (C, C=O, amide), 150.01 (CH, triaz), 145.45 (CH, triaz), 141.40 (C, Ar), 137.19 (C, Ar), 134.92 (C, Ar), 132.31 (CH, Ar), 132.17 (C, C–Cl), 131.41 (CH, Ar), 129.29 (CH, Ar), 128.37 (2× CH, Ar), 128.09 (2× CH, Ar), 127.29 (2× CH, Ar), 76.28 (C̲–OH), 57.01 (C̲H_2_-triaz), 48.24 (C̲H_2_–NH), 27.30 (C̲H_3_). HPLC (method A): 100%, RT = 4.37 min. HRMS (ESI, *m*/*z*). Calcd for C_20_H_19_ClN_4_O_3_ [M + H]^+^, 399.1224; found, 399.1225.

##### 3-Acetyl-*N*-(2-(2,4-dichlorophenyl)-2-hydroxy-3-(1*H*-1,2,4-triazol-1yl)propyl)benzamide (6b, R^1^ = Cl, R^2^ = Cl)

Prepared from 1-amino-2-(2,4-dichlorophenyl)-3-(1*H*-1,2,4-triazol-1-yl)propan-2-ol (5b) (0.15 g, 0.52 mmol) and 3-acetyl benzoic acid (0.13 g, 0.78 mmol). Purified by gradient column chromatography eluting with 3.5% MeOH in CH_2_Cl_2_. Product obtained as a white solid, yield 0.1 g (45%). M.p.: 160–162 °C. TLC (CH_2_Cl_2_–MeOH 9.5 : 0.5 v/v), *R*_f_ = 0.45. ^1^H NMR (DMSO-d_6_): *δ* 8.72 (t, *J* = 6.0 Hz, 1H, NH), 8.34 (s, 1H, triaz), 8.30 (s, 1H, Ar), 8.10 (d, *J* = 7.8 Hz, 1H, Ar), 8.01 (d, *J* = 8.3 Hz, 1H, Ar), 7.74 (s, 1H, triaz), 7.59 (m, 3H, Ar), 7.29 (dd, *J* = 2.2, 8.6 Hz, 1H, Ar), 6.35 (s, 1H, OH, ex), 5.10 (d, *J* = 14.4 Hz, 1H, CH̲_2̲_-triaz), 4.70 (d, *J* = 14.4 Hz, 1H, CH̲_2̲_-triaz), 4.08 (dd, *J* = 5.7, 14.0 Hz, 1H, CH̲_2̲_-NH), 3.97 (dd, *J* = 6.4, 14.0 Hz, 1H, CH̲_2̲_–NH), 2.61 (s, 3H, CH̲_3̲_). ^13^C NMR (DMSO-d_6_): *δ* 198.00 (C, CH_3_C=O), 167.58 (C, C=O, amide), 151.02 (CH, triaz), 145.56 (CH, triaz), 138.11 (C, Ar), 137.20 (C, Ar), 134.71 (C, Ar), 133.29 (C, C–Cl), 132.40 (CH, Ar), 131.51 (CH, Ar), 131.50 (C, C–Cl), 130.43 (CH, Ar), 129.30 (2× CH, Ar), 127.39 (2× CH, Ar), 76.90 (C̲–OH), 54.09 (C̲H_2_-triaz), 45.95 (C̲H_2_–NH), 27.32 (C̲H_3_). HPLC (method A): 100%, RT = 4.49 min. HRMS (ESI, *m*/*z*), calcd for C_20_H_18_Cl_2_N_4_O_3_ [M + H]^+^, 433.0834; found, 433.0832.

##### 2-Chloro-*N*-(2-(4-chlorophenyl)-2-hydroxy-3-(1*H*-1,2,4-triazol-1-yl)propyl)thiazole-4-carboxamide (7a, R^1^ = Cl, R^2^ = H)

Prepared from 1-amino-2-(2,4-dichlorophenyl)-3-(1*H*-1,2,4-triazol-1-yl)propan-2-ol (5a) (0.16 g, 0.63 mmol) and 2-chlorothiazole-4-carboxylic acid (0.15 g, 0.95 mmol). Purified by gradient column chromatography eluting with 3.5% MeOH in CH_2_Cl_2_. Product obtained as a semisolid, yield 0.17 g (68%). TLC (CH_2_Cl_2_–MeOH 9.5 : 0.5 v/v), *R*_f_ = 0.45. ^1^H NMR (DMSO-d_6_): *δ* 8.23 (s, 1H, thiazole), 8.22 (s, 1H, triaz), 8.10 (t, *J* = 5.2 Hz, 1H, NH), 7.84 (s, 1H, triaz), 7.41 (d, *J* = 8.8 Hz, 2H, Ar), 7.32 (d, *J* = 8.8 Hz, 2H, Ar), 6.12 (s, 1H, OH, ex), 4.53 (s, 2H, CH̲_2̲_-triaz), 3.96 (dd, *J* = 7.3, 14.1 Hz, 1H, CH̲_2̲_–NH), 3.58 (dd, *J* = 5.1, 14.1 Hz, 1H, CH̲_2̲_–NH). ^13^C NMR (DMSO-d_6_): *δ* 160.06 (C, C=O, amide), 151.44 (C, C–Cl), 151.13 (CH, triaz), 147.72 (C, Ar), 145.45 (CH, triaz), 141.28 (C, thiazole), 132.28 (C, Ar), 128.30 (2× CH, Ar), 128.19 (2× CH, Ar), 128.03 (CH, thiazole), 75.72 (C̲–OH), 57.26 (C̲H_2_-triaz), 47.22 (C̲H_2_-NH). HPLC (method A): 100%, RT = 4.52 min. HRMS (ESI, *m*/*z*). Calcd for C_15_H_13_Cl_2_N_5_O_2_S [M + H]^+^, 398.0245; found, 398.0242.

##### 2-Chloro-*N*-(2-(2,4-dichlorophenyl)-2-hydroxy-3-(1*H*-1,2,4-triazol-1-yl)propyl)thiazole-4-carboxamide (7b, R^1^ = Cl, R^2^ = Cl)

Prepared from 1-amino-2-(2,4-dichlorophenyl)-3-(1*H*-1,2,4-triazol-1-yl)propan-2-ol (5b) (0.15 g, 0.52 mmol) and 2-chlorothiazole-4-carboxylic acid (0.13 g, 0.78 mmol). Purified by gradient column chromatography eluting with 2.5% MeOH in CH_2_Cl_2._ Product obtained as a pale yellow wax, yield 0.15 g (68%). TLC (CH_2_Cl_2_–MeOH 9.5 : 0.5 v/v), *R*_f_ = 0.55. ^1^H NMR (DMSO-d_6_): *δ* 8.32 (s, 1H, thiazole), 8.26 (t, *J* = 5.9 Hz, 1H, NH), 8.25 (s, 1H, triaz), 7.72 (s, 1H, triaz), 7.54 (d, *J* = 7.7 Hz, 2H, Ar), 7.29 (dd, *J* = 2.2, 8.6 Hz, 1H, Ar), 6.33 (s, 1H, OH, ex), 5.03 (d, *J* = 14.4 Hz, 1H, CH̲_2̲_-triaz), 4.64 (d, *J* = 14.4 Hz, 1H, CH̲_2̲_-triaz), 4.05 (dd, *J* = 6.7, 14.0 Hz, 1H, CH̲_2̲_–NH), 3.99 (dd, *J* = 5.8, 14.0 Hz, 1H, CH̲_2̲_–NH). ^13^C NMR (DMSO-d_6_): *δ* 160.43 (C, C=O, amide), 151.45 (C, C–Cl), 151.03 (CH, triaz), 147.67 (C, Ar), 145.58 (CH, triaz), 137.98 (C, thiazole), 133.35 (C, C–Cl), 132.07 (C, C–Cl), 131.49 (CH, Ar), 130.47 (CH, Ar), 128.18 (CH, thiazole), 127.30 (CH, Ar), 76.27 (C̲–OH), 54.14 (C̲H_2_-triaz), 45.06 (C̲H_2_–NH). HPLC (method A): 100%, RT = 4.63 min. HRMS (ESI, *m*/*z*). Calcd for C_15_H_12_Cl_3_N_5_O_2_S [M + H]^+^, 431.9855; found, 433.9826.

##### *N*-(2-(4-Chlorophenyl)-2-hydroxy-3-(1*H*-1,2,4-triazol-1-yl)propyl)-4-(3-(4-chlorophenyl)thioureido)benzamide (11a, R^1^ = Cl, R^2^ = H, X = S)

Prepared from 4-amino-*N*-(2-(4-chlorophenyl)-2-hydroxy-3-(1*H*-1,2,4-triazol-1-yl)propyl)benzamide (10a) (0.16 g, 043 mmol) and 4-chlorophenyl isothiocyanate (0.11 g, 0.64 mmol). Purified by gradient column chromatography eluting with 2.5% MeOH in CH_2_Cl_2._ Product obtained as a white solid, yield 0.15 g (68%). M.p.: 146–148 °C. TLC (CH_2_Cl_2_–MeOH 9.5 : 0.5 v/v), *R*_f_ = 0.23. ^1^H NMR (DMSO-d_6_): *δ* 10.05 (s, 1H, NH–thiourea), 10.00 (s, 1H, NH–thiourea), 8.22 (t, *J* = 5.9 Hz, 1H, NH–CH_2_), 8.20 (s, 1H, triaz), 7.84 (s, 1H, triaz), 7.70 (d, *J* = 8.9 Hz, 2H, Ar), 7.55 (dd, *J* = 8.8, 21.2 Hz, 4H, Ar), 7.43 (d, *J* = 7.1 Hz, 2H, Ar), 7.39 (d, *J* = 9.0 Hz, 2H, Ar), 7.29 (m, 2H, Ar), 6.05 (s, 1H, OH, ex), 5.74 (dd, *J* = 14.3, 20.3 Hz, 2H, CH̲_2̲_-triaz), 3.86 (dd, *J* = 6.7, 14.0 Hz, 1H, CH̲_2̲_–NH), 3.63 (dd, *J* = 5.2, 14.1 Hz, 1H, CH̲_2̲_–NH). ^13^C NMR (DMSO-d_6_): *δ* 179.97 (C, C=S), 167.33 (C, C=O), 150.96 (CH, triaz), 145.38 (CH, triaz), 142.70 (C, Ar), 142.45 (C, Ar), 141.48 (C, Ar), 138.75 (C, C–Cl), 132.14 (C, C–Cl), 129.85 (C, Ar), 129.84 (2× CH, Ar), 128.23 (2× CH, Ar), 128.17 (2× CH, Ar), 126.24 (2× CH, Ar), 125.74 (2× CH, Ar), 122.74 (2× CH, Ar), 76.48 (C, C̲–OH), 57.40 (C̲H_2_-triaz), 48.35 (C̲H_2_-NH). HPLC (method B): 99%, RT = 4.78 min. HRMS (ESI, *m*/*z*). Calcd for C_25_H_22_Cl_2_N_6_O_2_S [M + H]^+^, 541.0980; found, 541.0971.

##### 4-(3-(4-Chlorophenyl)thioureido)-*N*-(2-(2,4-dichlorophenyl)-2-hydroxy-3-(1*H*-1,2,4-triazol-1-yl)propyl)benzamide (11b, R^1^ = Cl, R^2^ = Cl, X = S)

Prepared from 4-amino-*N*-(2-(2,4-dichlorophenyl)-2-hydroxy-3-(1*H*-1,2,4-triazol-1-yl)propyl) benzamide (10b) (0.17 g, 0.41 mmol) and 4-chlorophenyl isothiocyanate (0.1 g, 0.62 mmol). Purified by gradient column chromatography eluting with 3.5% MeOH in CH_2_Cl_2._ Product obtained as a white solid, yield 0.13 g (56%). M.p.: 138–140 °C. TLC (CH_2_Cl_2_–MeOH 9.5 : 0.5 v/v), *R*_f_ = 0.32. ^1^H NMR (DMSO-d_6_): *δ* 10.06 (s, 1H, NH–thiourea), 10.03 (s, 1H, NH–thiourea), 8.47 (t, *J* = 5.9 Hz, 1H, NH-CH_2_), 8.34 (s, 1H, triaz), 7.74 (s, 1H, triaz), 7.73 (d, *J* = 8.8 Hz, 2H, Ar), 7.57 (m, 4H, Ar), 7.51 (d, *J* = 8.9 Hz, 2H, Ar), 7.39 (d, *J* = 8.9 Hz, 2H, Ar), 7.29 (dd, *J* = 2.3, 8.7 Hz, 1H, Ar), 6.46 (s, 1H, OH-ex), 5.05 (d, *J* = 14.4 Hz, 1H, CH̲_2̲_-triaz), 4.68 (d, *J* = 14.4 Hz, 1H, CH̲_2̲_-triaz), 4.02 (dd, *J* = 5.7, 14.2 Hz, 1H, CH̲_2̲_–NH), 3.97 (dd, *J* = 6.5, 14.2 Hz, 1H, CH̲_2̲_-NH). ^13^C NMR (DMSO-d_6_): *δ* 179.90 (C, C=S), 168.02 (C, C=O), 151.01 (CH, triaz), 145.59 (CH, triaz), 142.89 (C, Ar), 138.76 (C, Ar), 138.19 (C, C–Cl), 133.26 (C, C–Cl), 132.17 (C, C–Cl), 132.04 (C, C–Cl), 131.54 (CH, Ar), 130.41 (CH, Ar), 128.81 (2× CH, Ar), 128.32 (2× CH, Ar), 127.27 (CH, Ar), 125.72 (2× CH, Ar), 122.70 (2× CH, Ar), 76.99 (C, C̲–OH), 54.18 (C̲H_2_-triaz), 46.05 (C̲H_2_–NH). Anal. calcd for C_25_H_21_Cl_3_N_6_O_2_S (575.89): C 52.13%, H 3.68%, N 14.59%. Found: C 52.29%, H 3.82%, N 14.71%.

##### *N*-(2-(4-Chlorophenyl)-2-hydroxy-3-(1*H*-1,2,4-triazol-1-yl)propyl)-4-(3-(4-chlorophenyl)ureido)benzamide (12a, R^1^ = Cl, R^2^ = H, X = O)

Prepared from 4-amino-*N*-(2-(4-chlorophenyl)-2-hydroxy-3-(1*H*-1,2,4-triazol-1-yl)propyl)benzamide (10a) (0.16 g, 0.43 mmol) and 4-chlorophenyl isocyanate (0.1 g, 0.64 mmol). Purified by gradient column chromatography eluting with 6% MeOH in CH_2_Cl_2_. Product obtained as a white solid, yield 0.14 g (63%). M.p.: 226–228 °C. TLC (CH_2_Cl_2_–MeOH 9.5 : 0.5 v/v), *R*_f_ = 0.35. ^1^H NMR (DMSO-d_6_): *δ* 8.99 (s, 1H, NH–urea), 8.90 (s, 1H, NH–urea), 8.25 (s, 1H, triaz), 8.22 (t, *J* = 5.7 Hz, 1H, NH–CH_2_), 7.83 (s, 1H, triaz), 7.54 (dd, *J* = 3.2, 8.9 Hz, 2H, Ar), 7.48 (m, 4H, Ar), 7.43 (d, *J* = 8.8 Hz, 2H, Ar), 7.31 (m, 4H, Ar), 6.16 (s, 1H, OH-ex), 4.60 (dd, *J* = 14.3, 21.5 Hz, 2H, CH̲_2̲_-triaz), 3.85 (dd, *J* = 6.7, 14.1 Hz, 1H, CH̲_2̲_–NH), 3.63 (dd, *J* = 5.2, 14.0 Hz, 1H, CH̲_2̲_–NH). ^13^C NMR (DMSO-d_6_): *δ* 167.51 (C, C=O), 152.63 (C, C=O), 151.00 (CH, triaz), 145.45 (CH, triaz), 143.01 (C, Ar), 142.50 (C, Ar), 141.52 (C, Ar), 138.88 (C, C–Cl), 132.12 (C, C–Cl), 129.10 (C, Ar), 128.80 (2× CH, Ar), 128.38 (2× CH, Ar), 128.09 (2× CH, Ar), 120.34 (2× CH, Ar), 117.66 (2× CH, Ar), 76.34 (C, C̲–OH), 57.14 (C̲H_2_-triaz), 48.24 (C̲H_2_–NH). Anal. calcd. for C_25_H_22_Cl_2_N_6_O_3_, (525.39): C 57.15%, H 4.22%, N 15.99%. Found: C 57.53%, H 4.28%, N 15.94%.

##### 4-(3-(4-Chlorophenyl)ureido)-*N*-(2-(2,4-dichlorophenyl)-2-hydroxy-3-(1*H*-1,2,4-triazol-1-yl)propyl)benzamide (12b, R^1^ = Cl, R^2^ = Cl, X = O)

Prepared from 4-amino-*N*-(2-(2,4-dichlorophenyl)-2-hydroxy-3-(1*H*-1,2,4-triazol-1-yl)propyl)benzamide (10b) (0.16 g, 0.43 mmol) and 4-chlorophenyl isocyanate (0.1 g, 0.64 mmol). Purified by gradient column chromatography eluting with 6% MeOH in CH_2_Cl_2._ Product obtained as a white solid, yield 0.11 g (52%). M.p.: 216–218 °C. TLC (CH_2_Cl_2_–MeOH 9.5 : 0.5 v/v), *R*_f_ = 0.37. ^1^H NMR (DMSO-d_6_): *δ* 9.06 (s, 1H, NH–urea), 8.97 (s, 1H, NH–urea), 8.42 (t, *J* = 6.0 Hz, 1H, NH–CH_2_), 8.34 (s, 1H, triaz), 7.72 (d, *J* = 11.0 Hz, 2H, Ar), 7.70 (s, 1H, triaz), 7.57 (dd, *J* = 8.6, 11.0 Hz, 2H, Ar), 7.49 (m, 4H, Ar), 7.33 (d, *J* = 8.9 Hz, 2H, Ar), 7.28 (dd, *J* = 2.2, 8.6 Hz, 1H, Ar), 6.53 (s, 1H, OH, ex), 5.05 (d, *J* = 14.3 Hz, 1H, CH̲_2̲_-triaz), 4.67 (d, *J* = 14.3 Hz, 1H, CH̲_2̲_-triaz), 4.02 (dd, *J* = 5.5, 14.1 Hz, 1H, CH̲_2̲_–NH), 3.95 (dd, *J* = 6.3, 14.1 Hz, 1H, CH̲_2̲_–NH). ^13^C NMR (DMSO-d_6_): *δ* 168.21 (C, C=O), 152.66 (C, C=O), 151.00 (CH, triaz), 145.45 (CH, triaz), 143.18 (C, Ar), 138.90 (C, Ar), 138.25 (C, C–Cl), 133.25 (C, C–Cl), 132.02 (C, C–Cl), 131.55 (CH, Ar), 131.24 (CH, Ar), 130.04 (CH, Ar), 129.10 (2× CH, Ar), 128.93 (CH, Ar), 127.27 (CH, Ar), 120.35 (2× CH, Ar), 117.64 (2× CH, Ar), 77.03 (C, C̲–OH), 54.19 (C̲H_2_-triaz), 46.12 (C̲H_2_–NH). Anal. calcd. For C_25_H_21_Cl_3_N_6_O_3_ (559.83): C 53.64%, H 3.78%, N 15.00%. Found: C 53.84%, H 3.68%, N 14.63%.

##### 4-Chloro-*N*-(4-((2-(4-chlorophenyl)-2-hydroxy-3-(1*H*-1,2,4-triazol-1-yl)propyl) carbamoyl) phenyl)benzamide (13a, R^1^ = Cl, R^2^ = H, Y = C(O))

Prepared from 4-amino-*N*-(2-(4-chlorophenyl)-2-hydroxy-3-(1*H*-1,2,4-triazol-1-yl)propyl)benzamide (10a) (0.15 g, 0.42 mmol) and 4-chlorobenzoyl chloride (0.1 g, 0.64 mmol). Purified by gradient column chromatography eluting with 6% MeOH in CH_2_Cl_2._ Product obtained as a white solid, yield 0.16 g (80%). M.p.: 222–224 °C. TLC (CH_2_Cl_2_-MeOH 9.5 : 0.5 v/v), *R*_f_ = 0.33. ^1^H NMR (DMSO-d_6_): *δ* 10.50 (s, 1H, NH-amide), 8.28 (t, *J* = 5.7 Hz, 1H, NH-CH_2_), 8.26 (s, 1H, triaz), 7.99 (d, *J* = 8.8 Hz, 2H, Ar), 7.84 (s, 1H, triaz), 7.83 (dd, *J* = 3.8, 8.9 Hz, 2H, Ar), 7.76 (d, *J* = 8.9 Hz, 2H, Ar), 7.62 (d, *J* = 8.8 Hz, 2H, Ar), 7.44 (d, *J* = 8.8 Hz, 2H, Ar), 7.30 (m, 2H, Ar), 6.14 (s, 1H, OH-ex), 4.59 (dd, *J* = 14.4, 21.9 Hz, 2H, CH̲_2̲_-triaz), 3.86 (dd, *J* = 6.8, 14.1 Hz, 1H, CH̲_2̲_–NH), 3.64 (dd, *J* = 5.3, 14.1 Hz, 1H, CH̲_2̲_-NH). ^13^C NMR (DMSO-d_6_): *δ* 167.38 (C, C=O), 165.13 (C, C=O), 151.00 (CH, triaz), 145.45 (CH, triaz), 142.26 (C, Ar), 141.49 (C, Ar), 137.10 (C, C–Cl), 133.76 (C, Ar), 132.14 (C, C–Cl), 130.17 (2× CH, Ar), 129.34 (C, Ar), 128.97 (2× CH, Ar), 128.53 (2× CH, Ar), 128.09 (2× CH, Ar), 126.25 (2× CH, Ar), 119.91 (2× CH, Ar), 76.33 (C, C̲–OH), 57.10 (C̲H_2_-triaz), 48.24 (C̲H_2_–NH). Anal. calcd. for C_25_H_21_Cl_2_N_5_O_3_ (510.38): C 58.83%, H 4.15%, N 13.72%. Found: C 58.78%, H 4.07%, N 13.68%.

##### 4-Chloro-*N*-(4-((2-(2,4-dichlorophenyl)-2-hydroxy-3-(1*H*-1,2,4-triazol-1-yl)propyl) carbamoyl)phenyl)benzamide (13b, R^1^ = Cl, R^2^ = Cl, Y = C(O))

Prepared from 4-amino-*N*-(2-(2,4-dichlorophenyl)-2-hydroxy-3-(1*H*-1,2,4-triazol-1-yl)propyl)benzamide (10b) (0.16 g, 0.39 mmol) and 4-chlorobenzoyl chloride (0.16 g, 0.6 mmol). Purified by gradient column chromatography eluting with 6% MeOH in CH_2_Cl_2._ Product obtained as a white solid, yield 0.16 g (76%). M.p.: 190–192 °C. TLC (CH_2_Cl_2_–MeOH 9.5 : 0.5 v/v), *R*_f_ = 0.32. ^1^H NMR (DMSO-d_6_): *δ* 10.51 (s, 1H, NH–amide), 8.49 (t, *J* = 5.9 Hz, 1H, NH–CH_2_), 8.35 (s, 1H, triaz), 7.99 (d, *J* = 8.6 Hz, 2H, Ar), 7.84 (d, *J* = 8.8 Hz, 2H, Ar), 7.79 (d, *J* = 8.8 Hz, 2H, Ar), 7.74 (s, 1H, triaz), 7.62 (d, *J* = 8.6 Hz, 2H, Ar), 7.57 (dd, *J* = 8.6, 13.4 Hz, 2H, Ar), 7.29 (dd, *J* = 2.2, 8.6 Hz, 1H, Ar), 6.50 (s, 1H, OH, ex), 5.07 (d, *J* = 14.3 Hz, 1H, CH̲_2̲_-triaz), 4.68 (d, *J* = 14.3 Hz, 1H, CH̲_2̲_-triaz), 4.04 (dd, *J* = 5.5, 14.0 Hz, 1H, CH̲_2̲_–NH), 3.97 (dd, *J* = 6.5, 14.0 Hz, 1H, CH̲_2̲_–NH). ^13^C NMR (DMSO-d_6_): *δ* 168.02 (C, C=O), 165.14 (C, C=O), 151.00 (CH, triaz), 145.58 (CH, triaz), 142.38 (C, Ar), 138.21 (C, Ar), 137.11 (C, C–Cl), 133.75 (C, C–Cl), 133.26 (C, C–Cl), 132.04 (C, Ar), 131.55 (CH, Ar), 130.41 (CH, Ar), 130.17 (2× CH, Ar), 129.06 (C, Ar), 128.98 (2× CH, Ar), 128.65 (2× CH, Ar), 127.27 (CH, Ar), 119.89 (2× CH, Ar), 76.99 (C, C̲–OH), 54.16 (C̲H_2_-triaz), 46.68(C̲H_2_–NH). Anal. calcd. for C_25_H_20_Cl_3_N_5_O_3_ (544.82): C 54.39%, H 3.65%, N 12.68%. Found: C 54.05%, H 3.80%, N 12.31%.

##### *N*-(2-(4-Chlorophenyl)-2-hydroxy-3-(1*H*-1,2,4-triazol-1-yl)propyl)-4-((4-chlorophenyl) sulfonamido)benzamide (14a R^1^ = Cl, R^2^ = H, Y = S(O_2_))

Prepared from 4-amino-*N*-(2-(4-chlorophenyl)-2-hydroxy-3-(1*H*-1,2,4-triazol-1-yl)propyl)benzamide (10a) (0.30 g, 0.82 mmoL) and 4-chlorobenzenesulfonyl chloride (0.26 g, 1.23 mmol). Purified by gradient column chromatography eluting with 3.5% MeOH in CH_2_Cl_2._ Product obtained as a white solid, yield 0.35 g (77%). M.p. 236–238 °C. TLC (CH_2_Cl_2_–MeOH 9.5 : 0.5 v/v), *R*_f_ = 0.45. ^1^H NMR (DMSO-d_6_): *δ* 10.73 (br.s, 1H, N*H*SO_2_), 8.23 (s, 1H, triazole), 8.21 (t, *J* = 6.0 Hz, 1H, NH), 7.81 (s, 1H, triazole), 7.79 (d, *J* = 8.8 Hz, 2H, Ar), 7.63 (m, 4H, Ar), 7.41 (d, *J* = 8.7 Hz, 2H, Ar), 7.28 (m, 2H, Ar), 7.16 (m, 2H, Ar), 6.04 (s, 1H, OH), 4.56 (dd, *J* = 14.4, 25.0 Hz, 2H, CH_2_-triazole), 3.79 (dd, *J* = 6.6, 14.0 Hz, 1H, NHC*Ha*Hb), 3.59 (dd, *J* = 5.3, 14.0 Hz, 1H, NHCHa*Hb*). ^13^C NMR (DMSO-d_6_): *δ* 167.19 (C, C=O), 151.00 (CH, triazole), 145.44 (CH, triazole), 141.45 (C, Ar), 140.85 (C, Ar), 138.65 (C, Ar), 138.48 (C, C–Cl), 132.15 (C, C–Cl), 130.03 (2× CH, Ar), 129.76 (C, Ar), 129.15 (2× CH, Ar), 129.04 (2× CH, Ar), 128.35 (2× CH, Ar), 128.06 (2× CH, Ar), 119.09 (2× CH, Ar), 76.25 (C, C–OH), 57.04 (CH_2_-triazole), 48.18 (CH_2_–NH). HPLC (method B): 100%, RT = 4.77 min. HRMS (ESI), *m*/*z*. Calcd for C_24_H_21_Cl_2_N_5_O_4_S [M + H]^+^, 546.0769; found, 546.0769.

##### 4-((4-Chlorophenyl)sulfonamido)*-N*-(2-(2,4-dichlorophenyl)-2-hydroxy-3-(1*H*-1,2,4-triazol-1-yl)propyl)benzamide (14b, R^1^ = Cl, R^2^ = Cl, Y = S(O_2_))

Prepared from 4-amino-*N*-(2-(2,4-dichlorophenyl)-2-hydroxy-3-(1*H*-1,2,4-triazol-1-yl)propyl)benzamide (10b) (0.17 g, 0.42 mmoL) and 4-chlorobenzenesulfonyl chloride (0.13 g, 0.63 mmoL). Purified by gradient column chromatography eluting with 3.5% MeOH in CH_2_Cl_2._ Product obtained as a white solid, yield 0.2 g (83%). M.p. 228–230 °C. TLC (CH_2_Cl_2_–MeOH 9.5 : 0.5 v/v), *R*_f_ = 0.46. ^1^H NMR (DMSO-d_6_): *δ* 10.76 (br.s, 1H, N*H*SO_2_), 8.40 (t, *J* = 6.0 Hz, 1H, NH), 8.32 (s, 1H, triazole), 7.79 (d, *J* = 8.8 Hz, 2H, Ar), 7.72 (s, 1H, triazole), 7.60 (dd, *J* = 4.8, 8.9 Hz, 4H, Ar), 7.54 (dd, *J* = 3.2, 5.5 Hz, 2H, Ar), 7.27 (dd, *J* = 2.2, 8.7 Hz, 1H, Ar), 713 (d, *J* = 8.8 Hz, 2H, Ar), 6.36 (s, 1H, OH), 5.04 (d, *J* = 14.4 Hz, 1H, C*Ha*Hb-triazole), 4.63 (d, *J* = 14.4 Hz, 1H, CHa*Hb*-triazole), 4.01 (dd, *J* = 5.7, 14.1 Hz, 1H, C*Ha*HbNH), 3.89 (dd, *J* = 6.3, 14.0 Hz, 1H, CHa*Hb*NH). ^13^C NMR (DMSO-d_6_): *δ* 167.78 (C, C=O), 151.00 (CH, triazole), 145.56 (CH, triazole), 141.01 (C, Ar), 138.65 (C, Ar), 138.48 (C, Ar), 138.15 (C, C–Cl), 133.26 (C, C–Cl), 132.01 (C, C–Cl), 131.50 (CH, Ar), 130.41 (CH, Ar), 130.03 (2× CH, Ar), 129.44 (C, Ar), 129.26 (2× CH, Ar), 129.08 (2× CH, Ar), 127.27 (CH, Ar), 119.02 (2× CH, Ar), 76.91 (C, C–OH), 45.09 (CH_2_-triazole), 45.96 (CH_2_–NH). HPLC (method B): 99.7%, RT = 4.76 min. HRMS (ESI), *m*/*z*. calcd for C_24_H_20_Cl_3_N_5_O_4_S [M + H]^+^, 580.038; found, 580.0304.

### CaCYP51 susceptibility testing

Antifungal MIC values were obtained according to the CLSI microdilution method for yeasts.^28^*C. albicans* strains CA14 and SC5314 were tested in triplicate. Cultures were diluted to 2.5 × 10^3^ cells per mL in RPMI 1640 (Sigma), buffered with 0.165 M MOPS, pH 7.0. Antifungal compounds were dissolved in DMSO and added at a final concentration of 1% v/v DMSO. Plates were incubated at 37 °C and read at 48 h.

### CaCYP51 IC_50_

*C. albicans* CYP51 was overexpressed in *E. coli* and purified and CaCYP51 reconstitutions assays performed.^10,37^ The final reaction volume of 500 μL, contained; 1 μM *C. albicans* CYP51, 2 μM *Homo sapiens* cytochrome P450 reductase (CPR) (UniProtKB accession number P16435), 60 μM lanosterol, 50 μM dilaurylphosphatidylcholine, 4% w/v 2-hydroypropyl-cyclodextrin, 0.4 mg mL^−1^ isocitrate dehydrogenase, 25 mM trisodium isocitrate, 50 mM NaCl, 5 mM MgCl_2_, and 40 mM MOPS (morpholinepropanesulfonic acid, pH 7.2). Antifungal compounds were added in 2.5 μL DMSO. The reaction was started by the addition of 4 mM NADPH-tetrasodium salt. Samples were shaken for 15 min at 37 °C and sterol metabolites extracted with ethyl acetate and derivatised with BSTFA.

### Binding affinity (*K*_d_)

The ligand–CaCYP51 complex was determined by non-linear regression (Levenberg–Marquardt algorithm) using a rearrangement of the Morrison equation for tight ligand binding,^38^ while weak ligand binding was calculated by the Michaelis–Menten equation. Curve fitting for novel azole saturation curves was performed using the computer program ProFit 6.1.12 (QuantumSoft, Zurich, Switzerland) for Mac OSX. *K*_d_ values were determined for each of the three replicate titrations per azole compound and then mean *K*_d_ values and standard deviations calculated.

### Preparation of *S. cerevisiae* strain Y2760

The *HsCYP51* ORF (NP_000777.1) was codon harmonized (ch) using DNA 2.0 for expression in *S. cerevisiae* and synthesized by ATUM (Newark, Ca, USA). The 3′ end of the ORF was extended to encode EQKI of the ScErg11 C-terminus, followed by a GGR linker and a 6xHis tag. Strain Y2514 was prepared by transforming host strain Y1857 with a transformation cassette including the above construct together with the *ScPGK* transcription terminator and *URA3* selection marker located downstream, bordered by upstream and downstream parts of *ScPDR5* gene identical to sequences in ScErg11 over-expressing strain Y941. Strain Y2514 constitutively overexpressed *HsCYP51ch-6xHis* and *URA3* from the *ScPDR5* locus.

Strain Y2757 (Y2514, *PDR15::HsCPRch-6xHis-LoxP-HIS1-LoxP*) was prepared by co-expressing the cognate reductase HsCRP in strain Y2514. Codon harmonisation and synthesis of human NADPH-cytochrome P450 reductase isoform 2 (HsCPRch, NP_001369584.1) ORF was performed by ATUM. The gene was 3′ extended with a sequence encoding GGR-6xHIS. A transformation cassette was prepared by recombinant PCR to include the *HsCPRch* ORF with 657 nucleotides the *ScPDR5* promoter upstream, and the *ScPGK* terminator followed by the *LoxP* flanked *ScHIS1* selection marker downstream. *ScPDR15* specific arms bordering the construct enabled the cassette to be integrated between the nucleotide 441 of the 5′ UTR and nucleotide 1 of the 3′ UTR in *PDR15*.

Strain Y2758 (Y2757 *ΔHIS1*) was made using the following steps. Strain Y2757 was transformed with the pSH69 pGAL1-cre plasmid (Euroscarf, Germany) *via* selection on hygromycin (75 μg mL^−1^) containing YPD plates. A single hygromycin positive colony was inoculated in 1% yeast extract, 2% peptone and 2% of galactose broth and incubated for 16 hours at 30 °C with shaking. The diluted culture was then plated on YPD agar and replica-plated on YPD + hygromycin and SD-HIS dropout agar plates. Deletion of the *HIS* selection marker downstream of HsCPRch-6xHis in the *PDR15* locus was confirmed in double His and hygromycin negative isolates by PCR and DNA sequence analysis.

The endogenous *ScERG11* ORF in strain Y2758 was removed by transforming with a *LoxP- ScHIS1* containing cassette with arms complementing from +387 nucleotides in the 5′UTR and −60 nucleotides in the 3′UTR of the *ScERG11* locus, resulting in strain Y2760 (ADΔΔ *PDR5::HsCYP51ch-6×HIS-URA3; PDR15::HsCPRch6×HIS-LoxP, ΔERG11-LoxP-HIS1-LoxP*).

### Antifungal susceptibility testing of recombinant *S. cerevisiae* strains expressing CYP51

Minimum inhibitory concentration (MIC) were determined according to recommendations outlined in the Clinical and Laboratory Standards Institute (CLSI) document M27-Ed4E^39^ with modifications. Sabouraud dextrose (SD) medium (pH 6.8) was used instead of RPMI-1640 for inoculum and growth of the *S. cerevisiae* strains and cultures were incubated at 30 °C. Assays were performed in round-bottomed 96-well plates (Corning, NY, US) and MICs were measured as the lowest concentrations of each antifungal agent that resulted in 80% inhibition of growth as compared with a drug-free, positive control. Stock solutions of each agent were prepared in DMSO and the final concentration of DMSO was 1% (v/v) in SD. The final concentration of fluconazole ranged from 0.25 to 128 μg mL^−1^. The final concentration of all other compounds ranged from 0.03–16 μg mL^−1^. Three biological repeats were performed.

### Computational studies

CaCYP51 protein–ligand complexes of all final compounds (*R*- and *S*-enantiomers) were obtained with Molecular Operating Environment (MOE) software^33^ using the crystal structure of wild-type CaCYP51 (PDB 5FSA).^34^ Minimisations were performed with the MMFF94 force field (ligands) and partial charges were automatically calculated. The charge of the haem iron at physiological pH was set to 3^+^ (geometry d2sp3) through the atom manager in MOE. Results were refined using the MMFF94 force field and scored by applying the London Δ*G* scoring function. The output database dock file was created with different poses for each ligand and arranged according to the final *S*-score function, which is the score of the last stage that was not set to zero. Molecular dynamics (MD) simulations were performed on CaCYP51-ligand complexes, which were optimised with the protein preparation wizard in Maestro (Schrödinger release 2020-1),^35^ by assigning bond orders, adding hydrogens, and correcting incorrect bond types. A default quick relaxation protocol was used to minimise the MD systems with the Desmond programme.^36^ The orthorhombic water box allowed for a 10 Å buffer region between protein atoms and box sides. Overlapping water molecules were deleted, and the systems were neutralised with Na^+^ ions and a salt concentration of 0.15 M. Force-field parameter assignment used the OPLS_2005 force field, for a 200 ns molecular dynamic run in the NPT ensemble (*T* = 300 K) at a constant pressure of 1 bar. Energy and trajectory atomic coordinate data were recorded at 1.2 ns intervals.

## Data availability

The data supporting this article have been included as part of the ESI.†

## Author contributions

MA and FAB performed the chemical synthesis and ET performed computational studies supervised by CS. AGW performed the binding affinity studies supervised by DEK and SLK. JEP performed the MIC and IC_50_ assays and BCM and MVK contributed to the *S. cerevisiae* model assays. All authors contributed to manuscript preparation.

## Conflicts of interest

There are no conflicts to declare.

## Supplementary Material

MD-016-D4MD00863D-s001
