# Supplementary material for: Exploring medium and long arm extensions of 1,2,4-triazole derivatives as Candida albicans 14α-demethylase (CYP51) inhibitors
Source: RSC Med Chem. 2025 Mar 12;16(5):2270–80. doi: 10.1039/d4md00863d (PMC11931565; doi:10.1039/d4md00863d)
Supplement: MD-016-D4MD00863D-s001 [file MD-016-D4MD00863D-s001.pdf]

Supplementary Information (SI) for RSC Medicinal Chemistry.
This journal is © The Royal Society of Chemistry 2025

Supporting Information

Exploring medium and long arm extensions of 1,2,4-triazole derivatives as *Candida albicans* 14α-demethylase (CYP51) inhibitors

Marwa Alsulaimany, Faizah A. Binjubair, Esra Tatar, Diane E. Kelly, Steven L. Kelly, Andrew G. Warrilow, Mikhail V. Keniya, Brian C. Monk, Josie E. Parker and Claire Simons

S2	**Figure S1 (part1)**. *CaCYP51 type II azole binding difference spectra*. Type II difference spectra are shown for the binding of **7a**, **7b**, **9a**, **9b**, **11b** and FLZ with 2.5 μM native CaCYP51 in quartz semi-microcuvettes of 1 cm light path. Each azole titration was performed in triplicate although only one replicate is shown.
S3	**Figure S1 (part2)**. *CaCYP51 type II azole binding difference spectra*. Type II difference spectra are shown for the binding of **12b**, **13a**, **13b**, 1**4b** and FLZ with 2.5 μM native CaCYP51 in quartz semi-microcuvettes of 1 cm light path. Each azole titration was performed in triplicate although only one replicate is shown.
S4	**Figure S2**. *CYP51 azole saturation curves*. Azole ligand binding saturation curves derived from the type II difference spectra with 2.5 μM native CaCYP51. Each azole titration was performed in triplicate although only one replicate is shown.
S5-S7	**Figure S3.** Comparing ligand-protein complex stability through protein-ligand RMSD over 200 ns MD simulation for exemplar compounds **7a, 7b, 9a, 9b, 11a, 11b, 12a, 12b, 13a, 13b, 14a** and 14**b** in the wild- type CaCYP51. Ligand RMSD in red and protein RMSD in blue.
S8-S11	**Figure S4.** Comparing the binding profile showing haem binding over 200 ns MD simulation for exemplar compounds **7a, 7b, 9a, 9b, 11a, 11b, 12a, 12b, 13a, 13b, 14a** and **14b** in wild- type CaCYP51. Interactions that occur more than 30.0% of the simulation time in the selected trajectory (0.00 through 200 ns) are shown.
S12-S15	**Figure S5.** Protein-ligand interactions of final frame after 200 ns MD simulation for enantiomers of **7a**, **7b**, **9a**, **9b**, **11a, 11b, 12a, 12b, 13a, 13b, 14a** and **14b** using wild type CaCYP51.
S16-22	**Figure S6**. 3D images illustrating binding position, haem Fe^{3+}-triazole binding distance and key binding interactions of protein-ligand interactions complexes of final frame after 200 ns MD simulation for enantiomers of **7a**, **7b**, **9a**, **9b**, **11a, 11b, 12a, 12b, 13a, 13b, 14a** and **14b** using wild type CaCYP51.
S23-S27	Procedures and characterisation of intermediate amines **5** and **10**
S28	**Figure S7**. ^{1}H NMR and ^{13}C NMR and HPLC trace of **6a**
S29	**Figure S8**. ^{1}H NMR and ^{13}C NMR and HPLC trace of **6b**
S30	**Figure S9**. ^{1}H NMR and ^{13}C NMR and HPLC trace of **7a**
S31	**Figure S10**. ^{1}H NMR and ^{13}C NMR and HPLC trace of **7b**
S32	**Figure S11**. ^{1}H NMR and ^{13}C NMR and HPLC trace of **11a**
S33	**Figure S12**. ^{1}H NMR and ^{13}C NMR of **11b**
S34	**Figure S13**. ^{1}H NMR and ^{13}C NMR of **12a**
S35	**Figure S14**. ^{1}H NMR and ^{13}C NMR of **12b**
S36	**Figure S15**. ^{1}H NMR and ^{13}C NMR of **13a**
S37	**Figure S16**. ^{1}H NMR and ^{13}C NMR of **13b**
S38	**Figure S17.** ^{1}H NMR and ^{13}C NMR and HPLC trace of **14a**
S39	**Figure S18.** ^{1}H NMR and ^{13}C NMR and HPLC trace of **14b**
S40	References

BINDING AFFINITY DATA

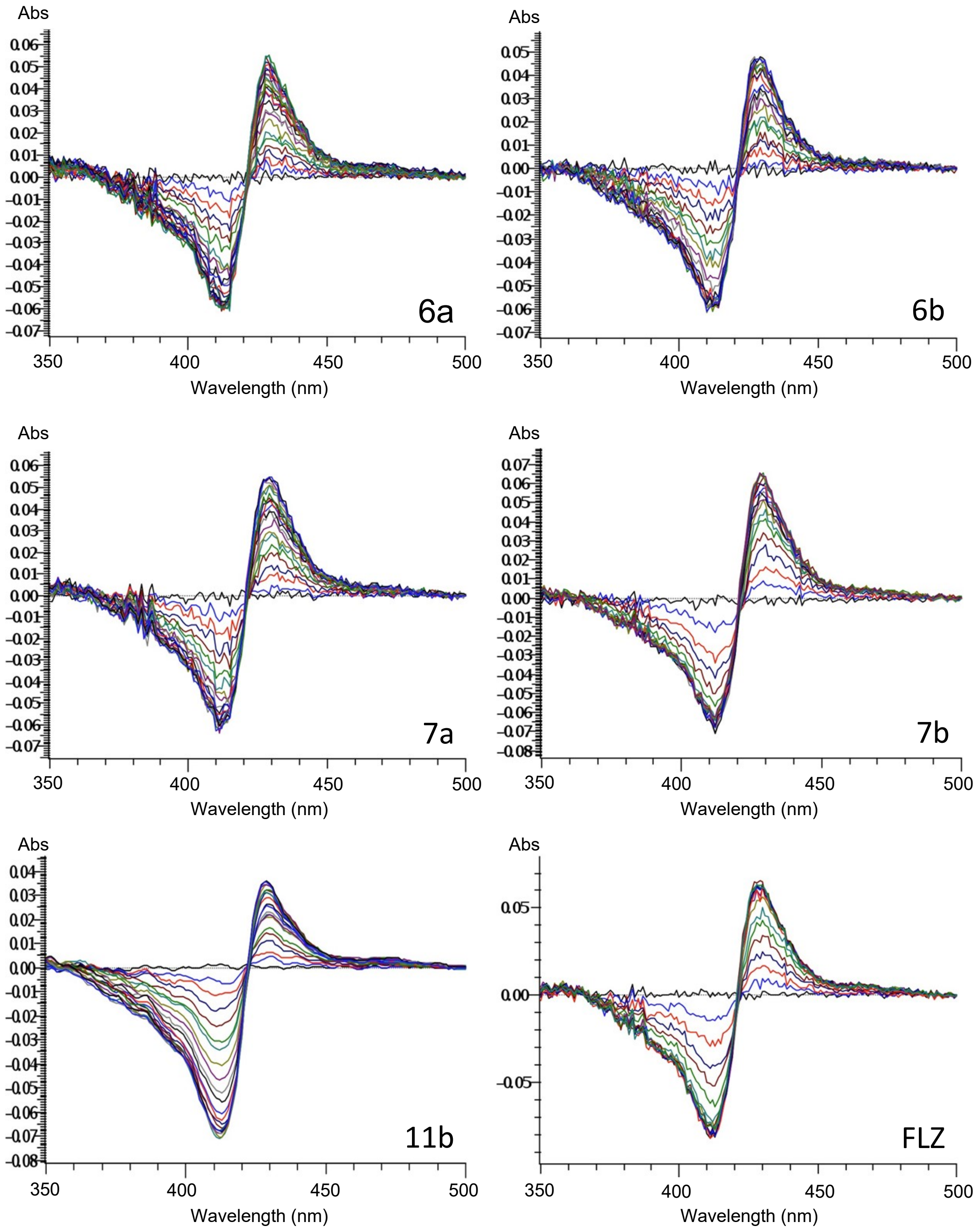

Figure S1 (part1). *CaCYP51 type II azole binding difference spectra*. Type II difference spectra are shown for the binding of **6a**, **6b**, **7a**, **7b**, **11b** and FLZ with 2.5 µM native CaCYP51 in quartz semi-microcuvettes of 1 cm light path. Each azole titration was performed in triplicate although only one replicate is shown.

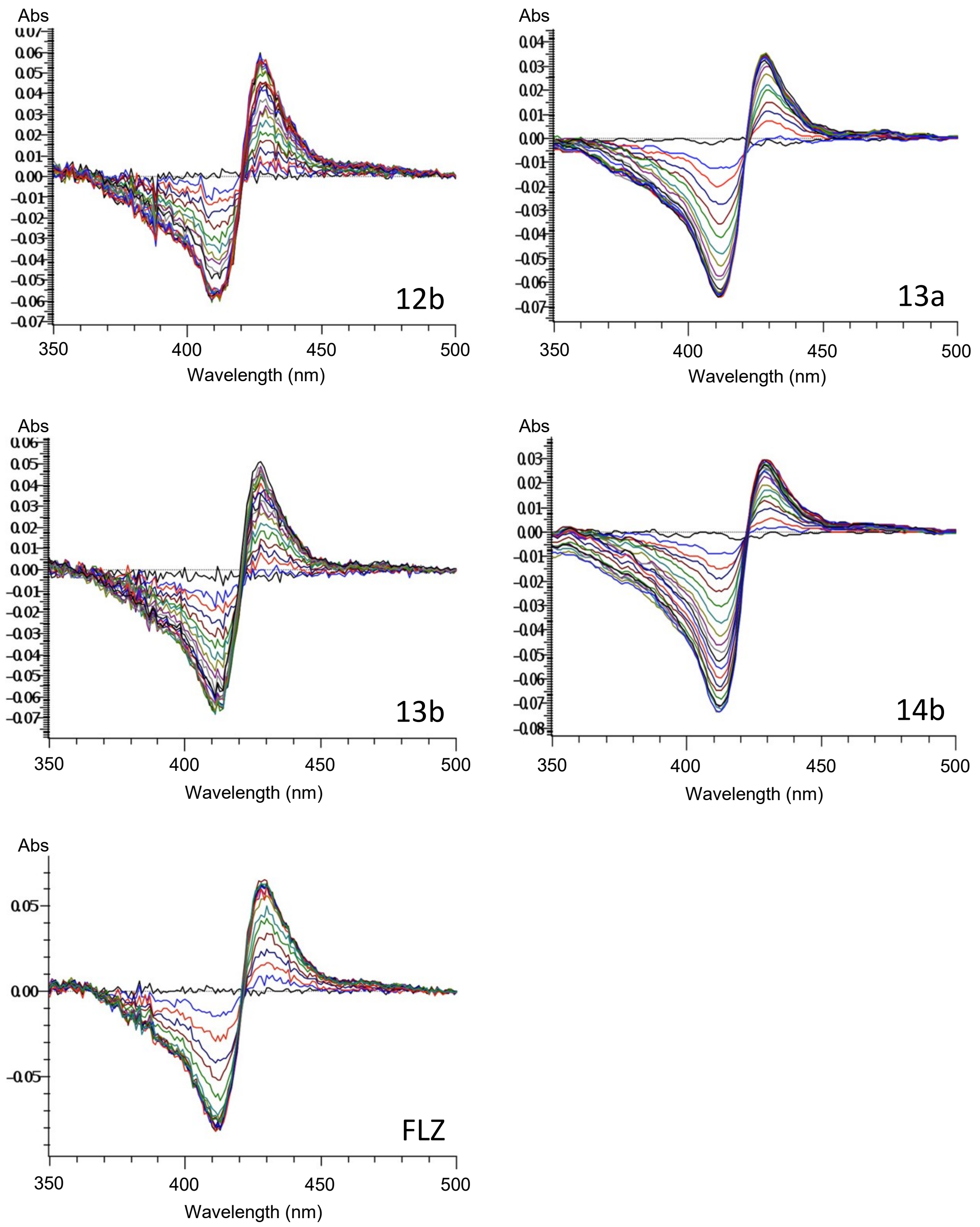

Figure S1 (part2). *CaCYP51 type II azole binding difference spectra*. Type II difference spectra are shown for the binding of **12b**, **13a**, **13b**, **14b** and FLZ with 2.5 μM native CaCYP51 in quartz semi-microcuvettes of 1 cm light path. Each azole titration was performed in triplicate although only one replicate is shown.

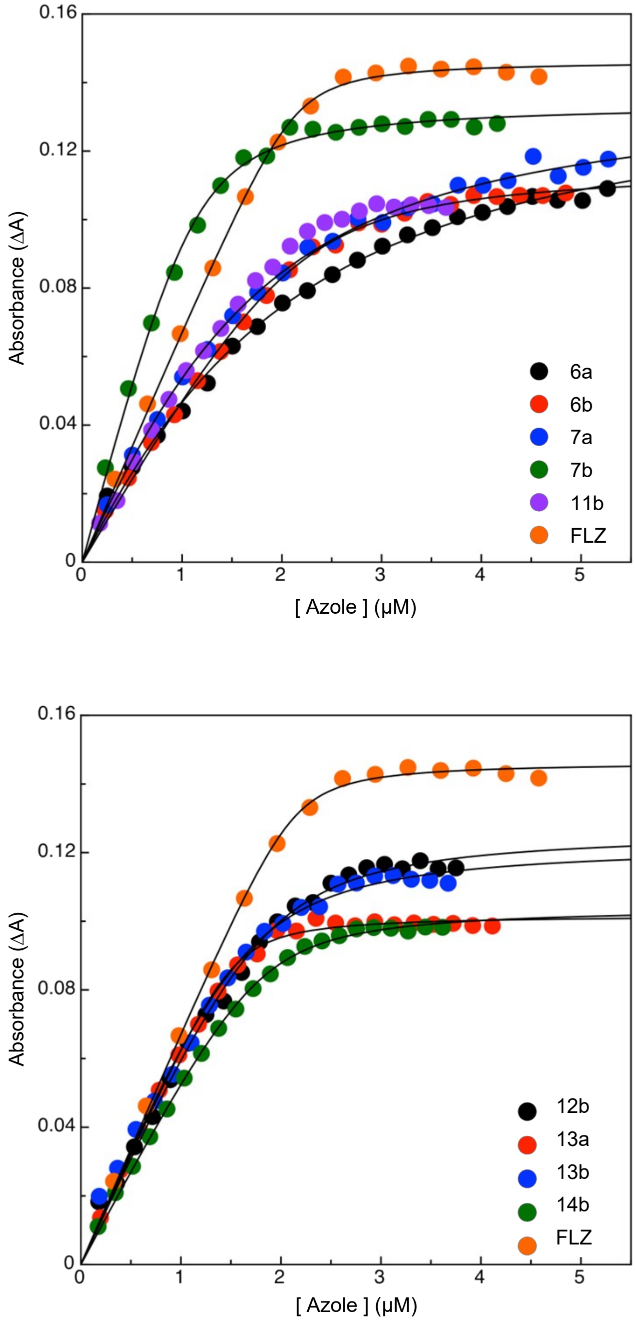

Figure S2. *CYP51 azole saturation curves*. Azole ligand binding saturation curves derived from the type II difference spectra with 2.5 μM native CaCYP51. Each azole titration was performed in triplicate although only one replicate is shown.

COMPUTATIONAL DATA

R-6a

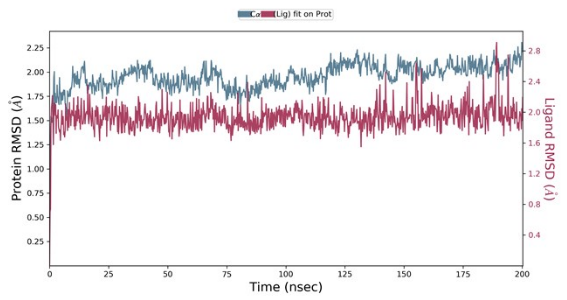

S-6a

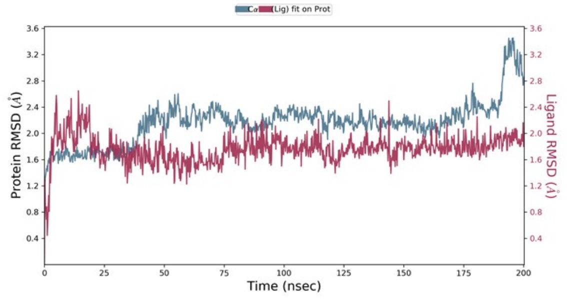

R-6b

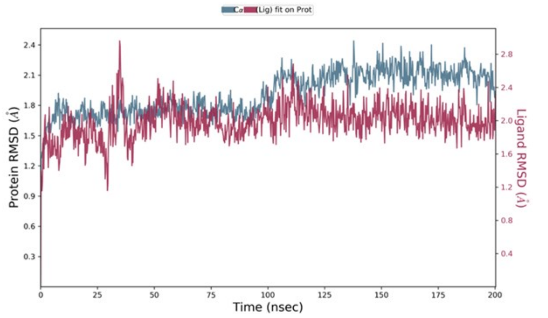

S-6b

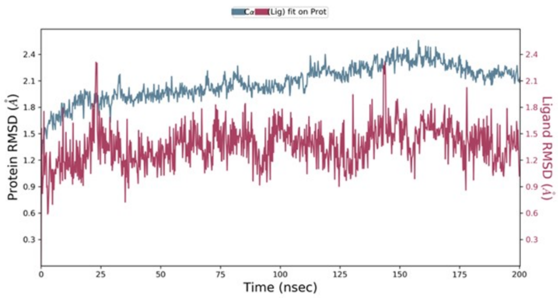

R-7a

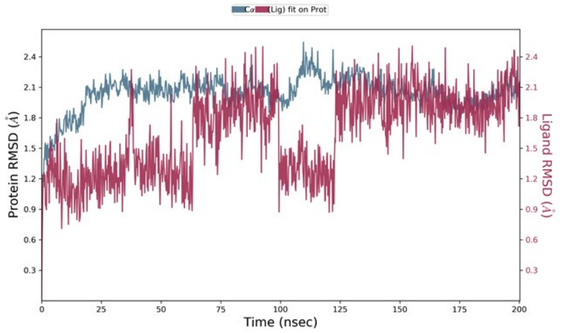

S-7a

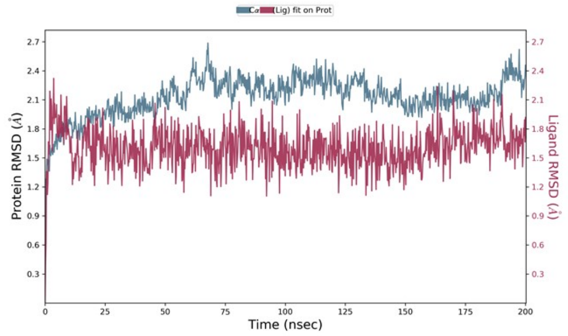

R-7b

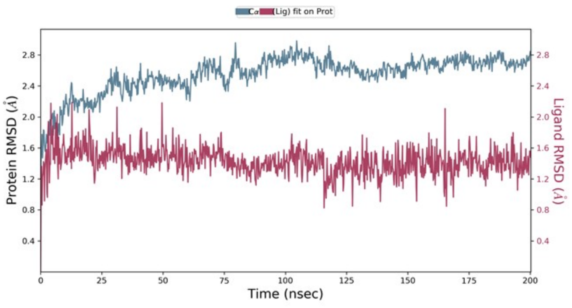

S-7b

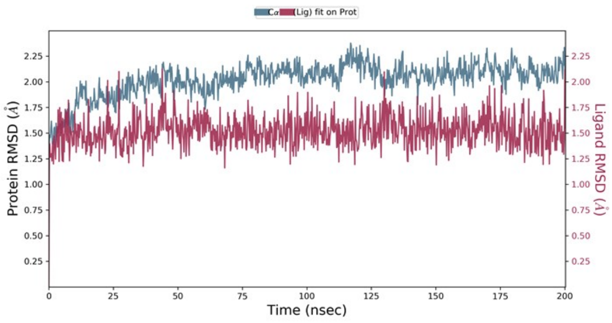

R-11a

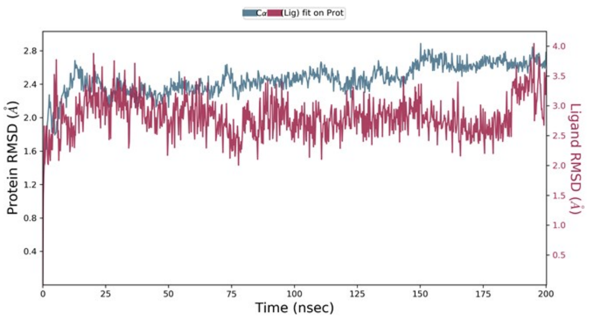

S-11a

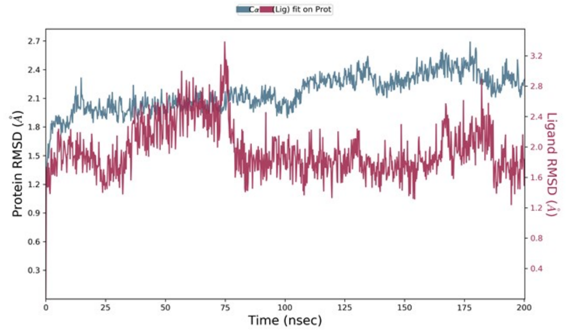

R-11b

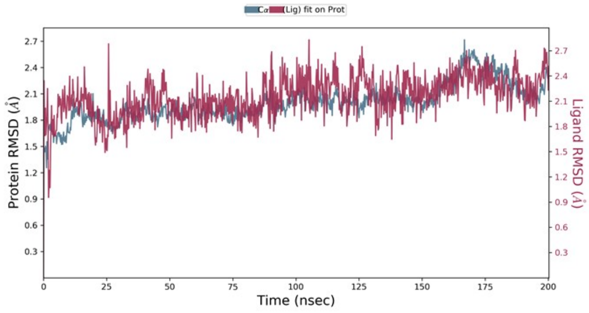

S-11b

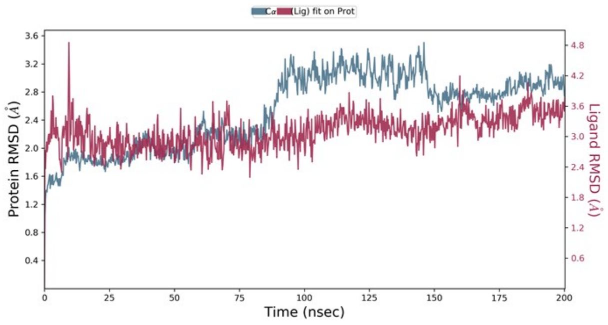

R-12a

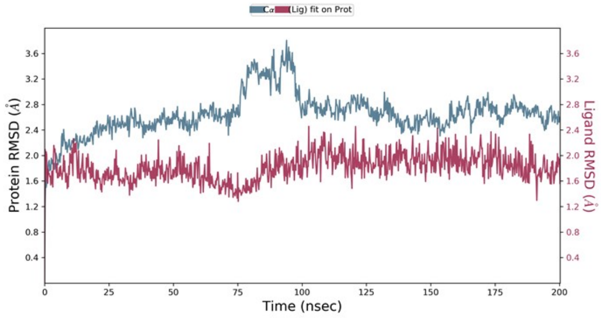

S-12a

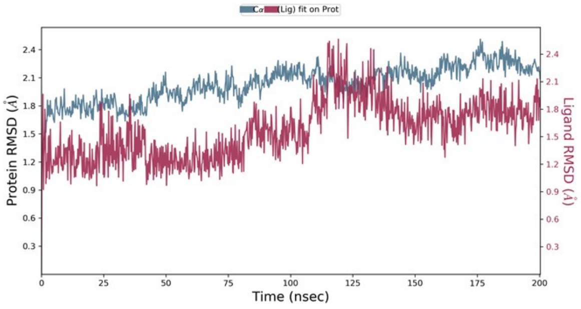

R-12b

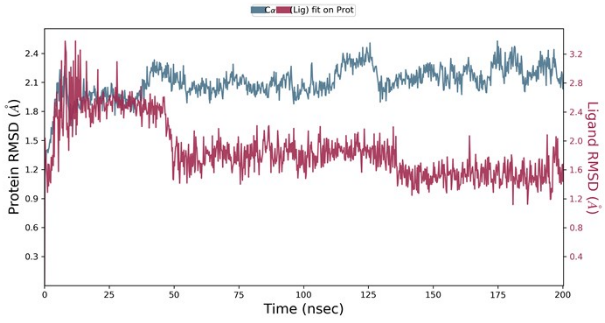

S-12b

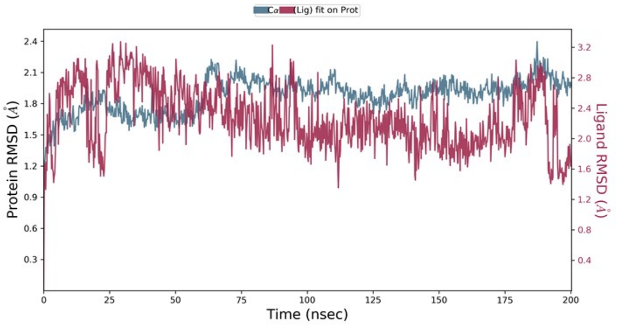

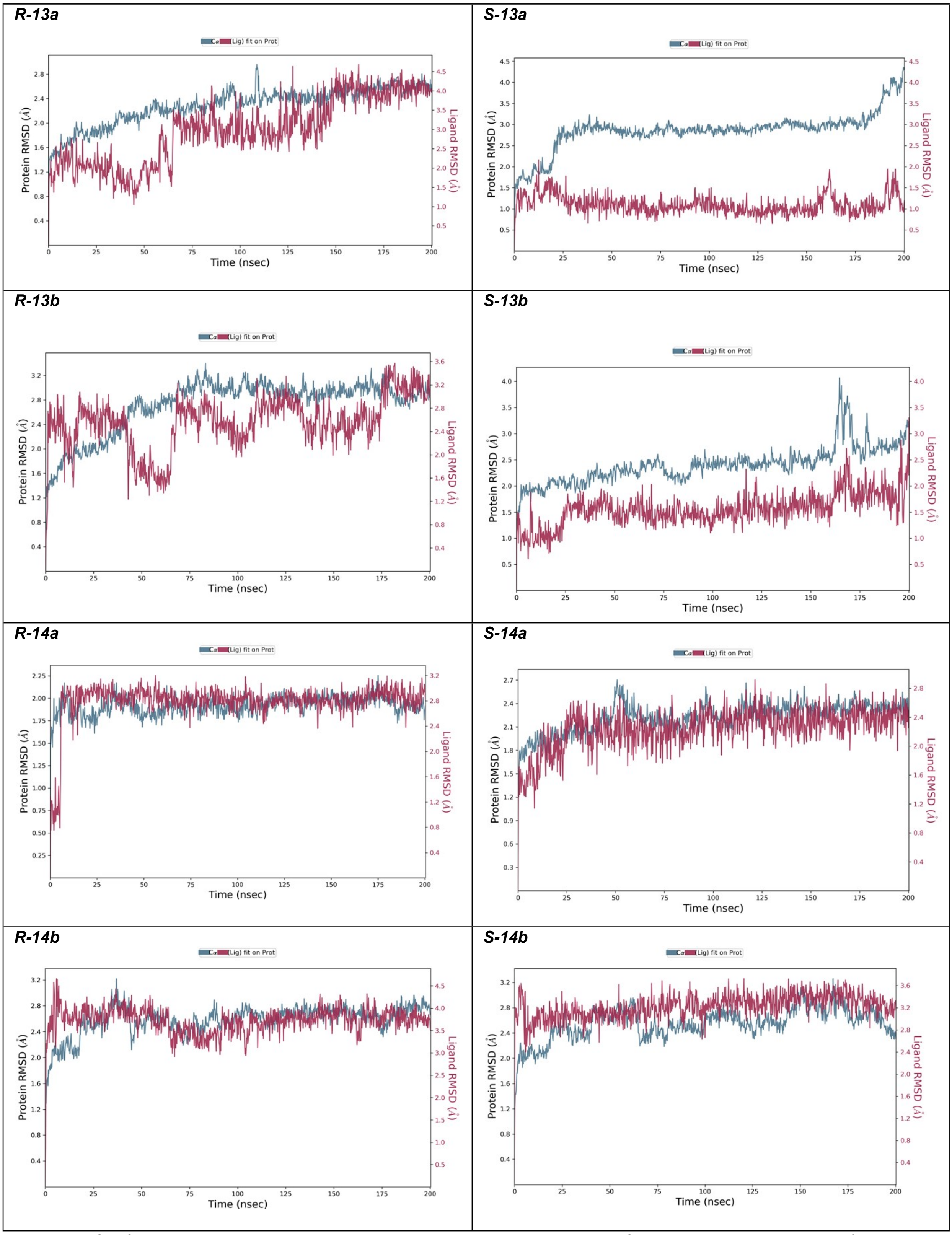

Figure S3. Comparing ligand-protein complex stability through protein-ligand RMSD over 200 ns MD simulation for exemplar compounds **6a, 6b, 7a, 7b, 11a, 11b, 12a, 12b, 13a, 13b, 14a** and **14b** in the wild- type CaCYP51. Ligand RMSD in red and protein RMSD in blue.

R-6a

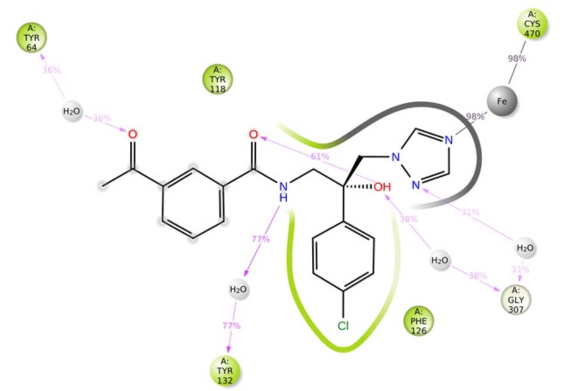

S-6a

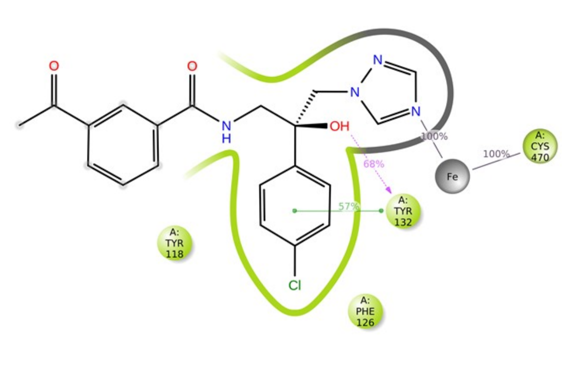

R-6b

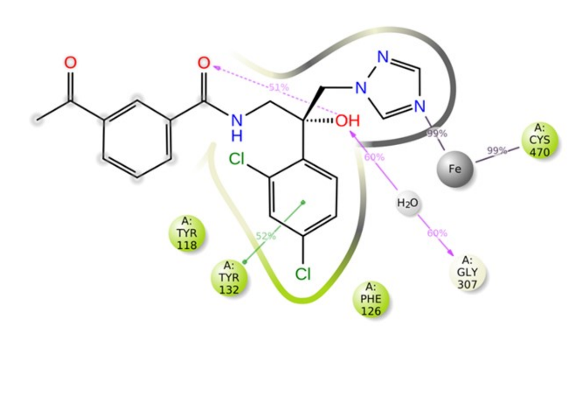

S-6b

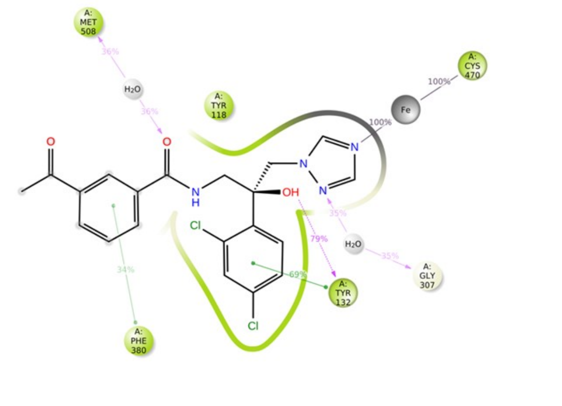

R-7a

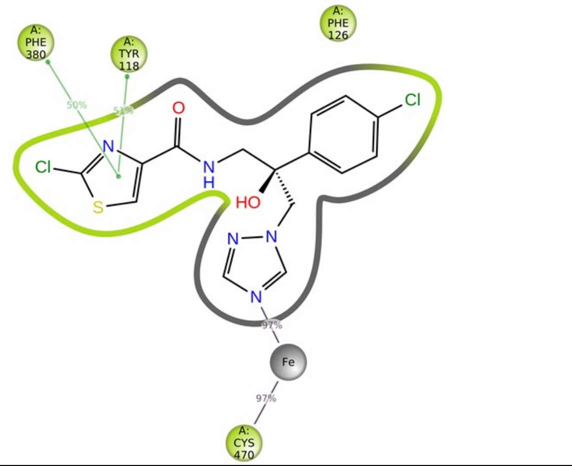

S-7a

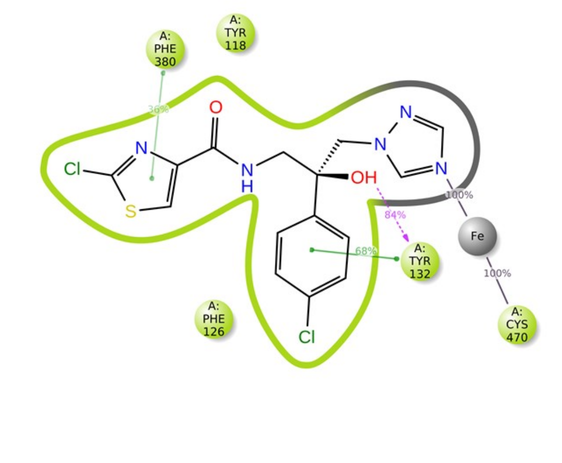

R-7b

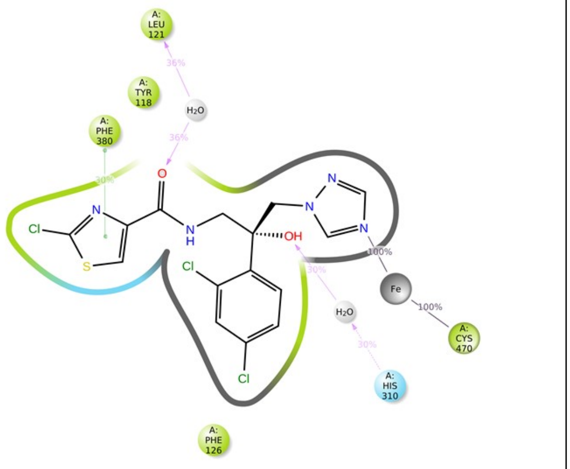

S-7b

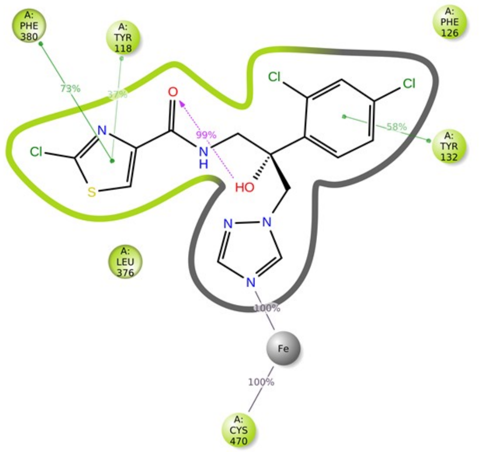

R-11a

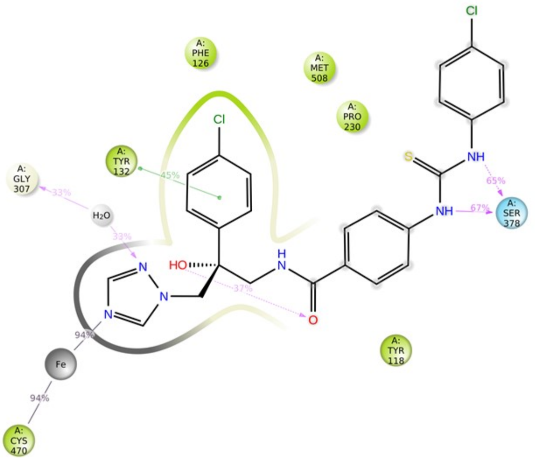

S-11a

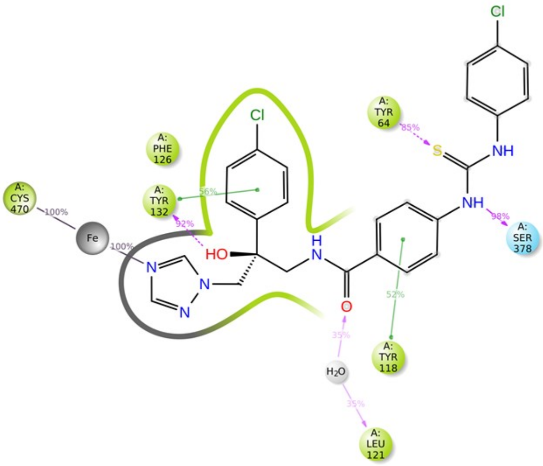

R-11b

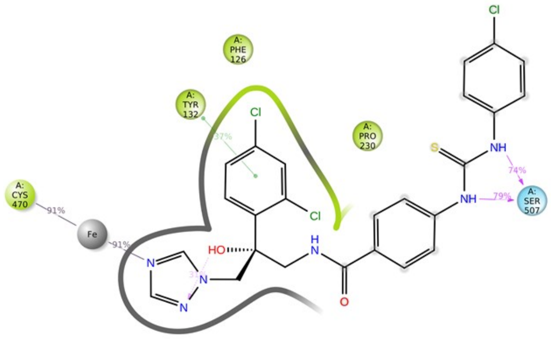

S-11b

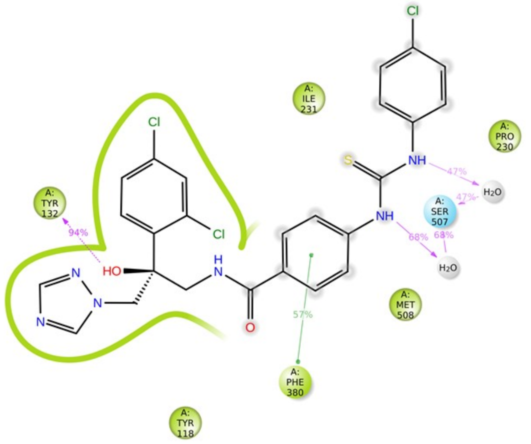

R-12a

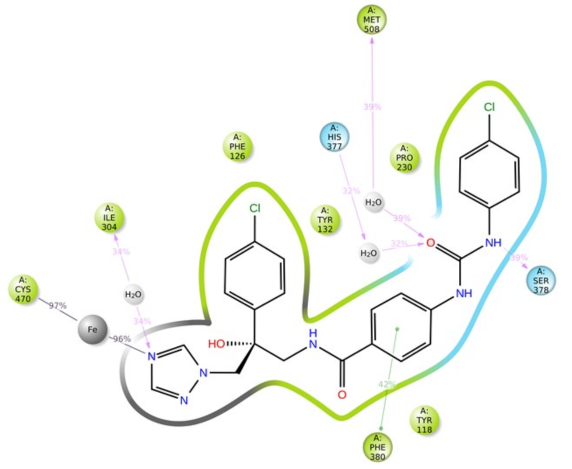

S-12a

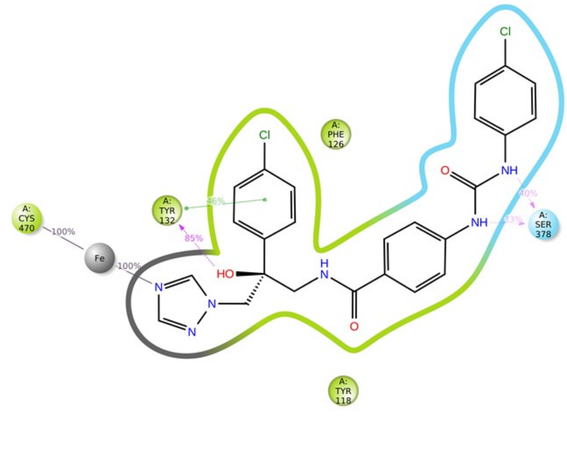

R-12b

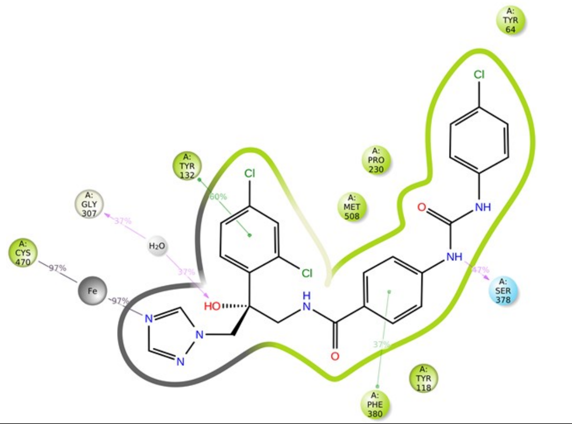

S-12b

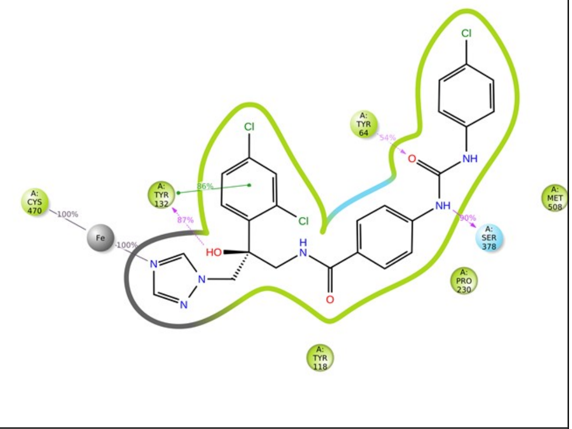

R-13a

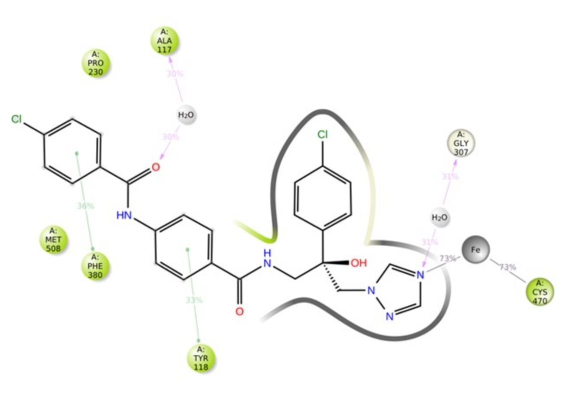

S-13a

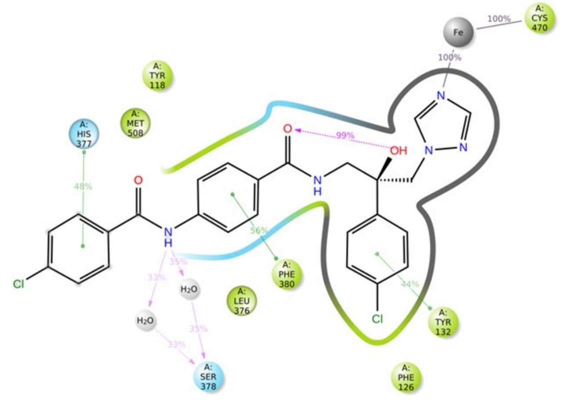

R-13b

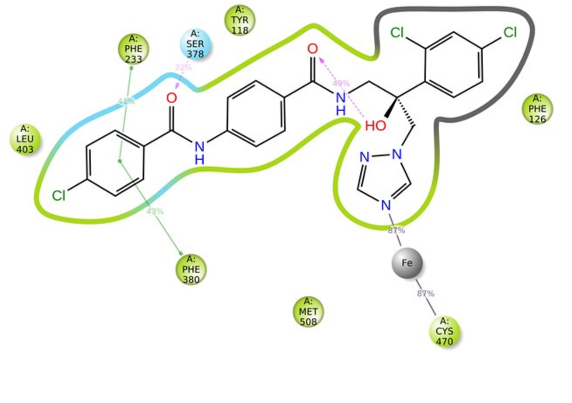

S-13b

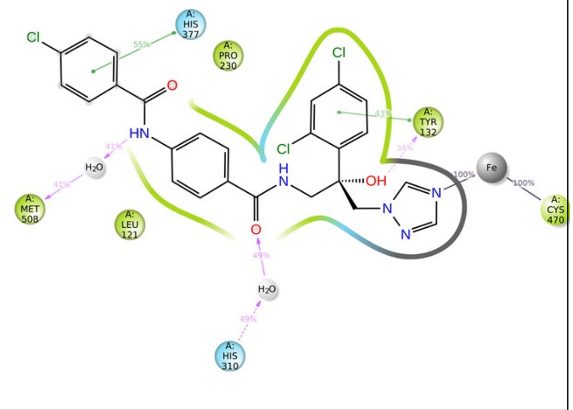

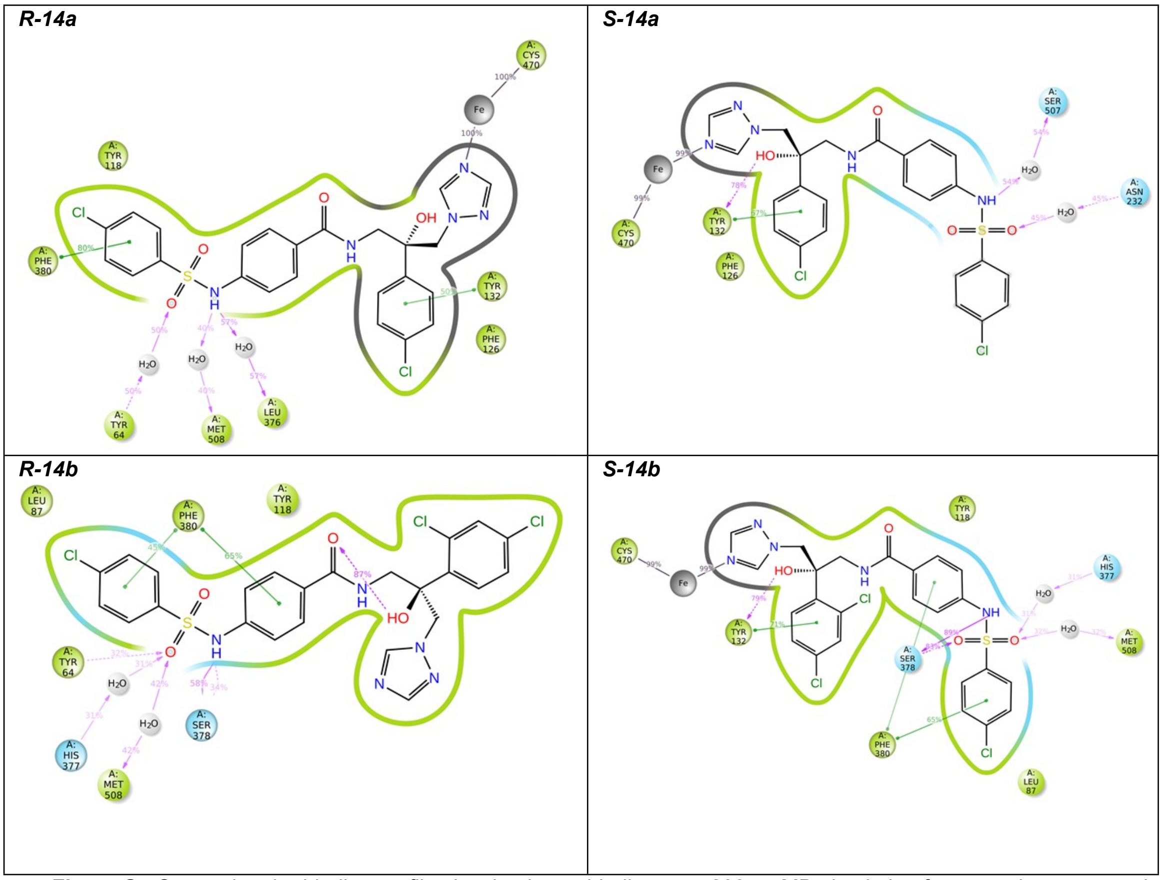

Figure S4 Comparing the binding profile showing haem binding over 200 ns MD simulation for exemplar compounds **6a, 6b, 7a, 7b, 11a, 11b, 12a, 12b, 13a, 13b, 14a** and **14b** in wild- type CaCYP51. Interactions that occur more than 30.0% of the simulation time in the selected trajectory (0.00 through 200 ns) are shown.

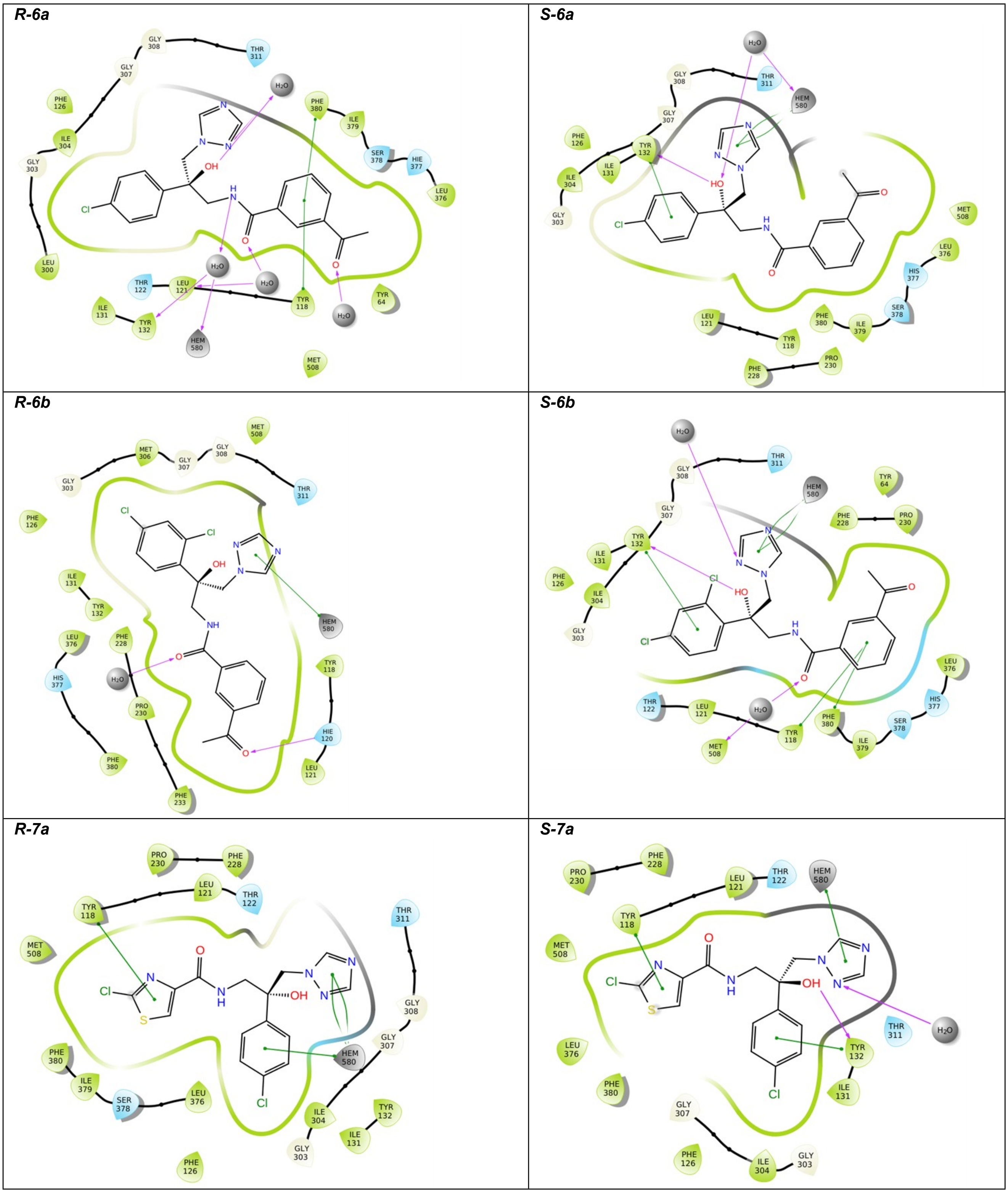

R-6a
GLY 308
THR 311
GLY 307
H₂O
PHE 126
PHE 380
ILE 379
ILE 304
SER 378
HIE 377
GLY 303
LEU 376
OH
Cl
LEU 300
THR 122
LEU 121
TYR 118
TYR 64
ILE 131
TYR 132
HEM 580
MET 508
S-6a
GLY 308
THR 311
HEM 580
GLY 307
PHE 126
TYR 132
ILE 131
ILE 304
HO
GLY 303
MET 508
LEU 376
HIS 377
SER 378
LEU 121
PHE 380
ILE 379
TYR 118
PHE 228
PRO 230
R-6b
MET 508
MET 306
GLY 308
GLY 307
GLY 303
THR 311
PHE 126
OH
ILE 131
TYR 132
HEM 580
LEU 376
PHE 228
HIS 377
TYR 118
PRO 230
HIE 120
PHE 380
LEU 121
PHE 233
S-6b
THR 311
GLY 308
HEM 580
TYR 64
GLY 307
PHE 228
PRO 230
TYR 132
ILE 131
PHE 126
HO
ILE 304
GLY 303
LEU 376
THR 122
LEU 121
HIS 377
SER 378
PHE 380
TYR 118
MET 508
ILE 379
R-7a
PRO 230
PHE 228
LEU 121
THR 122
TYR 118
THR 311
MET 508
OH
GLY 308
GLY 307
HEM 580
PHE 380
ILE 379
SER 378
LEU 376
ILE 304
TYR 132
ILE 131
GLY 303
PHE 126
S-7a
PRO 230
PHE 228
LEU 121
THR 122
HEM 580
TYR 118
MET 508
OH
THR 311
LEU 376
TYR 132
PHE 380
ILE 131
GLY 307
PHE 126
ILE 304
GLY 303

R-7b

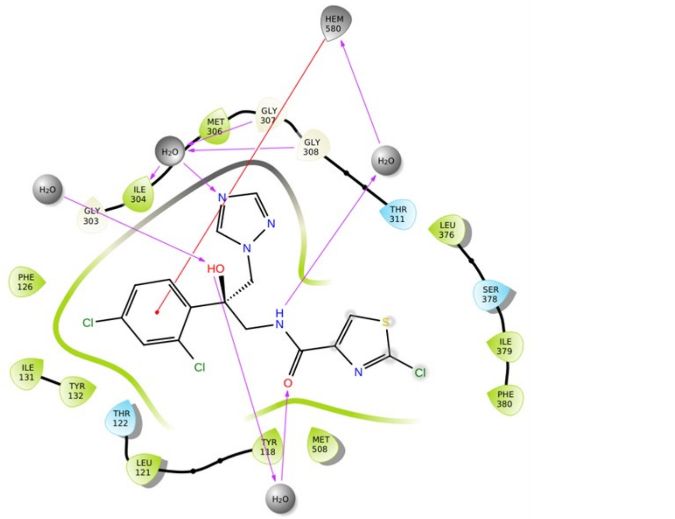

S-7b

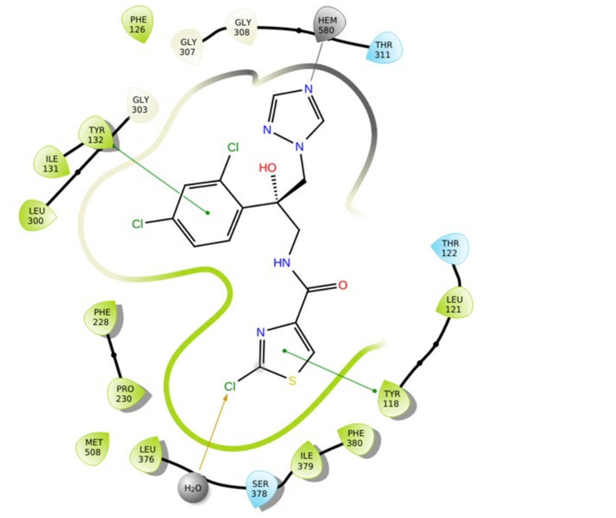

R-11a

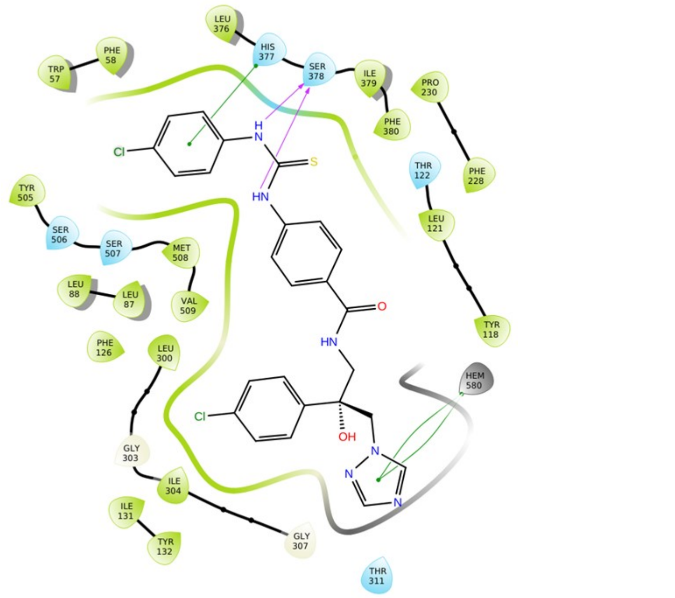

S-11a

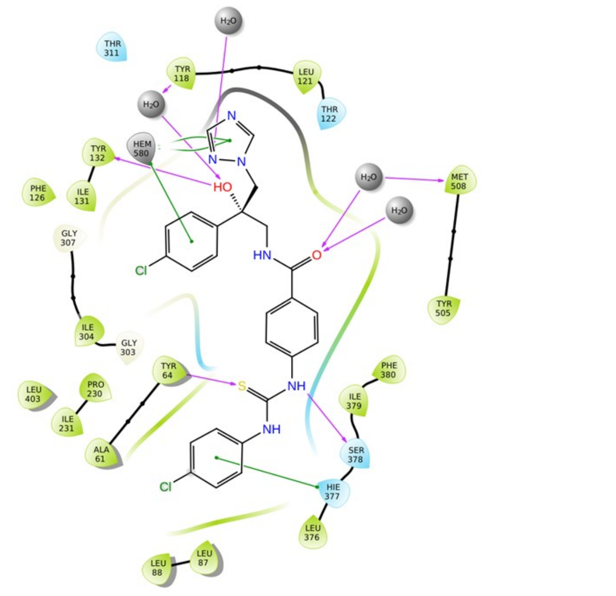

R-11b

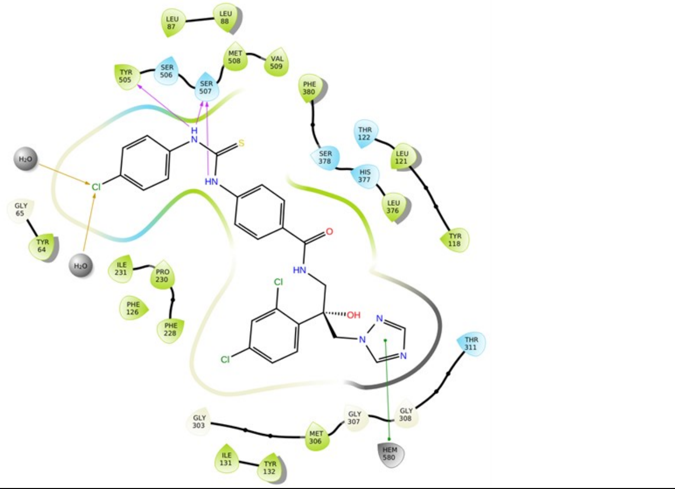

S-11b

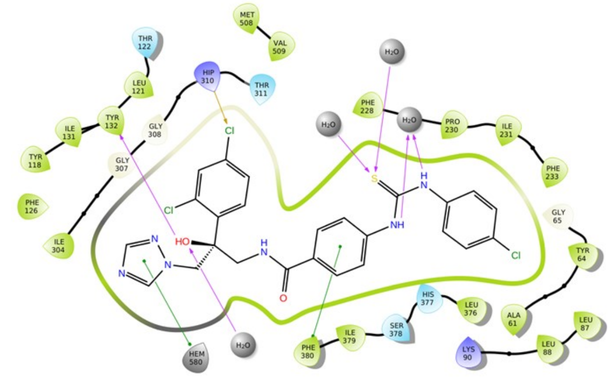

R-12a

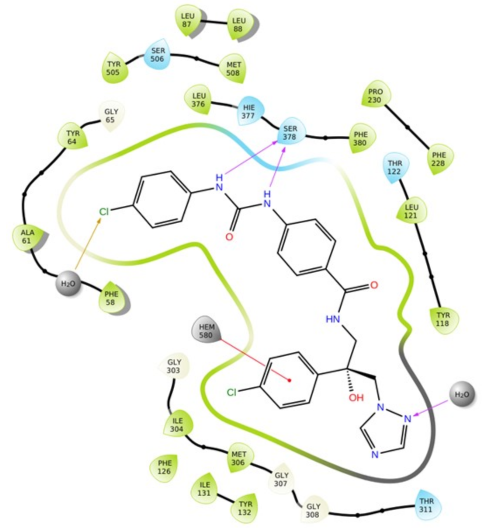

S-12a

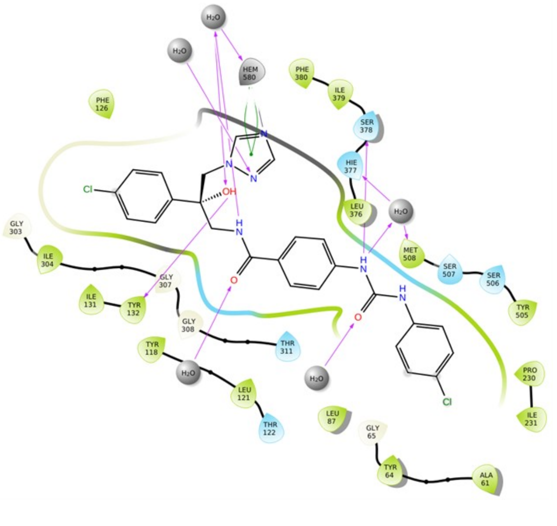

R-12b

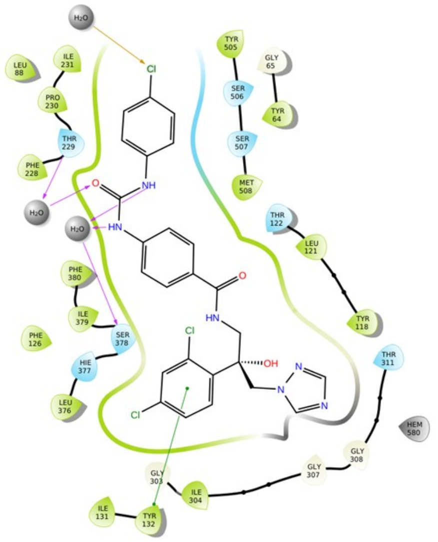

S-12b

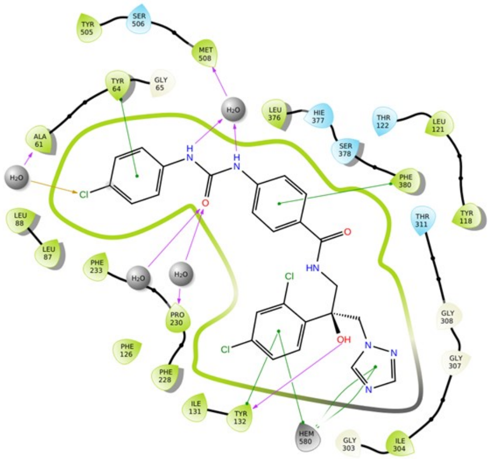

R-13a

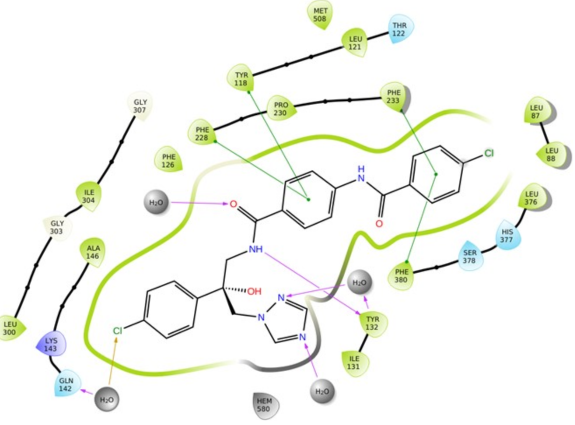

S-13a

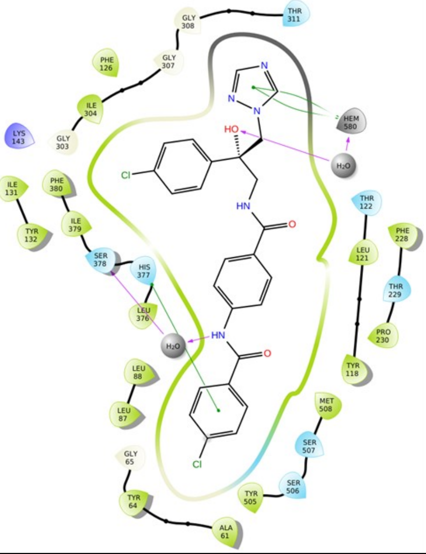

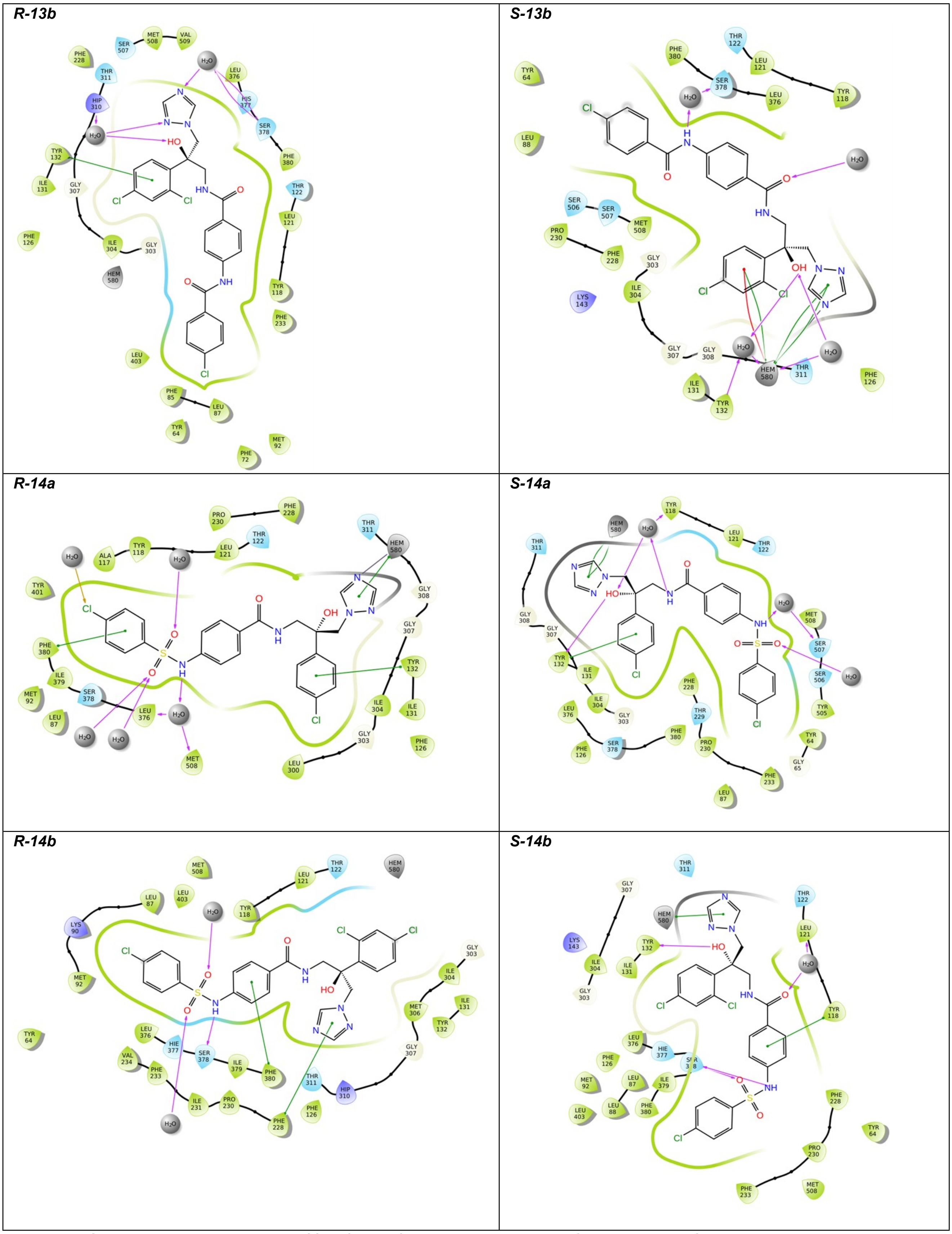

Figure S5. Protein-ligand interactions of final frame after 200 ns MD simulation for enantiomers of **6a**, **6b**, **7a**, **7b**, **11a**, **11b**, **12a**, **12b**, **13a**, **13b**, **14a** and **14b** using wild type CaCYP51.

Cmpd	3D CaCYP51-ligand complex after 200ns MD	Fe-haem	Key interactions

	simulation	distance (Å)	
***R*-6a**	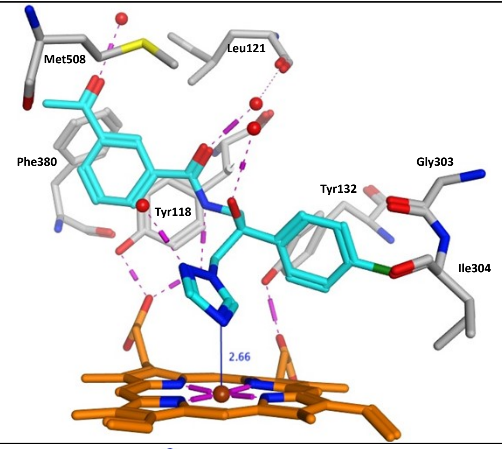	2.66*	Amide - H_2O mediated H-bond Leu121 and Tyr 132<br><br>Acetyl *benzene* ring – π-π stacking Tyr118 and Phe380<br><br>O*H*-H_2O H-bond
***S*-6**	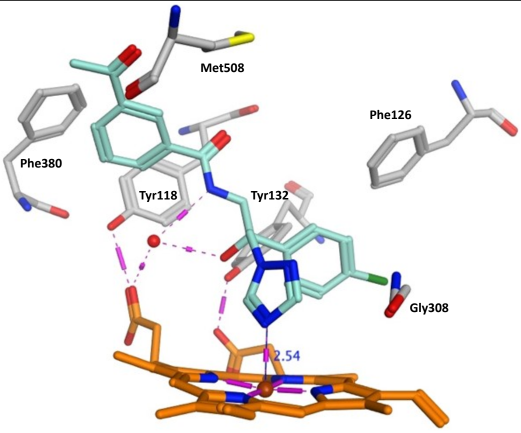	2.54	*O*H and amide NH – H_2O mediated with haem<br><br>O*H*- Tyr132 H-bond<br><br>Cl-*benzene* - π-π face-edge Tyr132
***R*-6b**	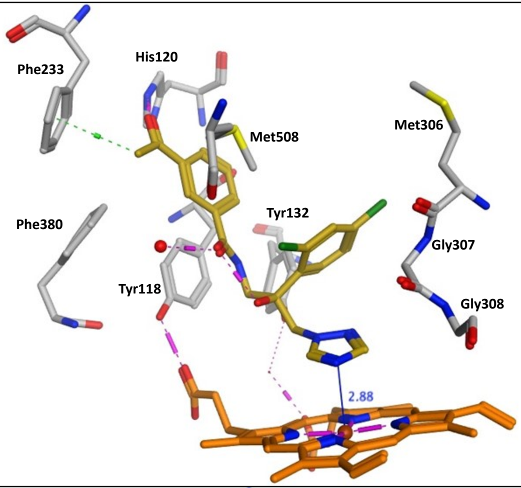	2.88*	Amide C=*O* and OH – H_2O H-bond<br><br>Acetyl C=*O* – His120 H-bond<br><br>Acetyl CH_3 – VdW Phe233
***S*-6b**	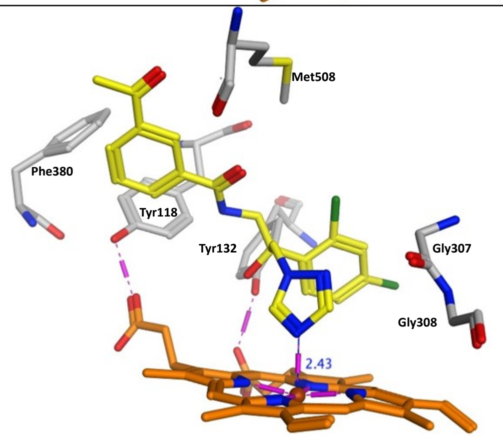	2.43	Acetyl *benzene* ring – π-π stacking Tyr118 and Phe380<br><br>diCl-*benzene* - π-π face-edge Tyr132<br><br>O*H*- Tyr132 H-bond

***R*-7a**	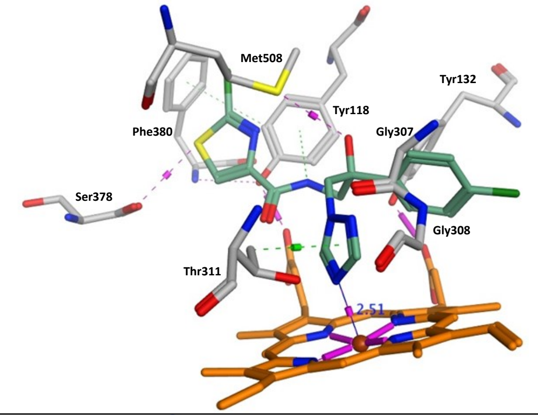	2.51	Thiazole S – Ser378 H-bond<br><br>Amide NH-Tyr118 (VdW cation/π)<br><br>OH-Met508 H-bond<br><br>Triazole – Thr311 VdW
***S*-7a**	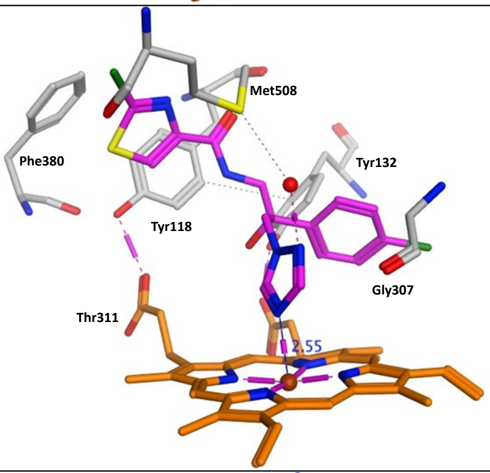	2.55	Thiazole – Tyr118 π-π stacking<br><br>O*H*-Tyr132 H-bonding<br><br>Cl-benzene – π-π face-edge Tyr132
***R*-7b**	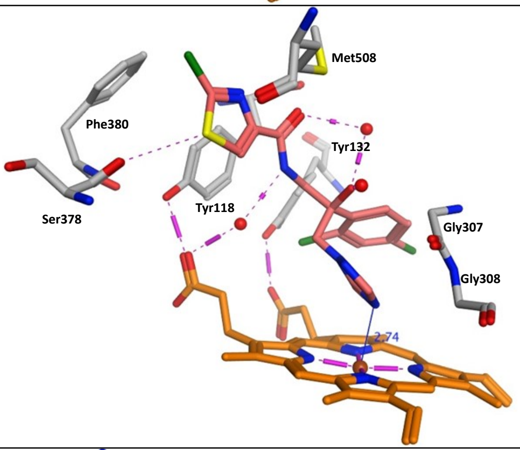	2.74*	H-bonding interactions H_2O and OH and amide NH and C=O<br><br>Thiazole S – Ser378
***S*-7b**	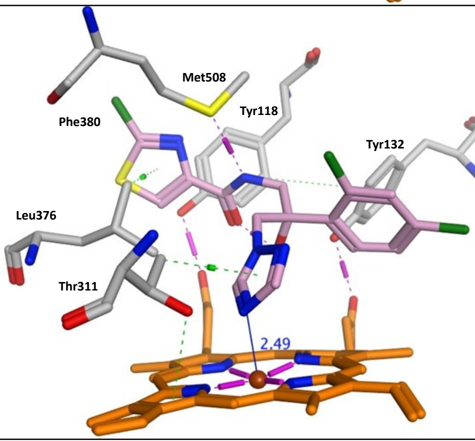	2.49	Thiazole – Tyr118 π-π stacking and Leu376 VdW<br><br>Triazole – Thr311 VdW<br><br>Amide NH – Met508 H-bond

***R*-11a**	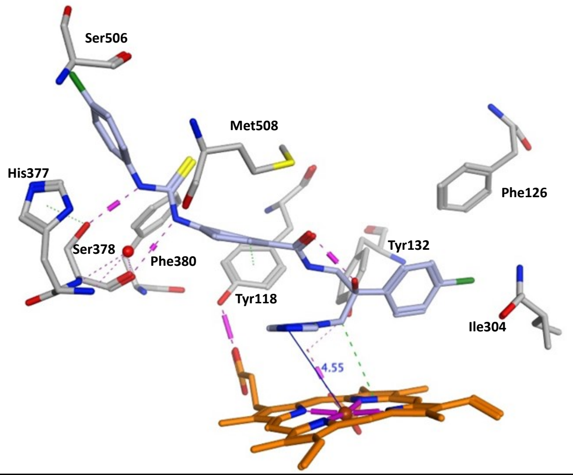	4.55*	Thiourea 2 x NH – Ser378 H-bonding<br><br>Central aryl ring – Tyr118 π-π face-edge
***S*-11a**	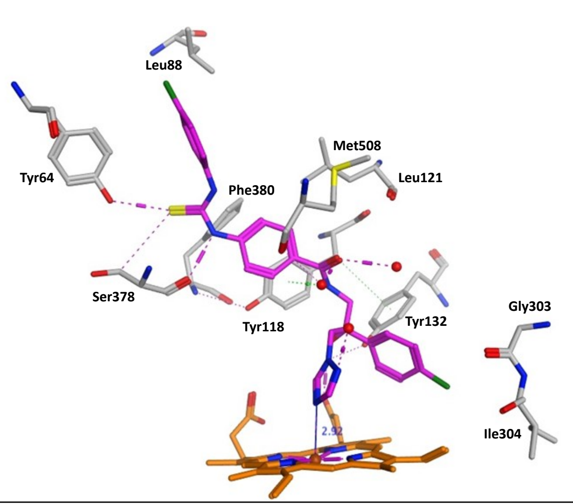	2.92	Thiourea 2 x NH – Ser378 H-bonding<br><br>C=*S* – Tyr64 H-bonding<br><br>Amide NH – Tyr118 VdW<br><br>Central aryl ring – Tyr118 π-π stacking<br><br>O*H*- Tyr132 H-bond
***R*-11b**	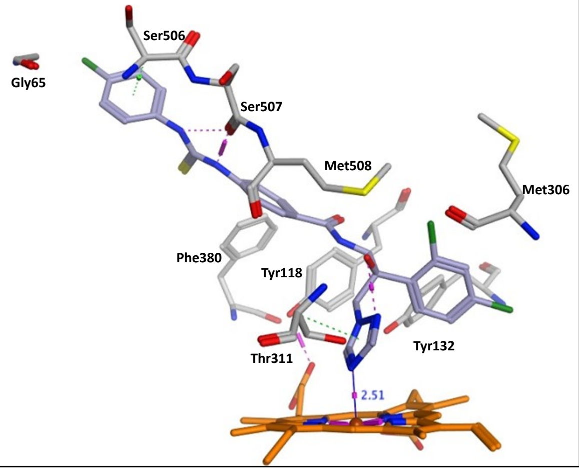	2.51	Thiourea 2 x NH – Ser506 and Ser507 H-bonding<br><br>Cl-*benzene* – Ser506 VdW<br><br>Triazole – Thr311 VdW<br><br>DiCl-benzene – Tyr132 π-π face-edge

***S*-11b**	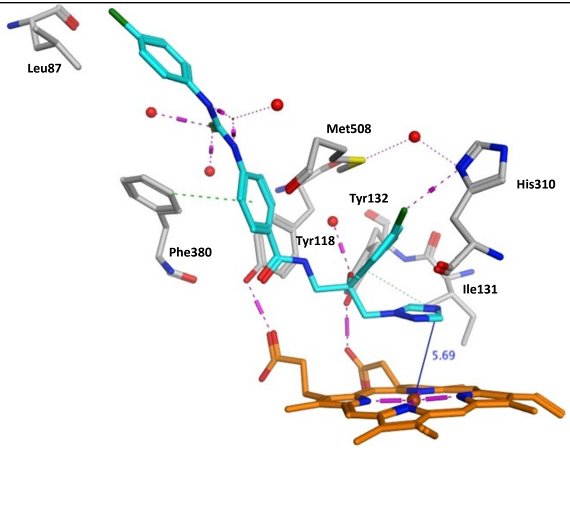	5.69*	Thiourea S and NH - H_2O bonding<br><br>OH – Tyr132 H-bond<br><br>Triazole – Tyr132 VdW<br><br>2,4-diCl benzene 4-Cl group bonding with His310<br><br>Central benzene ring – Phe380 π-π stacking
***R*-12a**	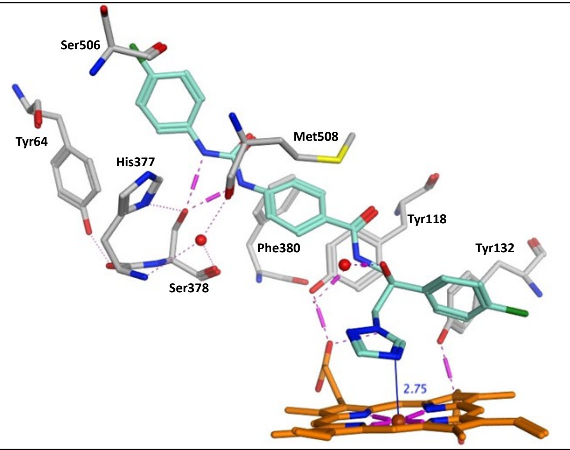	2.75*	Urea 2 x NH – Ser378 and His377 direct H-bonding, Met508 H_2O mediated H-bonding<br><br>O*H*-Tyr132
***S*-12a**	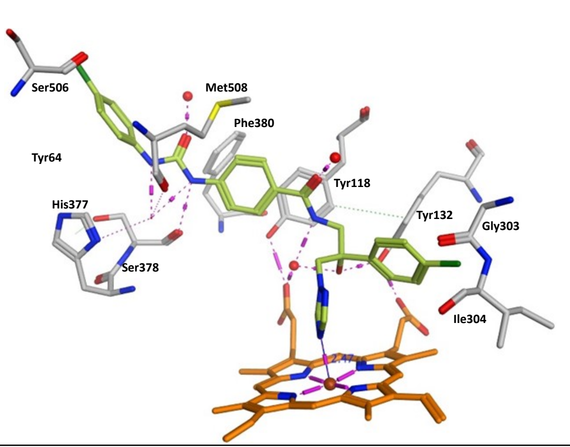	2.47	Urea 2 x NH – Ser378 and His377 direct H-bonding<br><br>O*H*- Tyr132 H-bond<br><br>Amide NH – water mediated with haem<br><br>Urea and amide C=*O* bond with H_2O

***R*-12b**	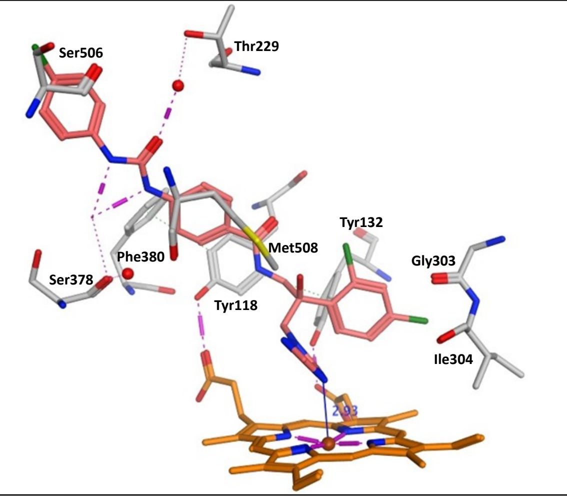	2.93*	Urea 2 x NH – Ser378 direct H-bonding<br><br>Urea C=*O* - H_2O mediated H-bonding Thr229
***S*-12b**	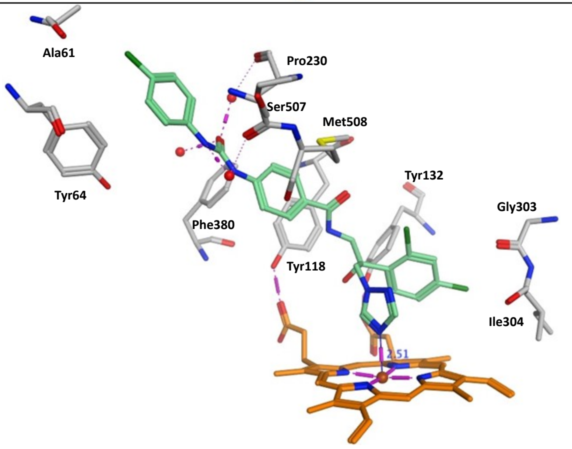	2.51	Urea 2 x NH – H_2O mediated with Ser507<br><br>Urea C=*O* - H_2O mediated H-bonding Pro230<br><br>DiCl-benzene – Tyr132 π-π face-edge<br><br>Central benzene ring – Phe380 π-π stacking<br><br>Cl-benzene – Tyr64 π-π face-edge
***R*-13a**	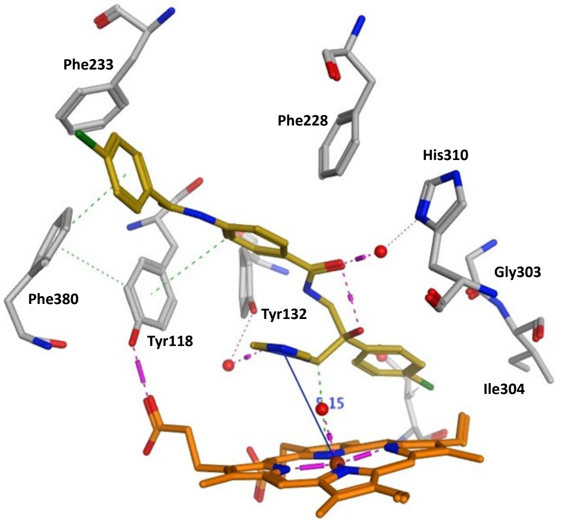	No interaction	Amide C*O*NH – His310 H_2O mediated H-bond<br><br>Triazole N – Tyr132 H_2O mediated H-bond<br><br>Amide Cl-*benzene* – Phe380 and Phe233 π-π stacking<br><br>Central *benzene* – Tyr118 and Phe228 π-π stacking

<table>
<tr>
<td>S-13a</td>
<td>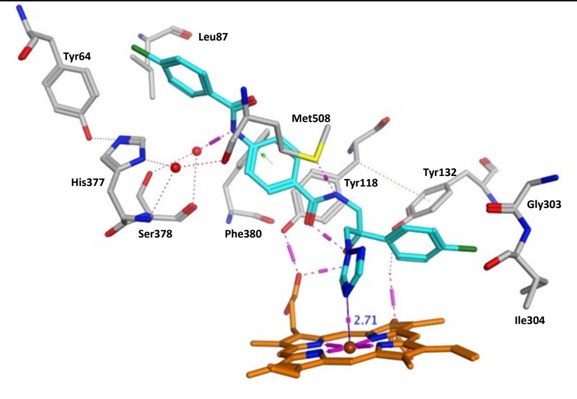
</td>
<td>2.71</td>
<td>Central amide NH – Met508 H-bond

End amide NH – Ser378 H_2O mediated H-bond

Central benzene – Phe380 π-π stacking

OH- haem H_2O mediated H-bond</td>
</tr>
<tr>
<td>R-13b</td>
<td>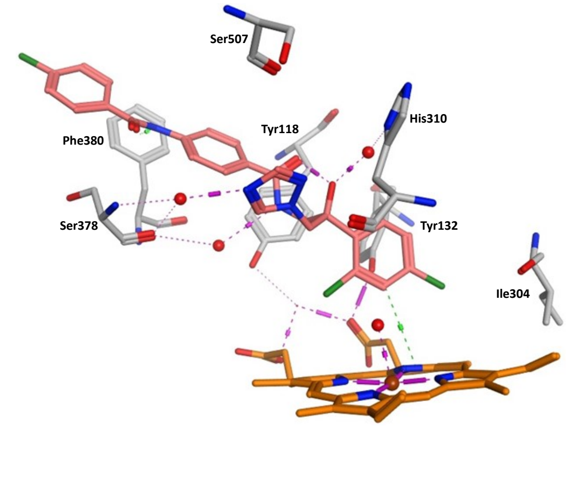
</td>
<td>No interaction</td>
<td>Triazole and central amide NH – Ser378 H_2O mediated H-bond

OH-His310 H_2O mediated H-bond

DiCl-benzene - haem VdW and Tyr132 π-π stacking

End amide NH – Phe380 cation-aryl bond</td>
</tr>
<tr>
<td>S-13b</td>
<td>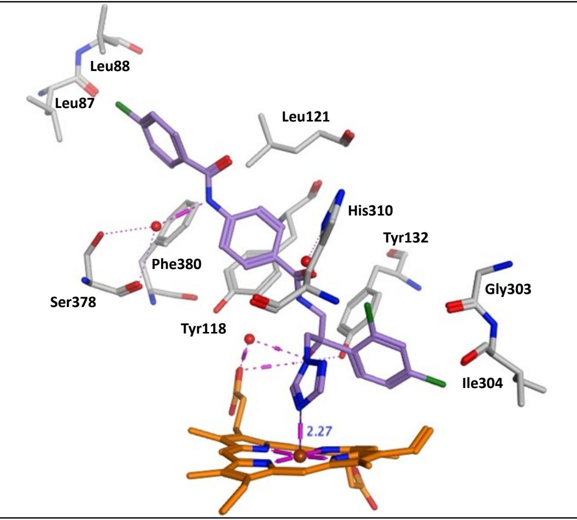
</td>
<td>2.27</td>
<td>Central amide NH – His310 H_2O mediated H-bond

End amide NH – Ser378 H_2O mediated H-bond

OH- haem and Tyr132 H_2O mediated H-bond</td>
</tr>
<tr>
<td>R-14a</td>
<td>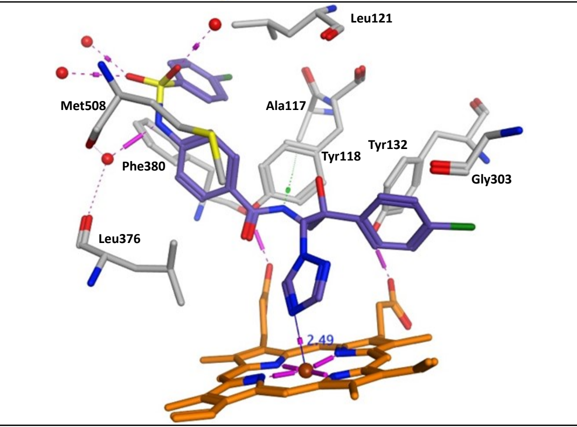
</td>
<td>2.49</td>
<td>SO_2NH – H_2O mediated H-bonding with Leu376 and Met508

π-π stacking Phe380 and Tyr132 and Cl-benzene rings</td>
</tr>
</table>

***S*-14a**	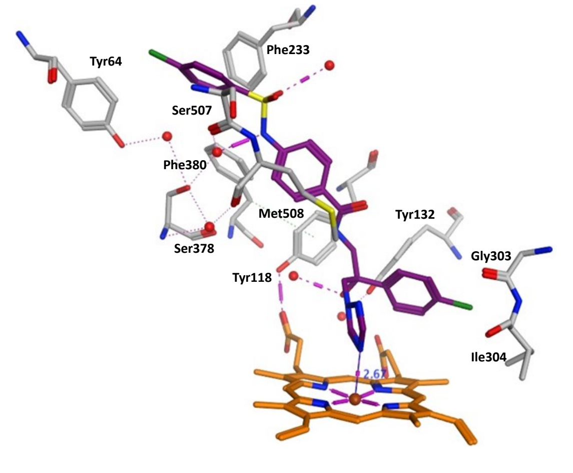	2.67	Sulfonamide N*H* – Ser378 and Ser507 H_2O mediated H-bond<br><br>O*H*- Tyr132 H-bond<br><br>S*O*$_2$NH bond with H_2O<br><br>Amide Cl-b*enzene* – Tyr132 π-π face edge
***R*-14b**	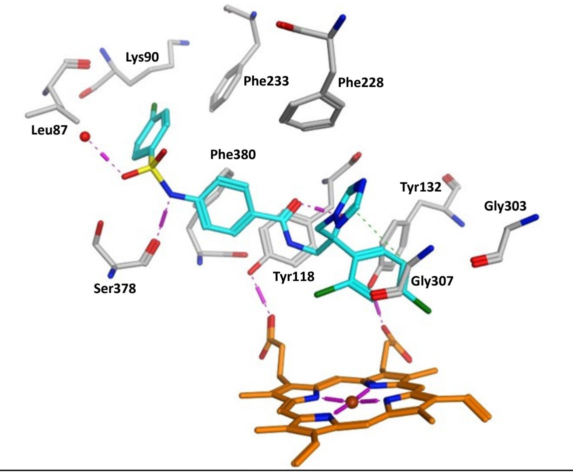	No interaction	Sulfonamide N*H* – Ser378 H-bond<br><br>S*O*$_2$NH bond with H_2O<br><br>Central *benzene* – Phe380 π-π face edge<br><br>Triazole – Phe228 π-π stacking<br><br>Triazole – Gly307 VdW
***S*-14b**	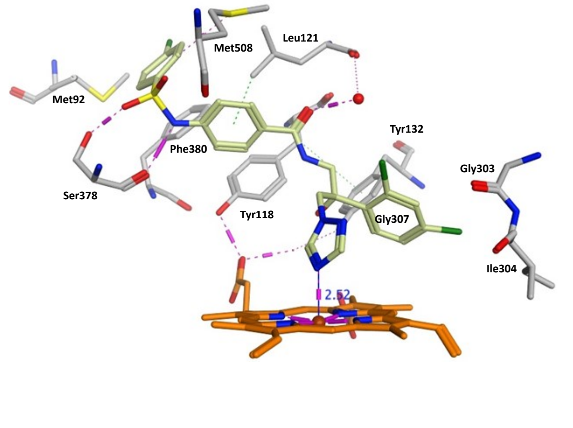	2.52	S*O*$_2$N*H* – Ser378 2 x H-bond<br><br>C*O*NH – Leu121 H_2O mediated H-bond<br><br>Cl-*benzene* – Met508<br><br>Central *benzene* – Tyr118 π-π face edge and Leu121 VdW

*Not perpendicular

Figure S6. 3D images illustrating binding position, haem Fe^{3+}-triazole binding distance and key binding interactions of protein-ligand interactions complexes of final frame after 200 ns MD simulation for enantiomers of **6a**, **6b**, **7a**, **7b**, **11a, 11b, 12a, 12b, 13a, 13b, 14a** and **14b** using wild type CaCYP51.

Procedures and characterisation of synthesised compounds:

General procedure for the preparation of triazole derivatives (2).

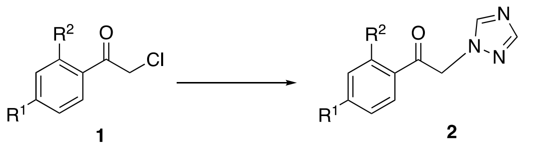

To a cooled solution of acetophenone derivatives (**1**) (15.87 mmol) in acetone (75 mL) was added 1,2,4-triazole (31.74 mmol) and K_2CO_3 (19.04 mmol). The reaction was stirred vigorously at 0 °C for 30 min then at room temperature overnight. The reaction mixture was filtered to remove inorganics (KCl) and the filtrate concentrated under reduced pressure. The residue obtained was extracted between EtOAc (100 mL) and washed with H_2O (3 x 50 mL), the combined aqueous extracts were back extracted with EtOAc (50 mL), then the combined organic layers dried (MgSO4) and concentrated under reduced pressure. The deep yellow residue was triturated with Et_2O to remove remaining acetophenone, then the yellow solid recrystallised from EtOH or purified by petroleum ether – EtOAc gradient column chromatography.

***1-(4-Chlorophenyl)-2-(1-H-1,2,4-triazol-1-yl)ethan-1-one* (2a, R^1 = Cl, R^2 = H).** Prepared from 2',4'-dichloroacetophenone (**1a**, R^1 = 4-Cl) (3.0 g, 15.87 mmol). Product obtained as a white crystalline solid, yield 1.79 g (51%). M.p. 148-150 °C (149-150 °C lit [1]). TLC (petroleum ether-EtOAc 1:2 v/v), R*f* = 0.35. ^{1}H NMR (DMSO-*d*$_6$): *δ* 8.51 (s, 1H, triazole), 8.07 (d, *J* = 8.8 Hz, 2H, Ar), 8.03 (s, 1H, triazole), 7.69 (d, *J* = 8.7 Hz, 2H, Ar), 6.00 (s, 2H, C*H*$_2$-triazole). ^{13}C NMR (DMSO-*d*$_6$): *δ* 192.22 (C, C=O), 151.79 (CH, triazole), 146.07 (CH, triazole), 139.55 (C, *C*-Cl), 133.37 (C, Ar), 130.52 (2 x CH, Ar), 129.59 (2 x CH, Ar), 55.68 (*C*H$_2$-triazole).

1-(2,4-Dichlorophenyl)-2-(1H-1,2,4-triazol-1-yl)ethan-1-one (*2b, R^1 = Cl, R^2 = Cl*). Prepared from 2,2',4'-trichloroacetophenone (**1b**, R^1= 2,4-di-Cl) (1.00 g, 4.48 mmoL). Product obtained as a pale-yellow solid, yield 0.85 g (74%). M.p. 100-102 °C (115-116 °C lit [2]). TLC (petroleum ether-EtOAc 1:2 v/v), R*f* = 0.21. ^{1}H NMR (DMSO-*d*$_6$): *δ* 8.54 (s, 1H, triazole), 8.03 (s, 1H, triazole), 7.96 (d, *J* = 8.4 Hz, 1H, Ar), 7.82 (d, *J* = 2.0 Hz, 1H, Ar), 7.65 (dd, *J* = 2.1, 8.4 Hz, 1H, Ar), 5.85 (s, 2H, C*H*$_2$-triazole). ^{13}C NMR (DMSO-*d*$_6$): *δ* 193.90 (C, C=O), 152.00 (CH, triazole), 146.04 (CH, triazole), 137.85 (C, Ar), 134.04 (C, *C*-Cl), 132.51 (C, *C*-Cl), 131.86 (CH, Ar), 131.00 (CH, Ar), 128.16 (CH, Ar), 57.59 (*C*H$_2$-triazole).

General procedure for the formation of the epoxide (3).

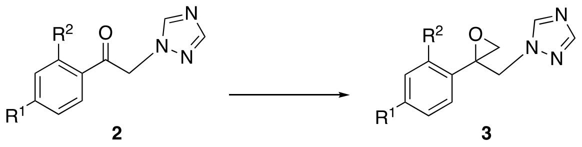

To a solution of 1-(arylphenyl)-2-(1*H*-1,2,4-triazol-1-yl)ethanone (**2**) (5 mmoL) in toluene (11 mL/mmoL) was added trimethylsulfoxonium iodide (TMSOI) (10 mmoL) followed by 20% aqueous NaOH (18.9 mmoL) and the reaction heated at 60 °C for 6 h then rt o/n. Upon completion, the reaction was diluted with H_2O (30 mL) and EtOAc (30 mL). The aqueous layer was extracted with EtOAc (3 x 20 mL), then the combined organic extracts washed with H_2O (2 x 30

mL), sat. aq. NaCl (20 mL), dried ($MgSO_4$) and concentrated to give the epoxide which was used in the next step without further purification.

***1-((2-(4-Chlorophenyl)oxiran-2-yl)methyl)-1H-1,2,4-triazole* (3a, R^1 = Cl, R^2 = H).** Prepared from 1-(4-chlorophenyl)-2-(1-*H*-1,2,4-triazol-1-yl)ethan-1-one (**2c**, R^1 = 4-Cl) (1.25 g, 5.66 mmoL). Product obtained as a light-yellow oil, which became dark orange on standing, yield 1.33 g (100 %). TLC (petroleum ether – EtOAc 1:2 v/v), R*f* = 0.28. ^{1}H NMR (DMSO-*d₆*): δ 8.39 (s, 1H, triazole), 7.91 (s, 1H, triazole), 7.40 (s, 4H, Ar), 5.06 (d, *J* = 15.0 Hz, 1H, C*Ha*Hb-triazole), 4.64 (d, *J* = 15.0 Hz, 1H, CHa*Hb*-triazole), 3.04 (d, *J* = 4.9 Hz, 1H, OC*Ha*Hb), 2.87 (d, *J* = 4.9 Hz, 1H, OCHa*Hb*). ^{13}C NMR (DMSO-d_6): δ 151.79 (CH, triazole), 145.40 (CH, triazole), 136.28 (C, Ar), 131.19 (C, Ar), 128.78 (2 x CH, Ar), 128.56 (2 x CH, Ar), 58.72 (*C*-epoxide), 53.99 (CH_2-triazole), 52.82 (CH_2-O).

***1-((2-(2,4-Dichlorophenyl)oxiran-2-yl)methyl)-1H-1,2,4-triazole* (3b, R^1 = Cl, R^2 = Cl).** Prepared from 1-(2,4-dichlorophenyl)-2-(1*H*-1,2,4-triazol-1-yl)ethan-1-one (**2b**, R^1= 2,4-diCl) (1.88 g, 7.32 mmoL). Product obtained as a pale yellow to orange oil, yield 1.96 g (99 %). TLC (petroleum ether – EtOAc 1:2 v/v), R*f* = 0.38. ^{1}H NMR (DMSO-d_6): δ 8.40 (s, 1H, triazole), 7.91 (s, 1H, triazole), 7.67 (d, *J* = 2.1 Hz, 1H, Ar), 7.36 (dd, *J* = 2.1, 8.3 Hz, 1H, Ar), 7.12 (d, *J* = 8.4 Hz, 1H, Ar), 4.87 (d, *J* = 15.0 Hz, 1H, CH_aH_b-triazole), 4.55 (d, *J* = 14.9 Hz, 1H, CH_aH_b-triazole), 3.13 (d, *J* = 4.8 Hz, 1H, $COCH_aH_b$), 2.94 (d, *J* = 4.75 Hz, 1H, $COCH_aH_b$). ^{13}C NMR (DMSO-d_6): *δ* 151.92 (CH, triazole), 145.50 (CH, triazole), 134.44 (C, Ar), 134.23 (C, *C*-Cl), 133.58 (C, *C*-Cl), 131.41 (CH, Ar), 129.15 (CH, Ar), 127.80 (CH, Ar), 58.95 (*C*-epoxide), 52.91 (CH_2-triazole), 52.24 (CH_2-O).

General procedure for the preparation of the azide derivatives (4).

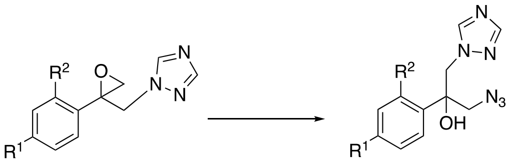

3 4

To a solution of epoxide derivative (**3**) (1.0 meq) in dry DMF (2.7 mL/mmoL) was added NaN_3 (1.95 meq) and NH_4Cl (1.2 meq) and the reaction heated at 60 °C for 2 h then rt o/n. After cooling to room temperature sat. aq. $NaHCO_3$ (50 mL/meq) was added and the reaction extracted with EtOAc (50 mL). The aqueous layer was back extracted with EtOAc (25 mL), then the combined organic layers washed with H_2O (25 mL), sat. aq. NaCl (25 mL), dried ($MgSO_4$) and concentrated under reduced pressure. The crude product was purified by gradient column chromatography.

***1-Azido-2-(4-chlorophenyl)-3-(1H-1,2,4-triazol-1-yl)propan-2-ol* (4a, R^1 = Cl, R^2 = H)).** Prepared from 1-((2-(4-chlorophenyl)oxiran-2-yl)methyl)-1*H*-1,2,4-triazole (**3a**, R^1= 4-Cl) (1.33 g, 5.64 mmoL). Product obtained as a thick yellow syrup after purification by gradient column chromatography (petroleum ether – EtOAc to 40:60 v/v), yield 1.02 g (65 %). TLC (petroleum ether – EtOAc 1:2 v/v), R*f* = 0.45. ^{1}H NMR (DMSO-d_6): *δ* 8.22 (s, 1H, triazole), 7.85 (s, 1H, triazole), 7.43 (d, *J* = 8.8 Hz, 2H, Ar), 7.37 (d, *J* = 8.8 Hz, 2H, Ar), 6.14 (s, 1H, O*H*), 4.54 (dd, *J* = 14.3, 22.0 Hz, 2H, CH_2-triazole), 3.65 (dd, *J* = 12.9, 22.6 Hz, 2H, CH_2-N_3). ^{13}C NMR (DMSO-d_6): *δ* 151.27 (CH, triazole), 145.59 (CH, triazole), 141.06 (C, Ar), 132.54 (C, *C*-Cl), 128.32 (2 x CH, Ar), 128.24 (2 x CH, Ar), 75.95 (C, *C*-OH), 57.88 (CH_2-triazole), 56.35 (CH_2-N_3). HRMS (ESI), *m/z*. calcd for $C_{11}H_{11}ClN_6O$ ([M + H]$^+$), 279.0761; found, 279.0761. HPLC (Method B): 98.99%, R_t = 4.94 min.

***1-Azido-2-(2,4-dichlorophenyl)-3-(1H-1,2,4-triazol-1-yl)propan-2-ol* (4b, R^1 = Cl, R^2 = Cl).** Prepared from 1-((2-(2,4-dichlorophenyl)oxiran-2-yl)methyl)-1*H*-1,2,4-triazole (**3b**, R^1= 2,4-diCl) (1.94 g, 7.17 mmoL). Product obtained as a

thick yellow syrup after purification by gradient column chromatography (petroleum ether – EtOAc to 40:60 v/v), yield 1.48 g (66 %). TLC (Petroleum ether – EtOAc 1:2 v/v), R*f* = 0.31. ^{1}H NMR (DMSO-d_6): *δ* 8.32 (s, 1H, triazole), 7.79 (s, 1H, triazole), 7.59 (t, *J* = 2.4 Hz, 2H, Ar), 7.39 (dd, *J* = 2.2, 8.7 Hz, 1H, Ar), 6.47 (s, 1H, O*H*), 4.85 (d, *J* = 14.5 Hz, 1H, C*Ha*Hb-triazole), 4.69 (d, *J* = 14.5 Hz, 1H, CHa*Hb*-triazole), 4.10 (d, *J* = 13.2 Hz, 1H, C*Ha*Hb-N_3), 3.74 (d, *J* = 13.2 Hz, 1H, CHa*Hb*-N_3). ^{13}C NMR (DMSO-d_6): *δ* 151.27 (CH, triazole), 145.66 (CH, triazole), 137.34 (C, Ar), 133.72 (C, *C*-Cl), 131.73 (CH, Ar), 131.54 (C, *C*-Cl), 130.46 (CH, Ar), 127.61 (CH, Ar), 76.78 (C, *C*-OH), 55.75 (*C*H_2- triazole), 53.98 (*C*H_2-N_3). HRMS (ESI), *m/z*. calcd for $C_{11}H_{10}Cl_2N_6O$ ([M + H]$^+$), 313.0371; found, 313.0373. HPLC (Method B): 99.27%, R_t = 5.03 min.

General procedure for the preparation of the free amines (5).

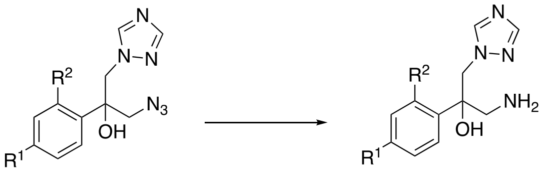

To a solution of azide derivative (**4**) (1.0 meq) in dry THF (5 mL) was added triphenylphosphine (1.15 meq) and the reaction stirred at room temperature for 1 h. H_2O (11.0 meq) was added and the reaction heated at 60 °C for 4 h. The reaction was concentrated under reduced pressure and to the resulting residue 2M aqueous HCl (20 mL) was added and the reaction stirred at room temperature for 20 min before extracting with CH_2Cl_2 (4 x 20 mL) to remove excess Ph_3P and triphenylphosphine oxide by-product. To the aqueous layer was added 1 M aqueous NaOH until basic pH; the free amine was then extracted with EtOAc (2 x 50 mL). The organic layers were combined, dried ($MgSO_4$) and concentrated under reduced pressure.

***1-Amino-2-(4-chlorophenyl)-3-(1H-1,2,4-triazol-1-yl)propan-2-ol* (5a, R^1 = Cl, R^2 = H)).** Prepared from 1-azido-2-(4-chlorophenyl)-3-(1*H*-1,2,4-triazol-1-yl)propan-2-ol (**4a**, R^1= 4-Cl) (0.514 g, 1.84 mmoL). Product obtained as a white solid on standing overnight, yield 0.36 g (77%). M.p. 94-96 °C. TLC (CH_2Cl_2-MeOH 9:1 v/v), R*f* = 0.4. ^{1}H NMR (DMSO-d_6): δ 8.20 (s, 1H, triaz), 7.82 (s, 1H, triaz), 7.40 (d, *J* = 8.8 Hz, 2H, Ar), 7.33 (d, *J* = 8.8 Hz, 2H, Ar), 5.54 (brs, 2H, N*H₂* partially exchanged), 4.50 (dd, *J* = 14.3, 19.3 Hz, 2H, C*H₂*-triazole), 2.81 (s, 2H, C*H₂*-NH_2). (O*H* exchanged so not observed). ^{13}C NMR (DMSO-d_6): δ 150.93 (CH, triazole), 145.30 (CH, triazole), 142.71 (C, Ar), 131.91 (C, *C*-Cl), 128.28 (2 x CH, Ar), 128.16 (2 x CH, Ar), 75.92 (C, *C*-OH), 56.51 (*C*H_2-triazole), 50.00 (*C*H_2-NH_2). HRMS (ESI), *m/z*. calcd for $C_{11}H_{13}ClN_4O$ ([M + H]$^+$), 253.0856; found, 253.0855. HPLC (Method B): 97.62%, R_t = 4.85 min.

***1-Amino-2-(2,4-dichlorophenyl)-3-(1H-1,2,4-triazol-1-yl)propan-2-ol* (5b, R^1 = Cl, R^2 = Cl).** Prepared 1-azido-2-(2,4-dichlorophenyl)-3-(1*H*-1,2,4-triazol-1-yl)propan-2-ol (**4b**, R^1= 2,4-diCl) (1.45 g, 4.62 mmoL). Product obtained as a white solid, yield 0.80 g (60 %). M.p. 70-72 °C. TLC (CH_2Cl_2-MeOH 9:1 v/v), R*f* = 0.48. ^{1}H NMR (DMSO-d_6): δ 8.29 (s, 1H, triazole), 7.72 (s, 1H, triazole), 7.53 (t, *J* = 2.1 Hz, 1H, Ar), 7.52 (s, 1H, Ar), 7.30 (dd, *J* = 2.2, 8.6 Hz, 1H, Ar), 5.80 (brs, 1H, O*H*), 4.87 (d, *J* = 14.3 Hz, 1H, C*Ha*Hb-triazole), 4.50 (d, *J* = 14.4 Hz, 1H, CHa*Hb*-triazole), 3.21 (d, *J* = 13.5 Hz, 1H, C*Ha*Hb-NH_2), 3.07 (d, *J* = 13.5 Hz, 1H, CHa*Hb*-NH_2), 1.55 (brs, 2H, N*H₂*). ^{13}C NMR (DMSO-d_6): δ 150.88 (CH, triazole), 145.37 (CH, triazole), 139.27 (C, Ar), 132.97 (C, *C*-Cl), 131.76 (CH, Ar), 131.58 (C, *C*-Cl), 130.36 (CH, Ar), 127.28 (CH, Ar), 76.47 (C, *C*-OH), 54.36 (*C*H_2-triazole), 47.04 (*C*H_2-NH_2).). HRMS (ESI), *m/z*. calcd for $C_{11}H_{12}Cl_2N_4O$ ([M + H]$^+$), 287.0466; found, 287.0467. HPLC (Method B): 99.33%, R_t = 4.78 min.

General procedure for the preparation of the nitro intermediates derivatives (9).

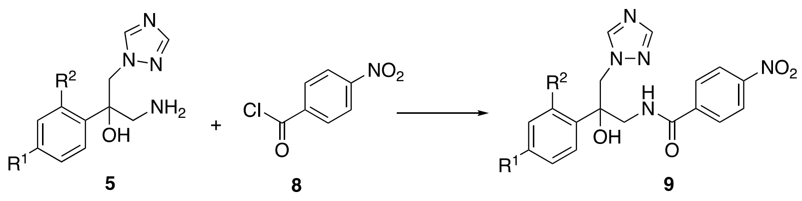

A solution of CH_2Cl_2 (7.5 mL) and saturated aqueous $NaHCO_3$ (15 mL) were stirred vigorously and chilled in an ice bath. 4-NitroBenzoyl chloride (**8**) (4.37 mmoL) was added, stirred until all the solid dissolved, followed by the free amine derivatives (**5**) (2.91 mmoL). Stirring was continued while warming to room temperature over a period of 2 h. The reaction mixture was evaporated, and the obtained residue diluted with EtOAc (15 mL), extracted with H_2O (5 mL), dried ($MgSO_4$) and the solvent evaporated under vacuum.

N-(2-(4-Chlorophenyl)-2-hydroxy-3-(1-H-1,2,4-triazol-1-yl)propyl)-4-nitrobenzamide **(9a, R^1 = Cl, R^2 = H).** Prepared from 1-amino-2-(4-chlorophenyl)-3-(1*H*-1,2,4-triazol-1-yl)propan-2-ol (**5a**, R^1= 4-Cl) (0.2 g, 0.79 mmoL). Product obtained as a white solid, which was purified by gradient column chromatography CH_2Cl_2-MeOH (97: 3 v/v), yield 0.24 g (74 %). M.p. 228-230 °C. TLC (CH_2Cl_2-MeOH 9.5:0.5 v/v), R*f* = 0.44. ^1H NMR (DMSO-*d*$_6$): *δ* 8.63 (t, *J* = 5.9 Hz, 1H, N*H*), 8.29 (d, *J* = 8.9 Hz, 2H, Ar), 8.26 (s, 1H, triazole), 7.96 (d, *J* = 8.9 Hz, 2H, Ar), 7.84 (s, 1H, triazole), 7.45 (d, *J* = 8.6 Hz, 2H, Ar), 7.32 (d, *J* = 8.7 Hz, 2H, Ar), 6.00 (s, 1H, O*H*), 4.63 (dd, *J* = 14.4, 20.8 Hz, 2H, CH_2-triazole), 3.91 (dd, *J* = 6.8, 13.9 Hz, 1H, C*Ha*HbNH), 3.64 (dd, *J* = 5.3, 13.9 Hz, 1H, CHa*Hb*NH). ^{13}C NMR (DMSO-*d*$_6$): δ 166.09 (C, C=O), 151.05 (CH, triazole), 149.50 (C, Ar), 145.47 (CH, triazole), 141.28 (C, Ar), 140.34 (C, Ar), 132.22 (C, *C*-Cl), 129.28 (2 x CH, Ar), 128.37 (2 x CH, Ar), 128.11 (2 x CH, Ar), 123.93 (2 x CH, Ar), 76.12 (C, *C*-OH), 56.92 (*C*H_2-triazole), 49.15 (*C*H_2-NH_2). HRMS (ESI), *m/z*. calcd for $C_{18}H_{16}ClN_5O_4$ ($[M + H]^+$), 402.0969; found, 402.0969. HPLC (Method B): 99.9%, R_t = 4.84 min.

N-(2-(2,4-dichlorophenyl)-2-hydroxy-3-(1H-1,2,4-triazol-1-yl)propyl)-4-nitrobenzamide **(9b, R^1 = Cl, R^2 = Cl)**. Prepared from 1-amino-2-(2,4-dichlorophenyl)-3-(1*H*-1,2,4-triazol-1-yl)propan-2-ol (**5b**, R^1= 2,4-diCl) (0.5 g, 1.74 mmoL). Product obtained as a white solid which was purified by gradient column chromatography CH_2Cl_2-MeOH (97.5: 2.5 to 97: 3 v/v), yield 0.42 g (55 %). M.p. 228-230 °C. TLC (CH_2Cl_2-MeOH 9.5:0.5 v/v), R*f* = 0.5. ^1H NMR (DMSO-*d*$_6$): δ 8.79 (t, *J* = 6.1 Hz, 1H, N*H*), 8.34 (s, 1H, triazole), 8.30 (d, *J* = 9.0 Hz, 2H, Ar), 7.99 (d, *J* = 8.7 Hz, 2H, Ar), 7.75 (s, 1H, triazole), 7.58 (s, 1H, Ar), 7.56 (d, *J* = 2.2 Hz, 1H, Ar), 7.29 (dd, *J* = 2.2, 8.6 Hz, 1H, Ar), 6.26 (brs, 1H, O*H*), 5.10 (d, *J* = 14.5 Hz, 1H, C*Ha*Hb-triazole), 4.71 (d, *J* = 14.5 Hz, 1H, CHa*Hb*-triazole), 4.07 (dd, *J* = 5.8, 14.0 Hz, 1H, C*Ha*HbNH), 4.00 (dd, *J* = 6.4, 14.0 Hz, 1H, CHa*Hb*NH). ^{13}C NMR (DMSO-*d*$_6$): δ 166.49 (C, C=O), 151.06 (CH, triazole), 149.55 (C, Ar), 145.46 (CH, triazole), 140.16 (C, Ar), 137.97 (C, Ar), 133.33 (C, *C*-Cl), 132.11 (C, *C*-Cl), 131.49 (CH, Ar), 130.46 (CH, Ar), 129.36 (2 x CH, Ar), 127.27 (CH, Ar), 123.93 (2 x CH, Ar), 76.74 (C, *C*-OH), 54.03 (*C*H_2-triazole), 45.81 (*C*H_2-NH_2). HRMS (ESI), *m/z*. calcd for $C_{18}H_{15}Cl_2N_5O_4$ ($[M + H]^+$), 436.0579; found, 436.0578. HPLC (Method B): 99.99%, R_t = 4.68 min.

General procedure for reduction of nitro derivatives to free amine derivatives (10).

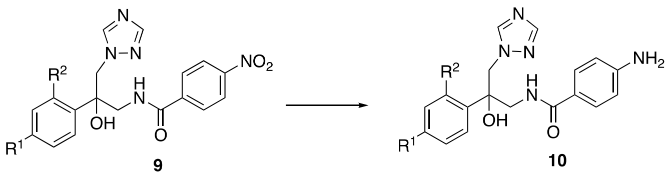

To a solution of *N*-(2-(arylphenyl)-2-hydroxy-3-(1*H*-1,2,4-triazol-1-yl)propyl)-4-nitrobenzamide derivative (**9**) (1.0 meq) in dry MeOH (15 mL) was added 10% Pd/C. Then, the reaction atmosphere was degassed, filled with hydrogen (using hydrogen balloon) and the mixture stirred at rt for 3 h. The suspension was filtered through a pad of celite and the solvent removed under reduce pressure. The crude product was purified by gradient column chromatography CH_2Cl_2-MeOH (97: 3 v/v).

4-Amino-N-(2-(4-chlorophenyl)-2-hydroxy-3-(1H-1,2,4-triazol-1-yl)propyl)benzamide **(10a, R^1 = Cl, R^2 = H).** Prepared from *N*-(2-(4-chlorophenyl)-2-hydroxy-3-(1*H*-1,2,4-triazol-1-yl)propyl)-4-nitrobenzamide (**9a**, R^1= 4-Cl) (0.2 g, 0.5 mmoL). Product obtained as an off-white wax, yield: 0.13 g (71 %). TLC (CH_2Cl_2-MeOH 9.5:0.5 v/v), R*f* = 0.35. ^{1}H NMR (DMSO-d_6): *δ* 8.25 (s, 1H, triazole), 8.00 (t, *J* = 5.9 Hz, 1H, N*H*), 7.82 (s, 1H, triazole), 7.48 (d, *J* = 8.7 Hz, 2H, Ar), 7.42 (d, *J* = 8.8 Hz, 2H, Ar), 7.31 (d, *J* = 8.8 Hz, 2H, Ar), 6.51 (d, *J* = 8.8 Hz, 2H, Ar), 6.40 (s, 1H, O*H*), 4.53 (dd, *J* = 14.3, 20.3 Hz, 2H, C*H₂*-triazole), 3.79 (dd, *J* = 6.6, 14.1 Hz, 1H, C*Ha*HbNH), 3.60 (dd, *J* = 5.1, 14.1 Hz, 1H, CHa*Hb*NH). ^{13}C NMR (DMSO-d_6): *δ* 168.38 (C, C=O), 152.50 (C, Ar), 150.97 (CH, triazole), 145.45 (CH, triazole), 141.73 (C, Ar), 132.07 (C, *C*-Cl), 129.41 (2 x CH, Ar), 128.42 (2 x CH, Ar), 128.09 (2 x CH, Ar), 120.45 (C, Ar), 112.92 (2 x CH, Ar), 76.54 (C, *C*-OH), 57.33 (*C*H_2-triazole), 48.39 (*C*H_2-NH). HRMS (ESI), *m/z*. calcd for $C_{18}H_{18}ClN_5O_2$ ([M + Na]$^+$), 394.1047; found, 394.1047. HPLC (Method B): 99.81%, R_t = 4.695 min.

4-Amino-N-(2-(2,4-dichlorophenyl)-2-hydroxy-3-(1H-1,2,4-triazol-1-yl)propyl)benzamide **(10b, R^1 = Cl, R^2 = Cl).** Prepared from *N*-(2-(2,4-dichlorophenyl)-2-hydroxy-3-(1*H*-1,2,4-triazol-1-yl)propyl)-4-nitrobenzamide (**9b**, R^1= 2,4-diCl) (0.38 g, 0.86 mmoL). Product obtained as a pale-yellow wax, yield 0.35 g (100 %). TLC (CH_2Cl_2-MeOH 9.5:0.5 v/v), R*f* = 0.36. ^{1}H NMR (DMSO-d_6): *δ* 8.34 (s, 1H, triazole), 8.21 (t, *J* = 5.9 Hz, 1H, N*H*), 7.73 (s, 1H, triazole), 7.58 (d, *J* = 8.7 Hz, 1H, Ar), 7.55 (d, *J* = 2.2 Hz, 1H, Ar), 7.51 (d, *J* = 8.7 Hz, 2H, Ar), 7.28 (dd, *J* = 2.3, 8.7 Hz, 1H, Ar), 6.87 (s, 1H, O*H*), 6.51 (d, *J* = 8.7 Hz, 2H, Ar), 5.71 (brs, 2H, N*H₂*), 5.00 (d, *J* = 14.3 Hz, 1H, C*Ha*Hb-triazole), 4.65 (d, *J* = 14.3 Hz, 1H, CHa*Hb*-triazole), 3.94 (d, *J* = 5.3 Hz, 2H, C*H₂*-NH). ^{13}C NMR (DMSO-d_6): *δ* 169.23 (C, C=O), 152.67 (C, Ar), 150.96 (CH, triazole), 145.61 (CH, triazole), 138.48 (C, Ar), 133.22 (C, *C*-Cl), 131.96 (C, *C*-Cl), 131.67 (CH, Ar), 130.37 (CH, Ar), 129.59 (2 x CH, Ar), 127.29 (CH, Ar), 120.01 (C, Ar), 112.90 (2 x CH, Ar), 77.21 (C, *C*-OH), 54.33 (*C*H_2-triazole), 46.39 (*C*H_2-NH). HRMS (ESI), *m/z*. calcd for $C_{18}H_{17}Cl_2N_5O_2$ ([M + Na]$^+$), 428.0657; found, 428.0660. HPLC (Method B): 99.98%, R_t = 4.68 min.

Figure S7. ^{1}H NMR and ^{13}C NMR and HPLC trace of **6a**

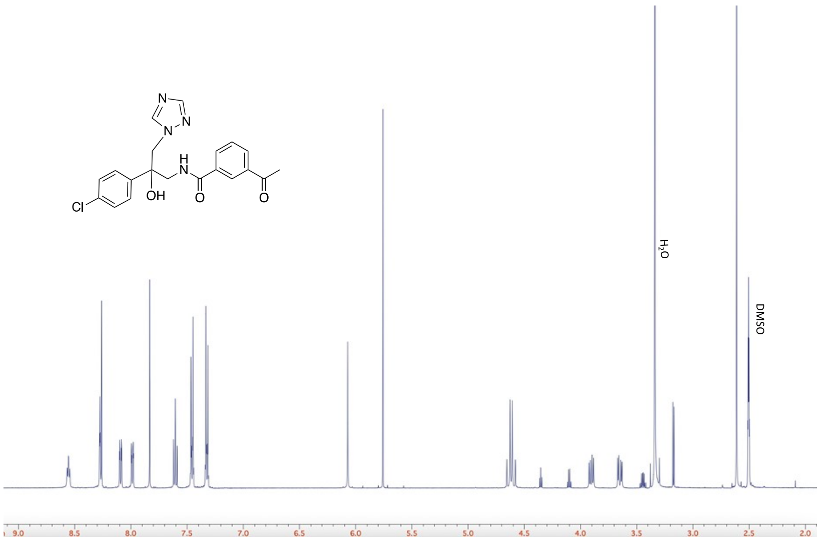

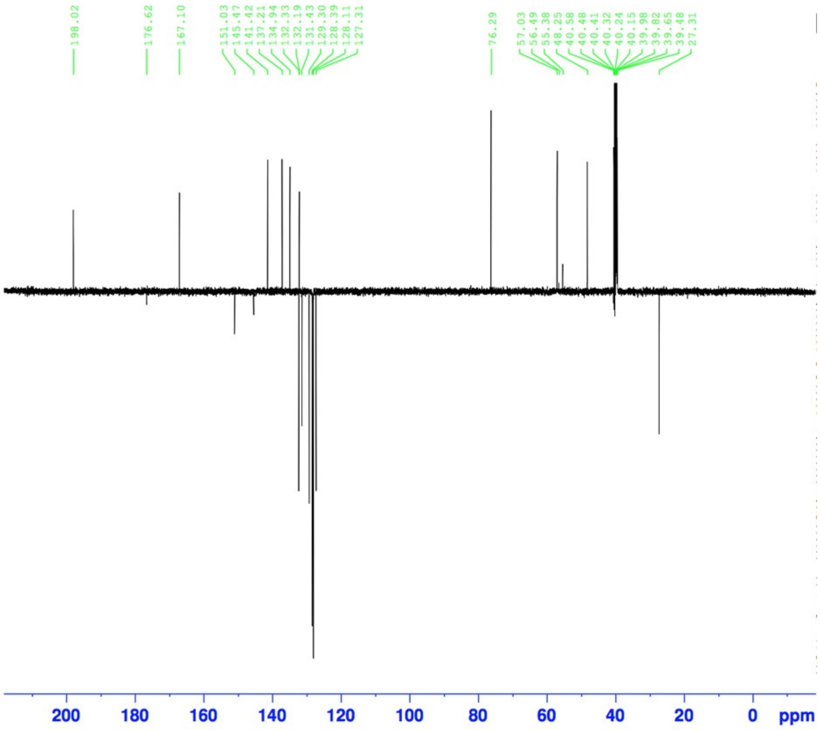

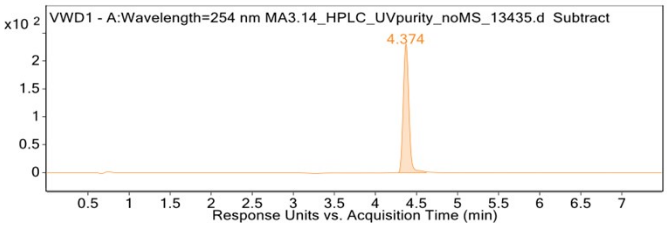

Figure: Base peak or HPLC chromatogram (indicated in left hand corner)

User Chromatogram Peak List

RT (min)	Area	Area %	Area Sum (%)	Symmetry	Width (min)
4.37	1103.59	100.00	100.00	1.15	0.340

Figure S8. ^{1}H NMR and ^{13}C NMR and HPLC trace of **6b**

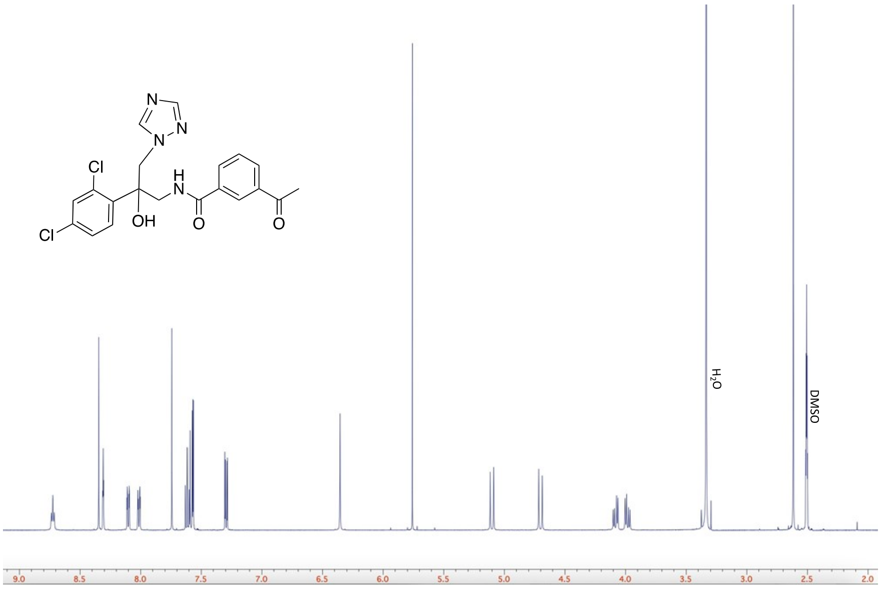

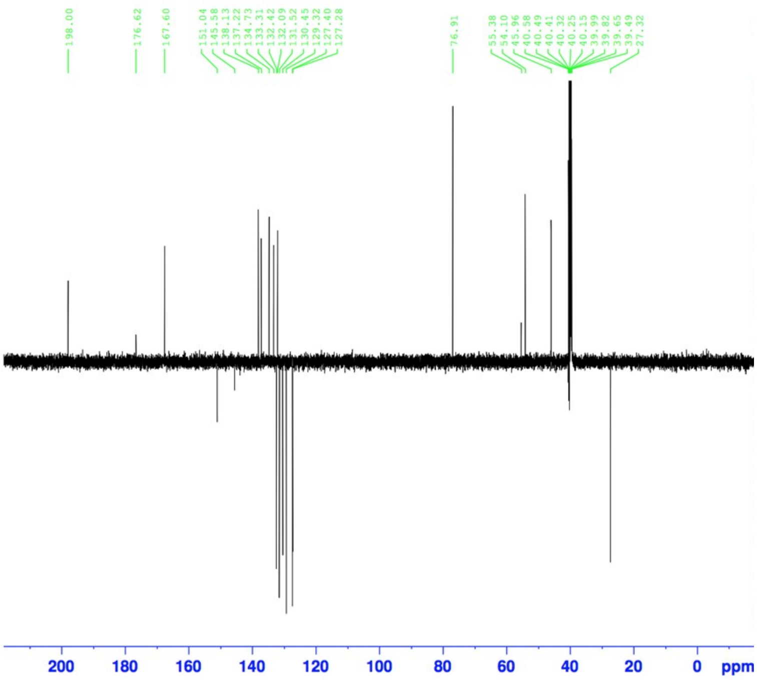

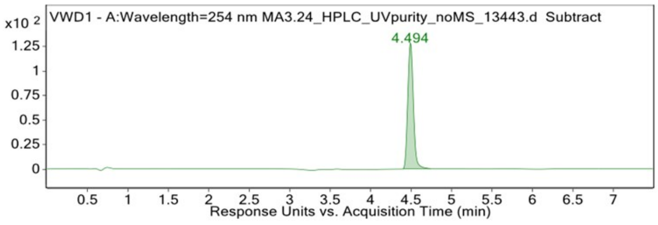

Figure: Base peak or HPLC chromatogram (indicated in left hand corner)

User Chromatogram Peak List

RT (min)	Area	Area %	Area Sum (%)	Symmetry	Width (min)
4.49	618.28	100.00	100.00	1.21	0.336

Figure S9. ^{1}H NMR and ^{13}C NMR and HPLC trace of **7a**

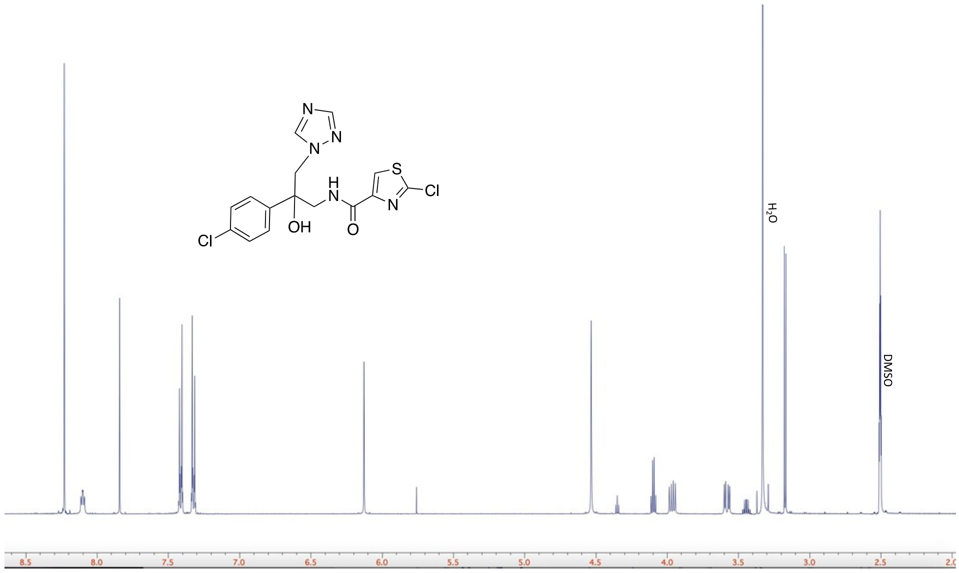

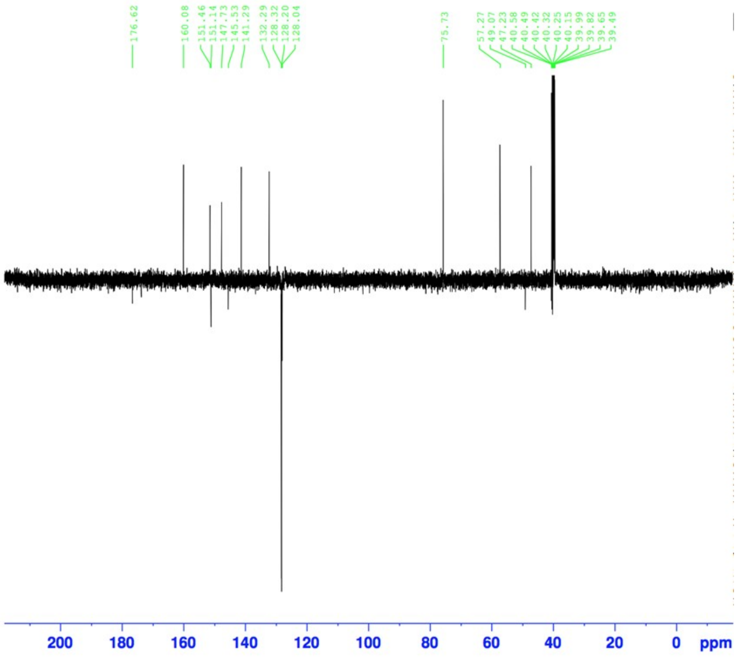

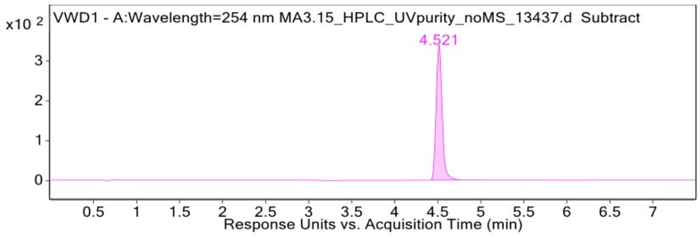

Figure: Base peak or HPLC chromatogram (indicated in left hand corner)

User Chromatogram Peak List

RT (min)	Area	Area %	Area Sum (%)	Symmetry	Width (min)
4.52	1661.68	100.00	100.00	1.07	0.346

Figure S10. ^{1}H NMR and ^{13}C NMR and HPLC trace of **7b**

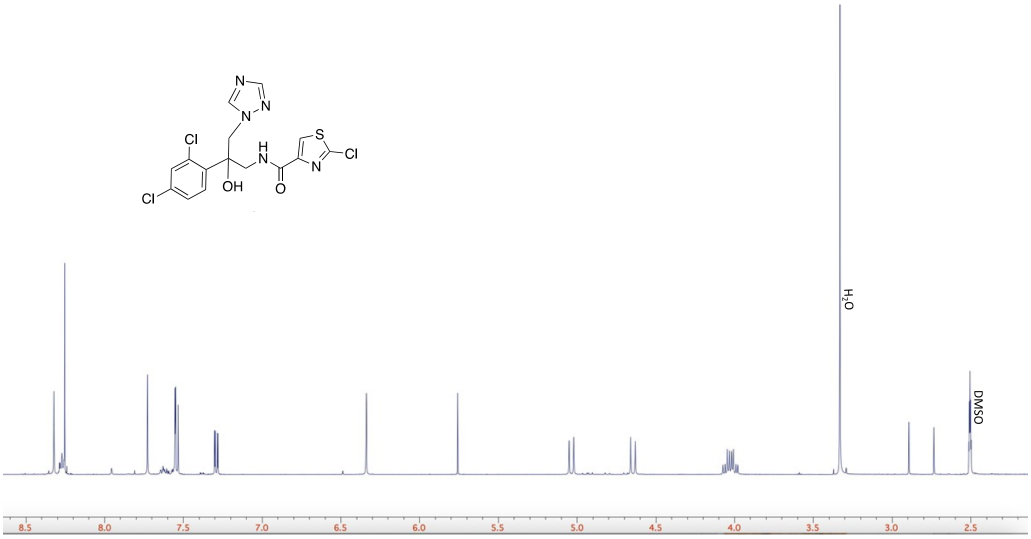

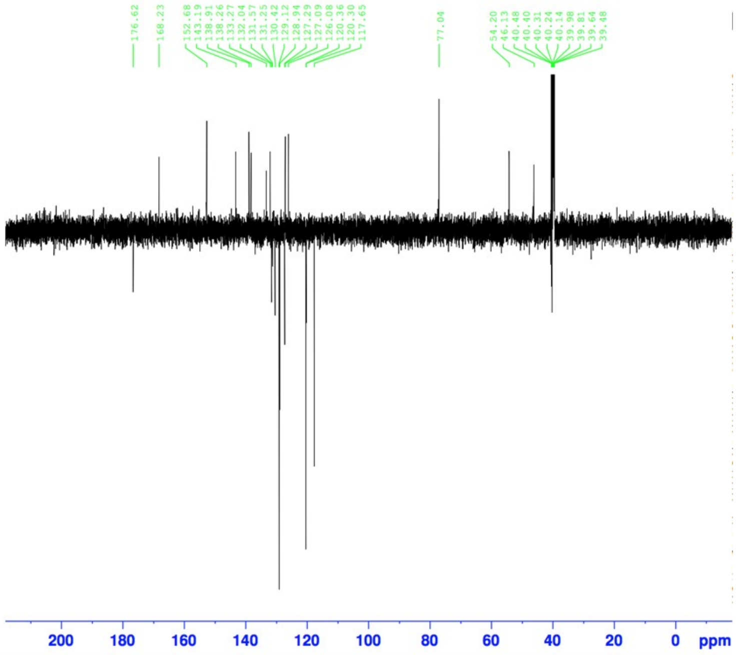

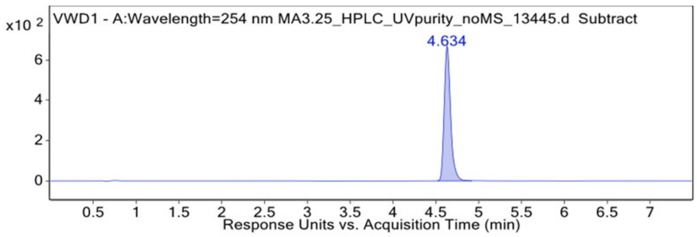

Figure: Base peak or HPLC chromatogram (indicated in left hand corner)

User Chromatogram Peak List

RT (min)	Area	Area %	Area Sum (%)	Symmetry	Width (min)
4.63	3489.79	100.00	100.00	1.44	0.400

Figure S11. ^{1}H NMR and ^{13}C NMR and HPLC trace of **11a**

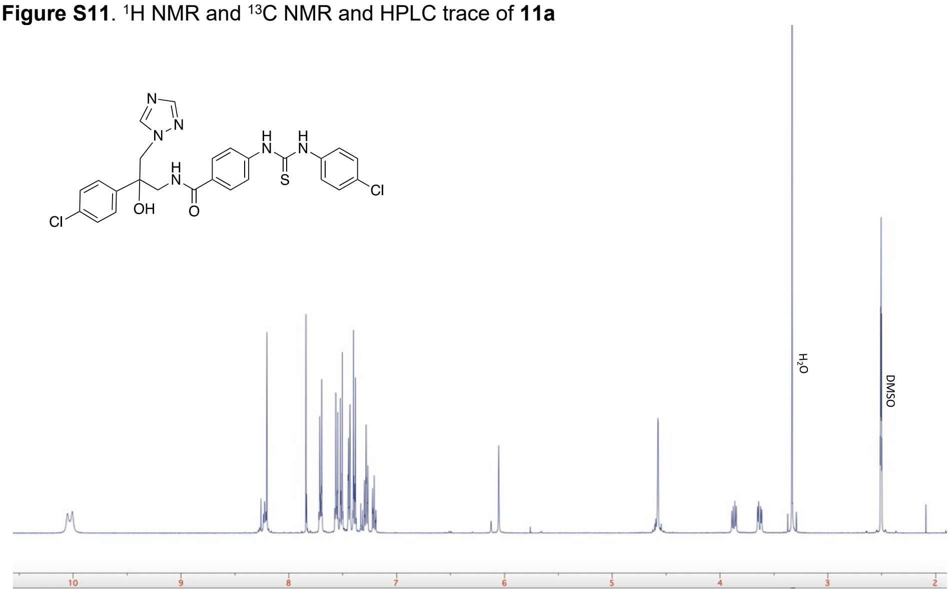

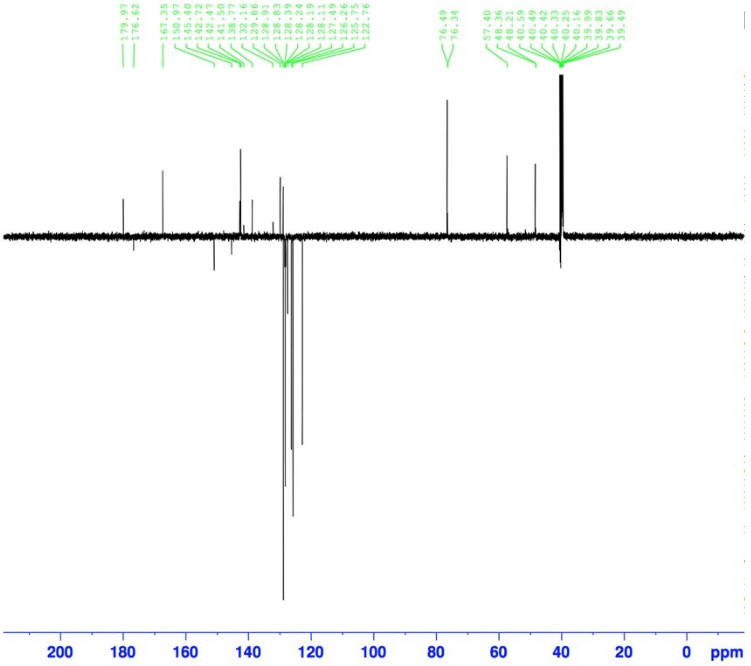

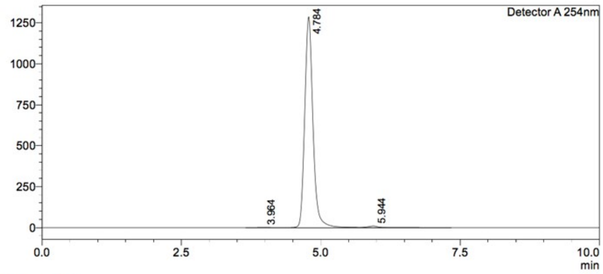

<Peak Table>

Detector A 254nm

Peak#	Ret. Time	Area	Height	Area%
1	3.964	5392	215	0.040
2	4.784	13271921	1285297	99.326
3	5.944	84727	8289	0.634
Total		13362040	1293801	100.000

Figure S12. ^{1}H NMR and ^{13}C NMR of **11b**

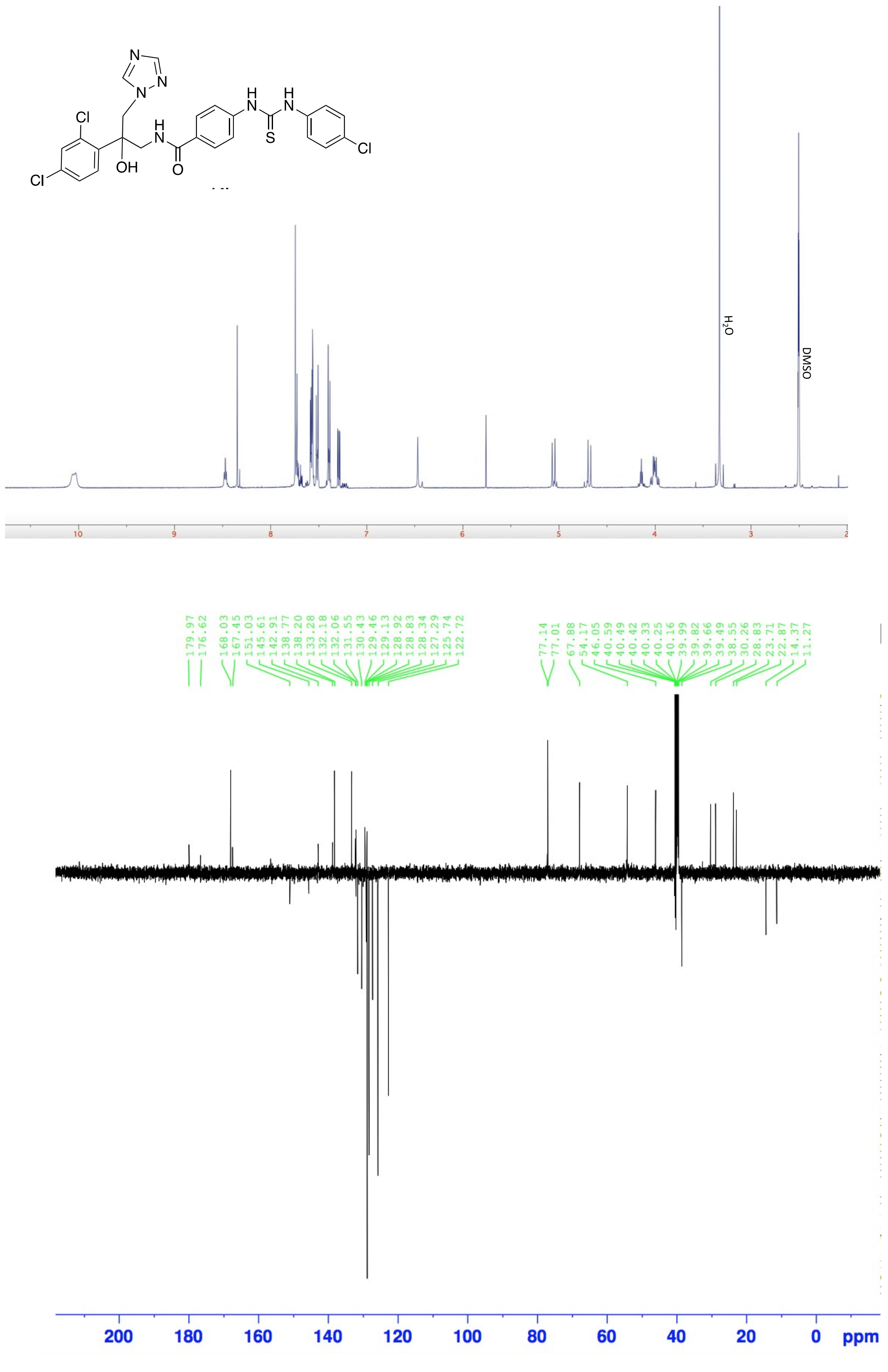

Figure S13. ^{1}H NMR and ^{13}C NMR of **12a**

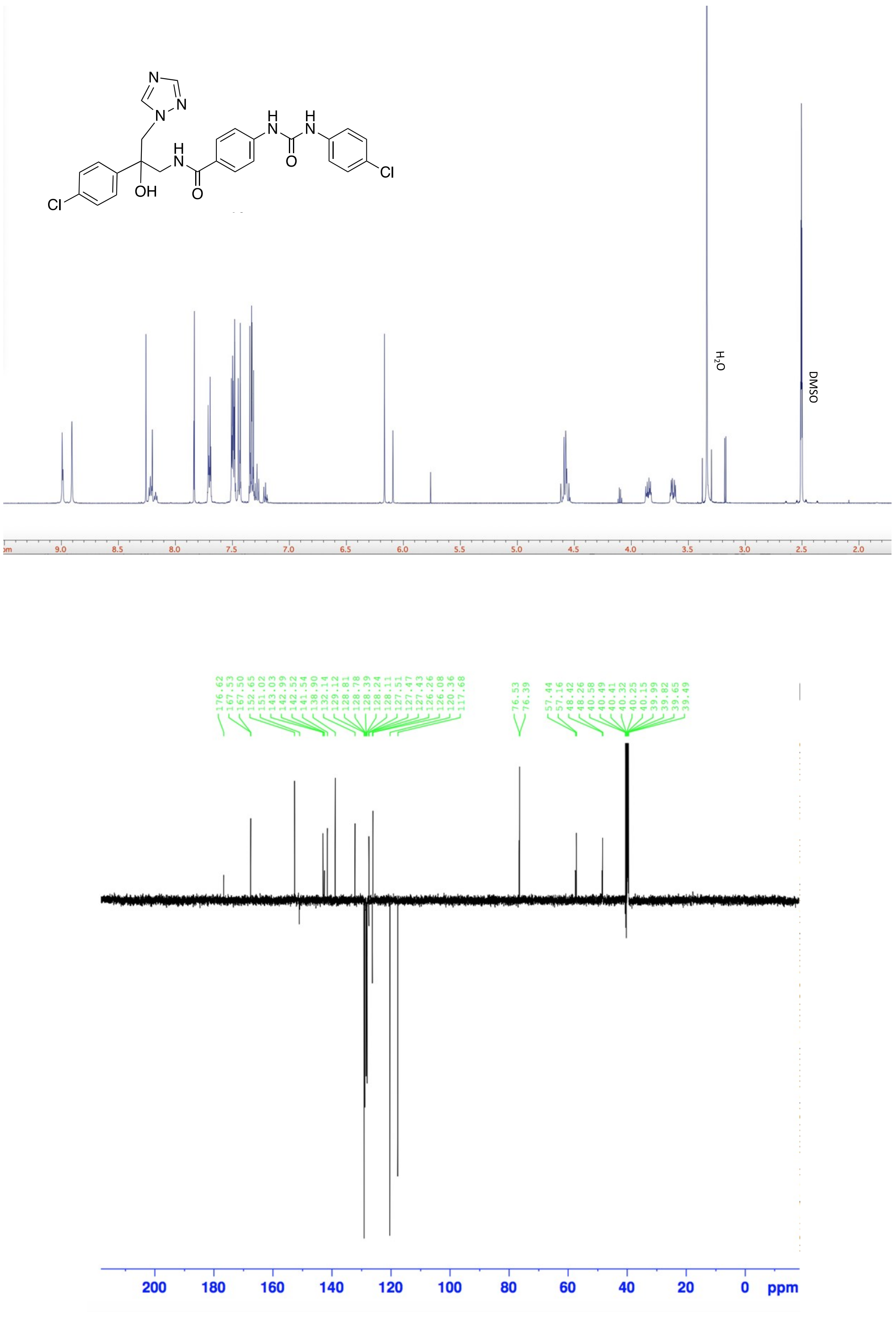

Figure S14. ^{1}H NMR and ^{13}C NMR of **12b**

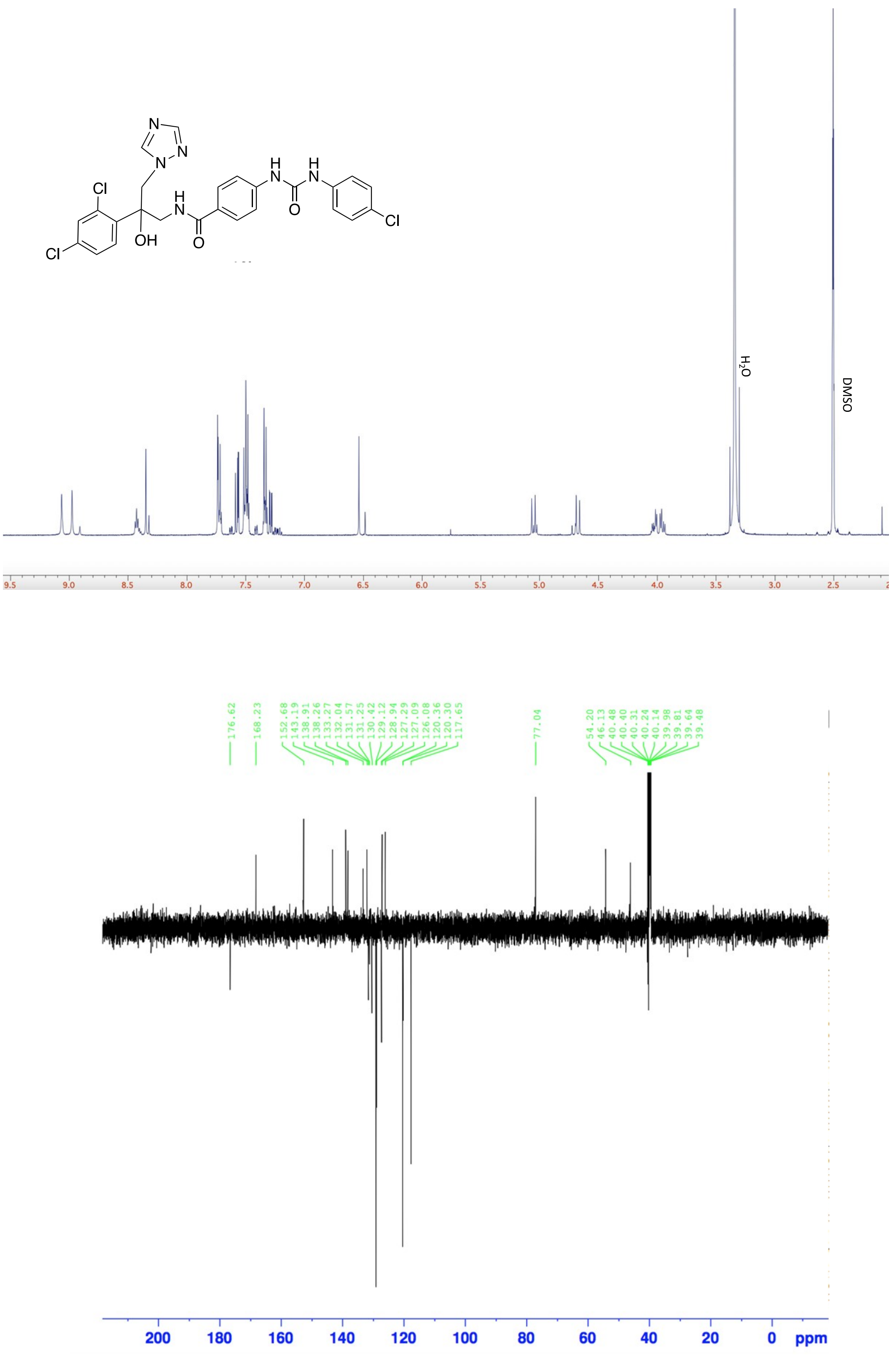

Figure S15. ^{1}H NMR and ^{13}C NMR of **13a**

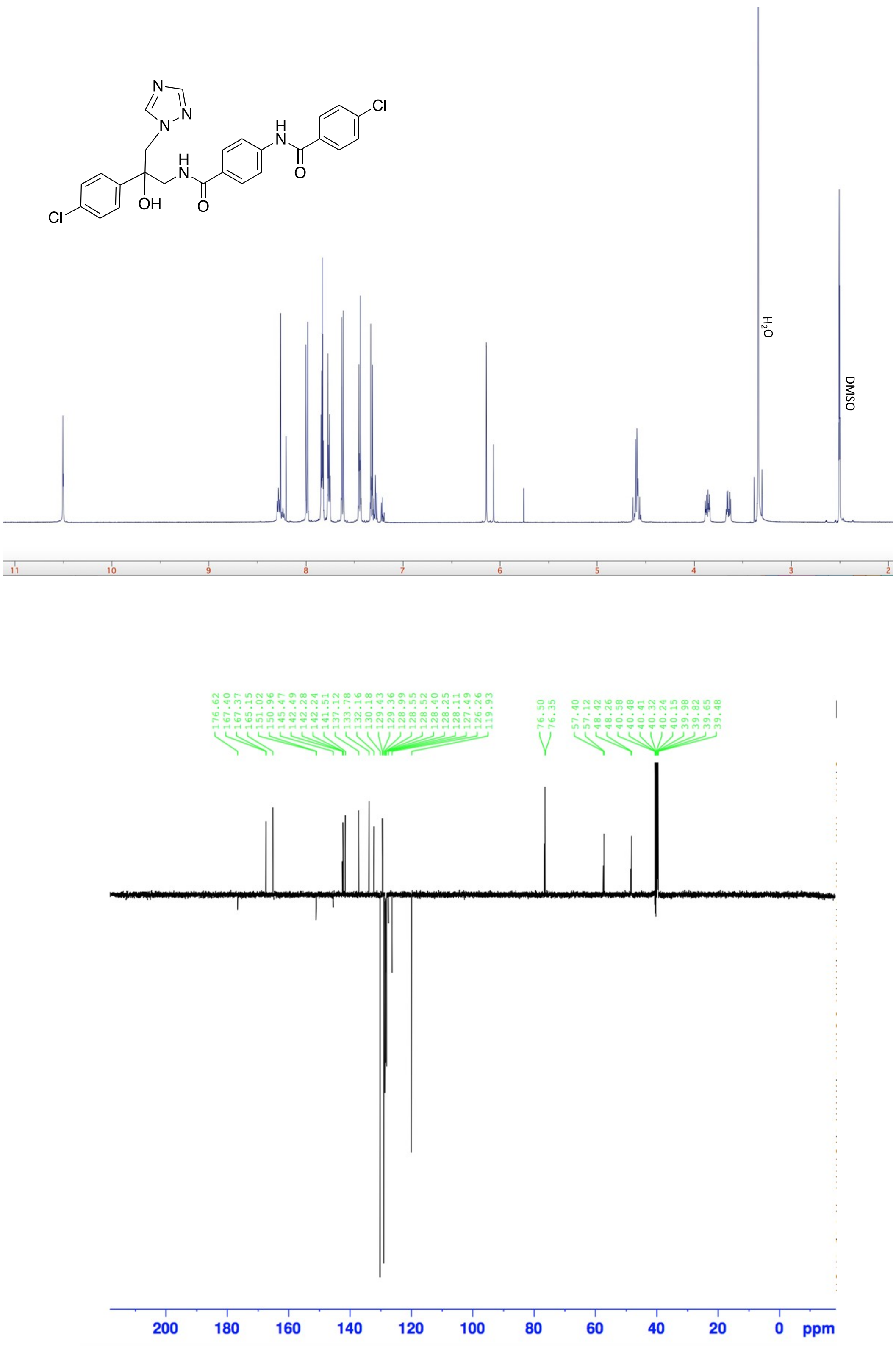

Figure S16. ^{1}H NMR and ^{13}C NMR of **13b**

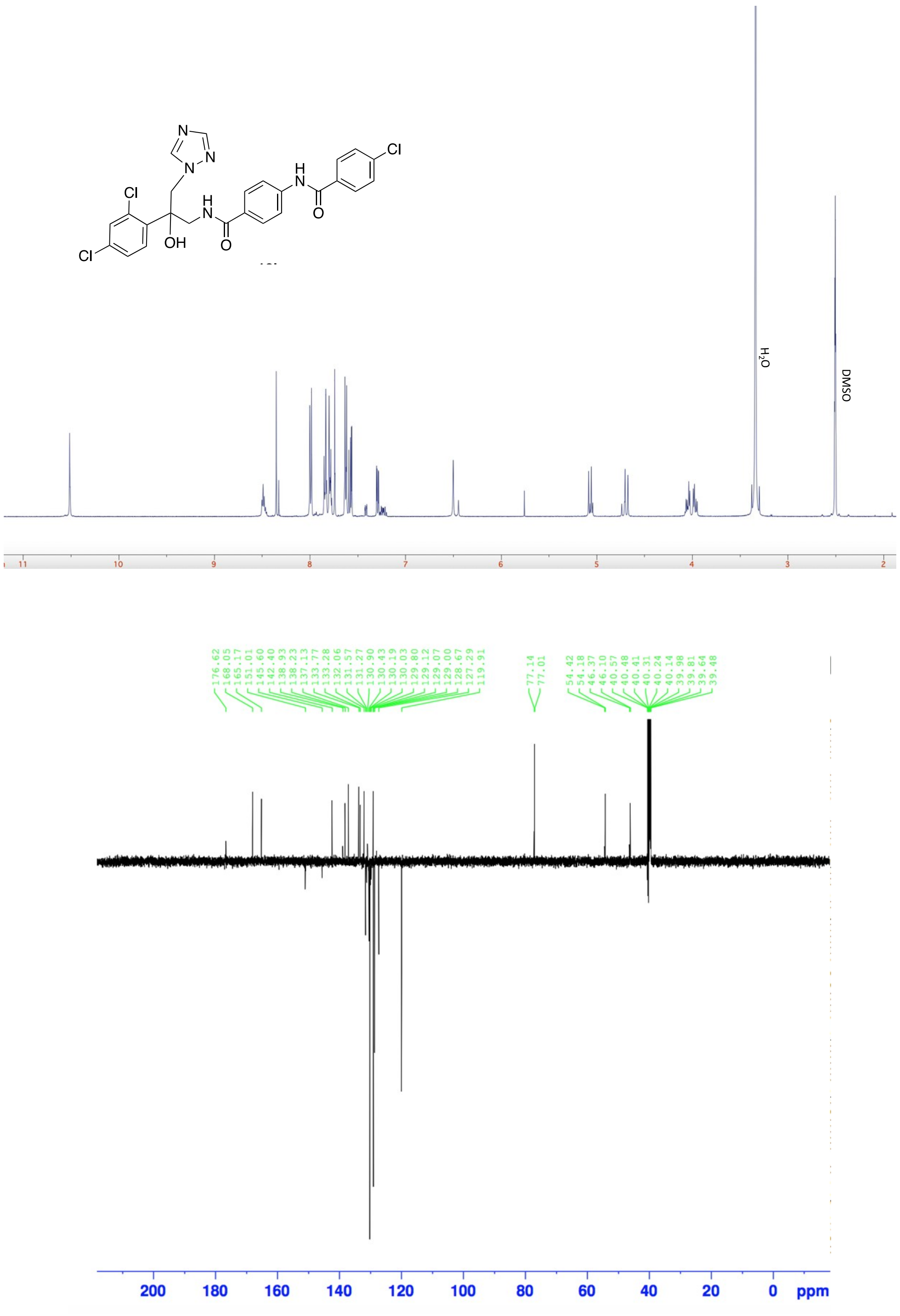

Figure S17. ^{1}H NMR and ^{13}C NMR and HPLC trace of **14a**

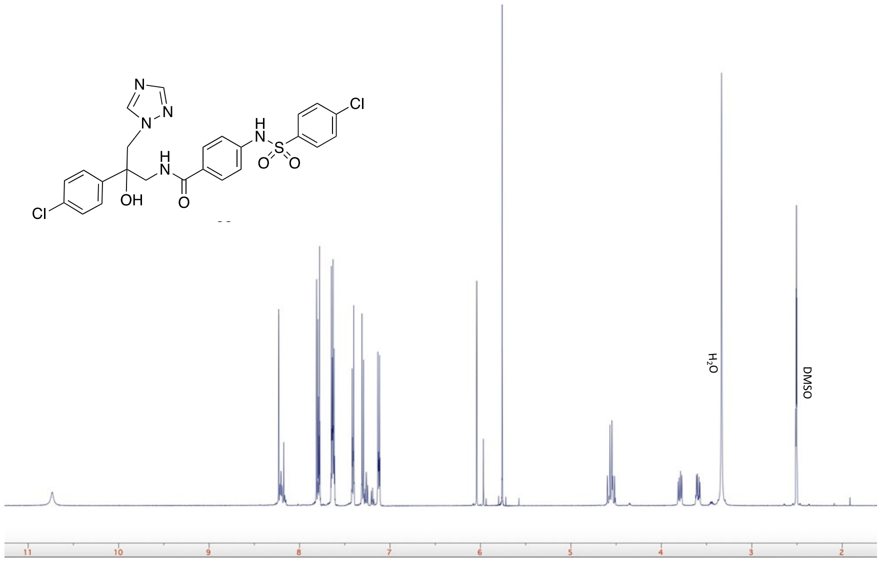

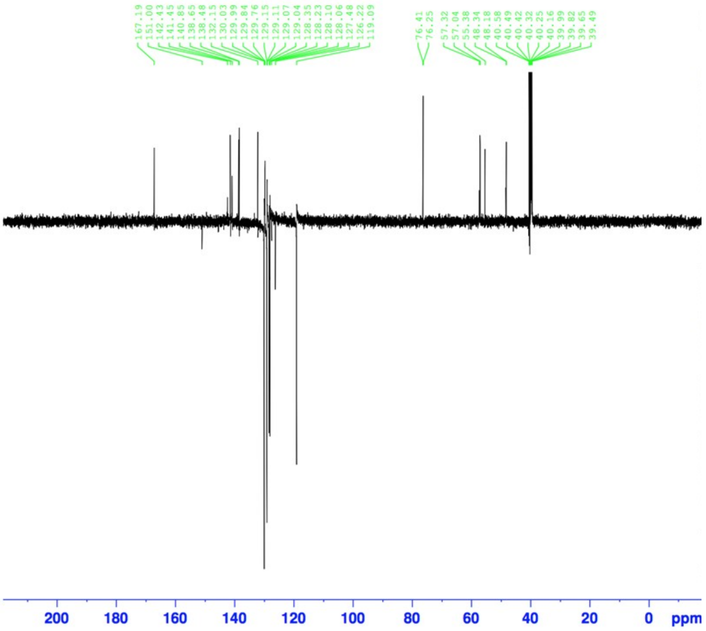

<Chromatogram>

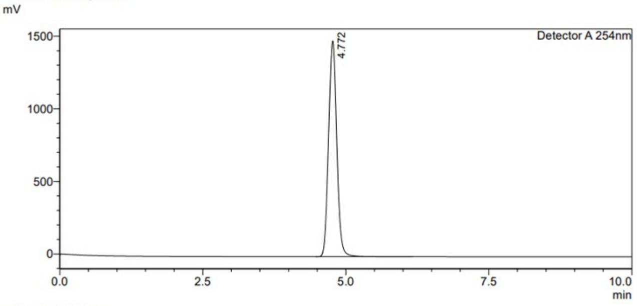

<Peak Table>

Detector A 254nm

Peak#	Ret. Time	Area	Height	Area%
1	4.772	14936639	1486206	100.000
Total		14936639	1486206	100.000

Figure S18. ^{1}H NMR and ^{13}C NMR and HPLC trace of **14b**

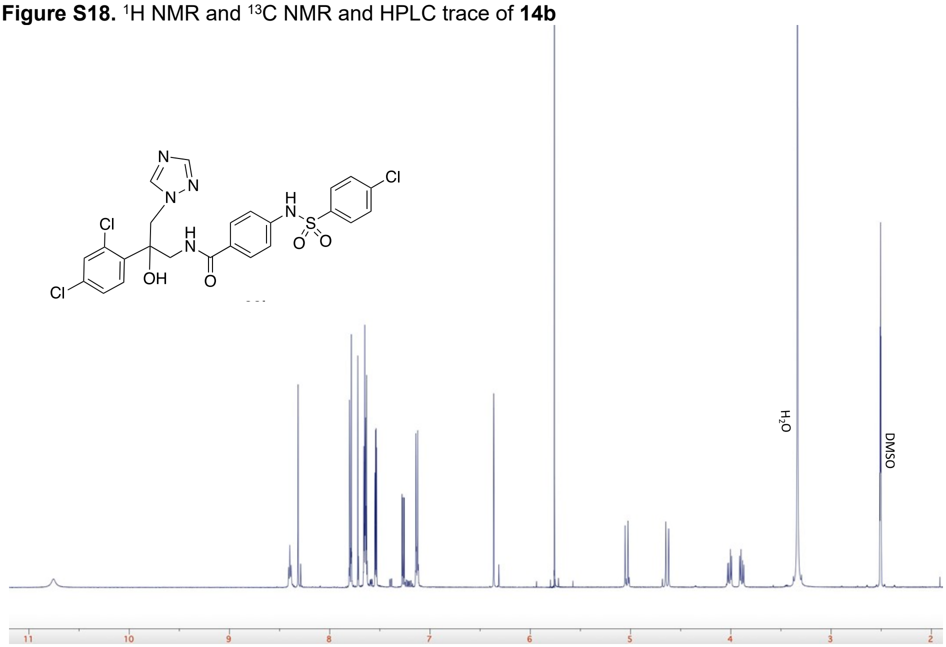

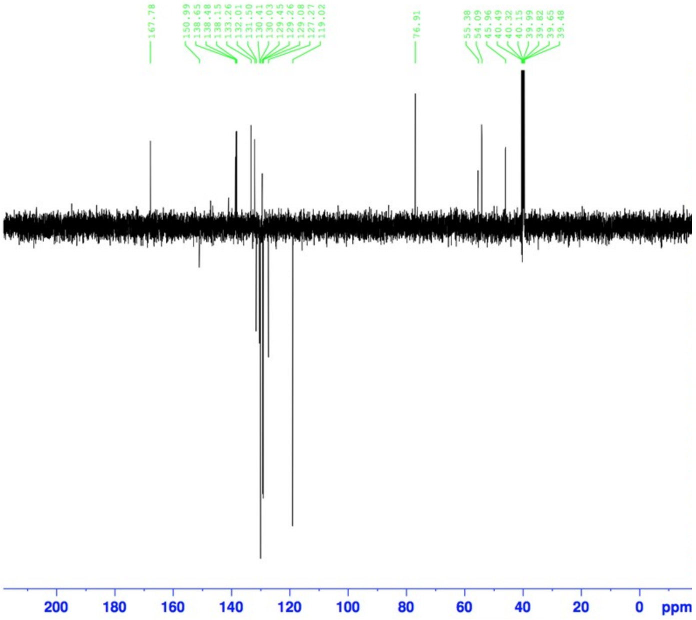

<Chromatogram>

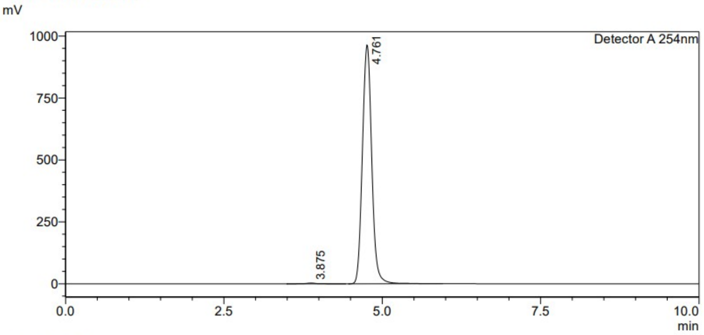

<Peak Table>

Detector A 254nm

Peak#	Ret. Time	Area	Height	Area%
1	3.875	30839	2954	0.309
2	4.761	9937208	963378	99.691
Total		9968047	966332	100.000

References

[1] Sekimata, K.; Han, S-Y.; Yoneyama, K.; Takeuchi, Y.; Yoshida, S.; Asami, T. J. A specific and potent inhibitor of brassinosteroid biosynthesis possessing a dioxolane ring. *Agric. Food Chem.* **2002**, *50*, 3486-3490. doi: 10.1021/jf011716w

[2] Astleford, B.A.; Goe, G.L.; Keay, J.G.; Scriven, E.F.V. Synthesis of 1-alkyl-1,2,4-triazoles: a new one-pot regiospecific procedure. *J. Org. Chem.* **1989**, *54*, 731-73. https://doi.org/10.1021/jo00264a048
